# Dual-Purpose Poultry in Organic Egg Production and Effects on Egg Quality Parameters

**DOI:** 10.3390/foods10040897

**Published:** 2021-04-19

**Authors:** Marianne Hammershøj, Gitte Hald Kristiansen, Sanna Steenfeldt

**Affiliations:** 1 Department of Food Science, Aarhus University, Agro Food Park 48, DK-8200 Aarhus, Denmark; ghk@food.au.dk; 2 Department of Animal Science, Aarhus University, Blichers Alle 20, DK-8830 Tjele, Denmark; sanna.steenfeldt@anis.au.dk

**Keywords:** layer hens, dual-purpose, egg, quality, shell strength, genotype, yolk color, egg weight, egg albumen

## Abstract

Egg laying genotypes have been selected for generations due to their high yield and egg quality, resulting in efficient feed utilization and low body weight; hence, they are not suitable for meat production. This imposes an issue for the male layer chicks, which are killed at one day old. Because of ethical and food waste concerns, the search for suitable dual-purpose genotypes in order to avoid euthanasia of male day-old chicks has intensified. The aim of the present study is to evaluate potential dual-purpose genotypes for their egg quality compared to a representative egg laying genotype. Two dual-purpose genotypes with divergent characteristics were evaluated: genotype A represented an experimental crossbreed based on a broiler type male and an egg layer female, and genotype C was a crossbreed of a layer type. These were compared to a rustic genotype B and a control genotype D, which was an egg layer. Eggs were collected six times during the period of 21–54 weeks of hen age, i.e., a total of 990 shell eggs were analyzed. Examined parameters were weights of egg, shell, yolk, and albumen, by calculating their relative proportions. Shell quality was assessed by shell strength, shell stiffness, and shell thickness. Yolk quality was determined as yolk color and inclusions of blood and meat spots, and albumen quality was evaluated in terms of pH and dry matter (DM) content. The egg layer genotype produced the smallest eggs with least blood and meat spot inclusions compared to that produced by the three dual-purpose genotypes. Shell quality was superior for the layer genotype. However, the experimental genotype A laid eggs of comparable shell quality, albumen DM, and yolk weight, but also with the darkest and most red-yellow colored yolk. The two other dual-purpose genotypes produced eggs of low-medium quality. In conclusion, the genotype A could serve as dual-purpose genotype from an egg quality perspective.

## 1. Introduction

Ethical and animal welfare concerns of consumers regarding husbandry procedures in poultry meat and egg production have been increased worldwide and especially in Europe [1,2]. One result has been the banning of battery cage egg production within EU countries, which came into action at the beginning of 2012. The egg production in the EU has changed in production system during the years. In 2016, 55.6% of EU egg production was in enriched cages, 25.7% deep litter production system, 14.1% free range, and 4.6% organic system [3]. The by-volume largest egg producing countries are Germany, France, and Spain, where >36% of all eggs in the EU are laid. There are large differences between member countries in the respective share of the production system, and, in 2016, enriched cages dominated the production in Lithuania, Spain, and Portugal (>90%), while having the lowest share in Austria, Germany, and Sweden (<15%). The highest shares of organic egg productions are found in Denmark, Sweden, Austria, and Germany (>10%), while very low in Lithuania, Latvia, Croatia, and Hungary (<1%) [3]. Although in certain markets white eggs have been associated with cage production systems, several European markets that have moved to cage-free production systems have also made the switch to hens laying white eggs. The Netherlands and Germany are examples of countries where this trend is happening [4]. Another animal welfare issue of poultry has arisen as a result of breeding strategies towards much differentiated poultry genotypes for many generations: meat-type broiler chickens with high growth rates and egg-type lean layer hens with low body weight and high egg-laying capacity. As a consequence, the male chickens of egg layer genotypes are not suitable for meat production due to their low muscle mass and slow growth rate, so they are killed immediately after hatching. This fact is associated with both ethical and economic issues in modern egg production as 50% of hatched eggs are wasted. A recent survey among 1000 German consumers shows that 67% of them find the practice of killing day-old chicks ‘very problematic’ [5]. As some countries, like Germany and France, have taken initiatives on a national level to ban the practice of killing of day-old male layer chicks by 2022 [6,7,8], solutions for handling the male layer chickens are necessary. Alternative strategies to the euthanasia of male layer chicks have been suggested, namely in ovo sexing, fattening of lay-hen males, and dual-purpose poultry [9]. Based on this perspective, it can be expected that there will be a growing interest in the use of dual-purpose genotypes in the future egg production, and that there will be an increased need for knowledge about the potential of these genotypes for both meat and egg production.

Dual-purpose poultry production uses less specialized genotypes for both egg and meat production, which are characterized by less efficient production [10], resulting in a lower profit for the farmer and a higher price of the products for the consumer compared to conventional egg-producing genotypes. However, the benefits of such strategies have not been assessed in the light of consumer/citizen expectations or in comparison to slow-growing broilers reared outdoors. Most consumers, e.g., 82% of German consumers [5], are not familiar with dual-purpose poultry, i.e., both meat and egg production can be carried out by the same genotype.

Furthermore, the egg production and consequently the quality of eggs laid by alternative dual-purpose genotypes has not been evaluated, with regard to the retail shell egg quality conceived by consumers. Many generation breeding strategies of modern egg layers have focused on egg quality to provide eggs of improved shell quality, i.e., strength and thickness; high albumen quality, i.e., high protein content for human nutrition and in food textures as gels and foams; and increased yolk proportion of the egg to provide nutritious compounds as vitamins, minerals, fatty acids, and carotenoids [11,12] 

At the end of the day, the dual-purpose production on the egg side is challenged by low output and thus a higher egg retail prize. This demands a positive consumer attitude and willingness to pay [13], where an essential key issue for the consumer is to receive a food product of a high quality or at least of same level as the traditional well-known egg-layer egg quality.

The aim of the present study is to examine the effect of using genotype breeding strategies for dual-purpose chickens on egg production and on various retail egg quality parameters. We hypothesize that dual-purpose poultry can be used for organic egg production and lay eggs of a comparable quality to those of an egg layer genotype.

## 2. Materials and Methods

### 2.1. Materials

Eggs were produced from four different genotypes in the study, including two dual-purpose genotypes (A, C), a rustic breed (B) and a commercial egg layer (D). The dual-purpose genotypes with divergent characteristics, and a rustic genotype were selected by the French Poultry and Aquaculture Breeders Technical Center (SYSAAF) in cooperation with two breeding companies. Genotype A represented an experimental cross breed based on a broiler type male and an egg layer female laying brown-shelled eggs, where genotype C was a cross breed of a layer type laying brown-shelled eggs. Genotype B represented a genotype that has not been selected for any specific traits and included to compare with the dual-purpose genotypes orientated more on meat or eggs production. Finally, genotype D was a control egg layer breed laying white-shelled eggs (purchased at a local pullet breeder). However, due to a mistake in the chick delivery for genotype A, fewer chickens than expected were available for genotype A, which resulted in only two replicate units for genotype A instead of three replicates that was planned. For genotype B, C and D, there were three replicate units.

All genotypes received the same starter and grower diets until 19 weeks (week) of age. From 19 to 31 week of age, two different starter layer diets were given, one (I) for the light layer type (control group) and another (II) for the three dual-purpose genotypes receiving the same diet with a lower protein content. (I): protein: 18.3%, MJ ME: 11.2, methionine: 3.3 g/kg, lysine: 9.3 g/kg. (II): protein: 17.3%, MJ ME: 11.2, methionine: 3.1 g/kg, lysine: 8.7 g/kg). From 32–42 week and 43–62 week, a layer phase 1 and 2 was offered, where the protein content was reduced to 17.2 and 16.5% (I) and to 15.1% (II), respectively. The amino acids were reduced accordingly. MJ ME was 11.2/11.0 (I) and 10.9 and 11.0 (II), respectively. Calcium content was the same in both layer diets I and II (3.5–4.0%) and phosphorous content was on average 0.67% (I) and 0.60% (II). Values are presented ‘as is’. All diets were based on organic ingredients and no crystalline amino acids were added.

The stocking density was 4 m^2^ per hen on the outdoor area as stated in the legislation for organic laying hens to be the minimum area available in organic poultry production. There were rows of willow on each outdoor unit and an open space between the willows with 3 mobile houses of 2 × 3 m each. All houses were equipped with perches, a feeding trough (40 L), and a round trough for water supply. Each house had 5–9 nest boxes, which could be reached from inside by the hens and from outside for collection of eggs. Nest space and perch length per hen followed the legislation for organic laying hens.

Six times, at the hen ages of 21, 25, 30, 38, 46, and 54 week, 15 eggs were collected from each of the 11 outdoor units with mobile houses, representing the four genotypes in two (A) and three replicates (B, C and D), i.e., a total of 990 shell eggs were individually analyzed. 

The eggs were stored at 22 °C until analysis. On day 1 after egg collection, the 165 eggs were marked and individually weighed; any visually cracked eggs were removed. For practical reasons in order to overcome 165 egg samples, the parameter analysis was distributed over several days. On day 5, egg-shell strength analysis was performed, and on day 7 eggs were broken, day 8 the albumen dry matter was recorded, and day 9 dried egg shells were weighed and shell thickness measured.

### 2.2. Methods

#### 2.2.1. Shell Quality Parameters

Eggs were subjected to shell strength measurement as described earlier [14]. The recordings of force and displacement data at the fracture of the shells resulted in the parameters of shell strength (N) and shell fracture point (mm). The shell stiffness (N/mm) was defined as the slope of the initial part (0.01–0.03 mm) of the force–displacement curve and resulted in the stiffness parameter of the egg shell [15]. Furthermore, the diameter of eggs, i.e., initial height (mm), were obtained by the analysis, and was used to calculate the percentage ratio of egg compression before fracture given as ‘shell-to-egg compression’ = shell fracture point (mm)/initial height (mm) × 100 (%) s.

After breaking the eggs for yolk and egg albumen analyses as described below, the egg shells were washed in lukewarm running water and set to dry at room temperature for 48 h, after which the shell weight was recorded. The shell thickness (µm) was measured around the equator of each egg in triplicate by a micrometer (Disella A/S, Kolding, Denmark) with a round tip and 1 µm accuracy. 

#### 2.2.2. Yolk Quality Parameters

Eggs were broken and the yolk and albumen separated by cutting the albumen free with a scalpel. Any visual blood spots and meat spots were noted, and egg albumen remains on the egg yolk were removed by rolling the egg yolk carefully on a paper tissue. The egg yolk color was measured by a Minolta Chroma Meter CR-300 with an 8 mm diameter measuring area (Minolta Co. Ltd., Osaka, Japan) using the CIEL* a* b* (Commission Internationale de L’enclairage, Vienna, Austria). The lab scale includes the three parameters of lightness L*, where 0 = black and 100 = white, redness a*, where −100 = green and 100 = red, and yellowness b*, where −100 = blue and 100 = yellow. The calibration was performed on a predefined white plate (no. 19833046) with standardized daylight (D65) and Y, x, y values of 93.4, 0.3158, and 0.3324, respectively. Hereafter, the weight of each individual egg yolk was recorded.

#### 2.2.3. Egg Albumen Quality Parameters

The egg albumen was collected in a 50 mL-beaker glass and homogenized by using an Ultra Turrax fitted with a 0.5 cm diameter homogenizer at a speed of 8000 rpm for 20 s. A subsample (~2–3 g) of the homogenized egg albumen was transferred into a porcelain pan for determination of dry matter (DM) content by drying in a heating cabinet at 98 °C for 18 h and reweighed as dry. The DM (*w*/*w*-%) was calculated as ‘dry sample weight’/‘wet sample weight’*100. Another subsample of homogenized egg albumen was used for pH measurement carried out by a pH-meter MeterLab^TM^ PHM220 (Radiometer, Copenhagen, Denmark) calibrated with IUPAC certified buffer standard solutions of pH 7.00 and pH 10.01 (HACH Lange GmbH, Berlin, Germany). 

The weight of the egg albumen was calculated by subtracting the weights of egg yolk and egg shell from the ‘egg weight’, and the relative (%) proportions of yolk, shell, and albumen were calculated.

#### 2.2.4. Data Analysis

A two-way analysis of variance (ANOVA) with class variables of four genotypes (A, …, D) and 6 hen ages (21, …, 54 weeks) with 2–3 replicate outdoor units (1, 2, 3) of 15 eggs analyzed per unit was included as model with interactions between age and genotype.

Data distribution of continuous data, i.e., all but meat spot and blood spot, was checked for normality by the PROBIT function, and variance homogeneity by a Bartlett test using the software program SAS version 9.3 (SAS Institute Inc., Cary, NC, USA). Only the shell stiffness data did not show normal distribution, and the data were then transformed by a logarithm function (log x) to obtain normal distribution.

The following model including overall mean (µ), main effects, and interactions between these was applied for all egg parameters, (17 in total), Y = µ + a (genotype 1–4) + b (hen age 1–6) + a × b + e.

When the interactions between genotype and age were not significant, i.e., *p* > 0.05, they were excluded from the model; Y = µ + a (genotype 1–4) + b (hen age 1–6) + e, which was the case for albumen pH, shell strength, shell-to-egg compression, shell stiffness, shell thickness, yolk percentage, and albumen percentage. Least Squared Means (LS-means) were considered significantly different at minimum 95-% level (*p* ≤ 0.05).

Pearson correlation was calculated at 30 week of hen age of egg yolk color a* in relation to the outdoor unit vegetation coverage as given in Figure 4B. Individual data are available in Supplementary Table S1, where traits are whenever possible presented in reference to ontology ATOL: https://www.atol-ontology.com/en/atol-2/ (accessed on 2 December 2021).

## 3. Results

**Figure 1:**
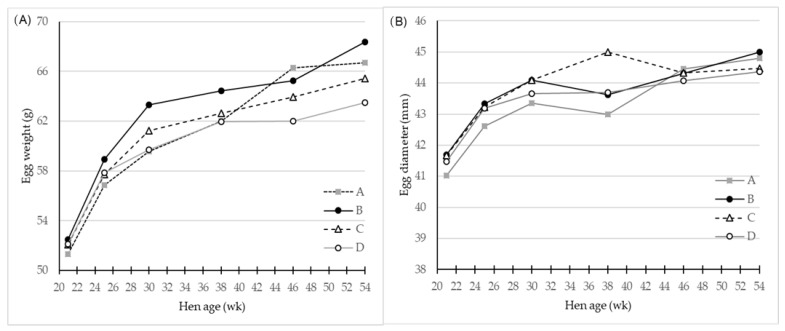
LS-means of (**A**) Egg weight (g) and (**B**) egg diameter (mm) for eggs of four different hen genotypes A, B, C, and D as differentiated by hen age (week), *n* = 30 for genotype A and 45 for genotypes B, C and D. There was a significant (*p* < 0.001) interaction effect of genotype and hen age on both parameters.

The egg quality was assessed quantitatively as egg weight (Table 1), and highly significant effects of both genotype and hen age were found (*p* < 0.001). Hen age was observed to be positively associated with egg weight, with the major increase during the first part of the egg laying period (Figure 1A).

The genotype B laid eggs that were significantly heavier than the three other genotypes. The egg weight from genotype A was no different from those of genotype C, but both laid egg of significantly higher weight than eggs of genotype D. Eggs from genotypes C and D were no different in mean egg weight. There was a significant interaction of genotype and age as shown in Table 1 and the egg layer genotype D had a more steep egg weight curve initially, which flattened as hens grew older (Figure 1A). This was not the case for genotype A, which had lower egg weight at the beginning of laying period, but at 46 week these eggs had the highest weight. Genotype B generally produced eggs of the highest egg weight throughout the total period, and genotype C eggs had a weight within that of the three other genotypes.

Generally, the egg diameter increases as eggs get larger (Table 1, Figure 1B), and eggs from genotype B and C had greatest values (*p* < 0.001) for diameter compared to egg diameters of genotype A and D.

The presence of blood spots and meat spots in the eggs was calculated as frequency of eggs having one or more of these spots. Only the genotype had a significant influence on these parameters. In total, the eggs originating from genotype B had significantly higher frequencies: 25.2% for blood spots (*p* < 0.001) and 15.9% for meat spots (*p* < 0.01), while genotype D had the lowest frequencies of 0.7% and 5.2%, respectively (Table 1).

**Figure 2:**
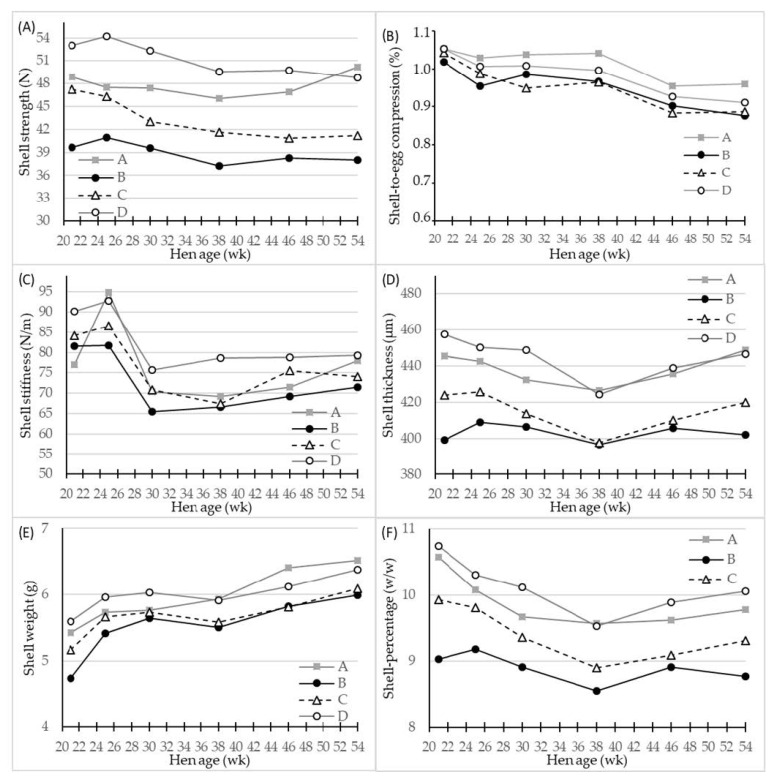
LS-means of shell parameters of four different hen genotypes (**A**–**D**) as differentiated by hen age (week). (**A**) shell strength (N) at compression, (**B**) shell-to-egg-compression (%), (**C**) shell stiffness (N/m), (**D**) shell thickness (µm) at equator, (**E**) shell weight (g) after air drying, and (**F**) shell percentage (*w*/*w*), *n* = 30 for genotype A and 45 for genotypes B, C and D.

All the shell quality parameters were significantly affected by hen age (*p* < 0.001) and genotype (*p* < 0.001), while significant interactions between age and genotype were found only for the shell thickness, shell weight and shell percentage (*p* < 0.01–0.05) (Table 2, Figure 2). Overall, the egg layer genotype D had the significantly highest values of all shell parameters, apart from the shell-to-egg compression, where genotype A laid eggs that had a higher value (Table 2), while the genotypes B and C produced eggs with inferior shell quality parameters.

The effect of hen age on the shell quality parameters is shown in detail in Figure 2 for the four hen genotypes. All shell quality parameters decreased with hen age apart from the shell weight, which was increased (Figure 2E); however, as a consequence of an even higher increase in egg weight (Figure 1A), the relative proportion of the shell of the egg also decreased as hens grew older (Figure 2F). The genotypes B and C were inferior in shell quality during the whole egg production period. The results are more straightforward for genotype B, since the values of shell strength, shell thickness, and shell percentage did not at any time during egg laying period reach a level comparable to that of genotypes A and D.

**Figure 3:**
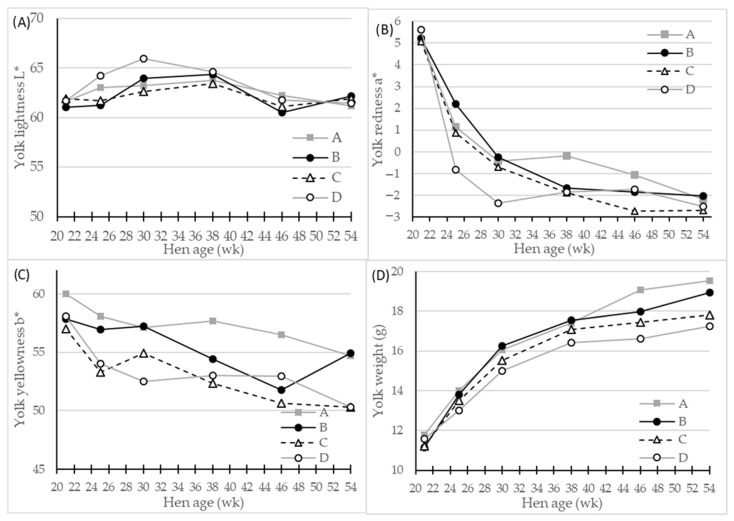
LS-means of egg yolk color parameters and egg mass of four different hen genotypes (**A**–**D**) as differentiated by hen age (week). Panels showing (**A**) L* (lightness), 0 = black, 100 = white, (**B**) a* (redness), −100 = green, 100 = red, (**C**) b* (yellowness), −100 = blue, 100 = yellow, and (**D**) egg yolk weight, *n* = 30 for genotype A and 45 for genotypes A, B and C, significant interactions of hen age * genotype (*p* < 0.01) for all parameters.

The egg yolk quality assessed as yolk color parameters and yolk mass (g and %) were all significantly affected by genotype (*p* < 0.001) and hen age (*p* < 0.01 or *p* < 0.001) (Table 3). The egg layer genotype D had significantly fewer red, and fewer yellow egg yolks compared with the genotypes A and B, and the genotype A generally had the most red and yellow egg yolk color. The yolk color was further affected by hen age interacting significantly (*p* < 0.01 or *p* < 0.001) with the genotype (Table 3). The lightness L* and yellowness b* parameters fluctuated as hen age increased with a general trend of slightly decreasing yellowness (Figure 3C). The redness a* values were initially very high at beginning of laying period (Figure 3B), and decreased significantly as hens got older. From 24–38 weeks of age, different values for yolk redness between the genotypes A and D were observed, while egg laying genotype D had the lowest values for redness and genotype A had the highest (Figure 3B). The egg yolk mass increased by hen age, as expected, with highly significant (*p* < 0.001) genotype differences of >1 g on average between genotypes A and D (Table 3). This genotype difference persisted throughout the total experimental period, and became more pronounced as hen age increased (Figure 3D), with a peak at 54 week in egg yolk weight from 17.2 g of genotype D eggs to 19.5 g of genotype A eggs.

**Figure 4:**
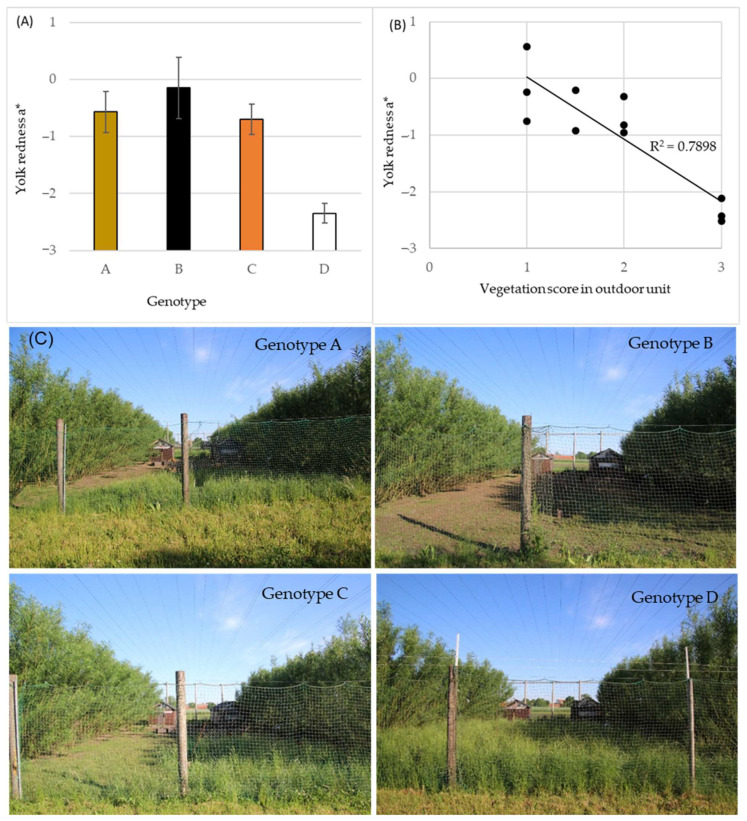
(**A**) LS-mean egg yolk color redness a* at hen age 30 week for four hen genotypes A–D, *n* = 30 for genotype A and 45 for genotypes B, C and D, vertical bars indicate standard deviations. (**B**) Correlation of scores for visual evaluation of vegetation coverage in outdoor units of organic poultry 3 weeks prior to egg collection and yolk color redness a* of panel A data. Each data point represents one pen and 15 egg yolk colors. Score 1 = mainly bare soil, 2 = partly covered with vegetation, 3 = full covered with vegetation. (**C**) Representative photos of outdoor units for hen genotypes A–D at time for vegetation scoring.

The significant difference in egg yolk color among the genotypes (Table 3, Figure 3) was further evaluated based on the supply of green vegetative material in the outdoor area of the organic experimental facility (Figure 4). The values of yolk color redness at 30 week of hen age was shown to correlate negatively (r = −0.889) with the visual grading score of vegetation coverage in the units (Figure 4B). Higher egg yolk redness * was observed in eggs from hens in units where vegetation coverage score was low, i.e., the hens had foraged more actively than in units with high full vegetation coverage, which showed results of lower values of yolk redness a*. For illustrative purpose, photos of the outdoor units’ vegetation at 3 weeks prior to the egg sampling at 30 week of hen age can be observed in Figure 4C. 

The egg albumen quality was evaluated by pH value and DM content (Table 4). The albumen pH did not differ among the genotypes. On the other hand, the DM content, which reflects mainly the protein content of albumen, was significantly higher for eggs of genotype A, which also had the relatively lower proportion of albumen by mass of the whole egg, compared to the other genotypes. This resulted in the most concentrated albumen regarding DM of eggs from genotype A. Eggs from genotype B and D had significantly lower values for albumen DM content, and genotype B had the greatest albumen relative weight. 

**Figure 5:**
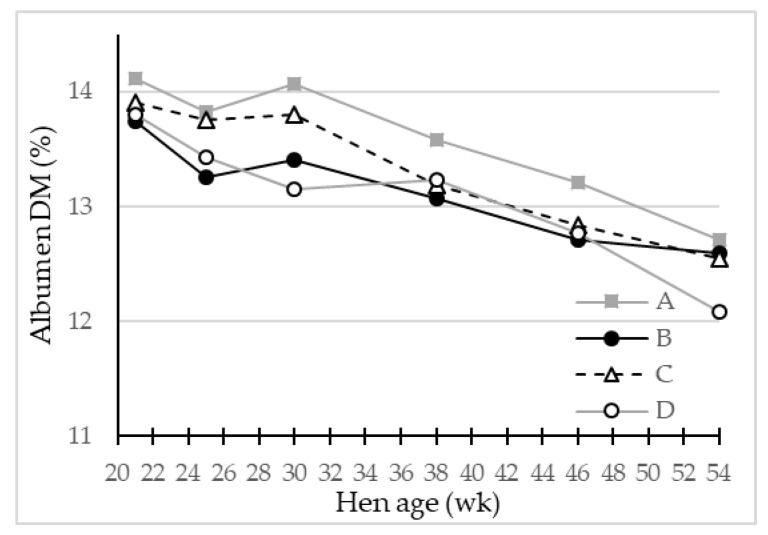
LS-means of egg albumen dry matter (DM) (%) in eggs from four different hen genotypes (A–D) as function of hen age (week) (*p* < 0.05), *n* = 30 for genotype A and 45 for genotypes B, C and D.

Based on the LS-means of egg weight, albumen-% and albumen dry matter relative weight of the eggs (Table 4, Figure 5) from the four genotypes, the produced mass of albumen dry matter per egg was calculated on average to be 5.06 g/egg for egg layer genotype D, 5.20–5.27 g/egg for genotypes C and A, while genotype B eggs contained the overall highest albumen dry matter of 5.36 g/egg, mainly caused by increased egg weight and albumen weight.

## 4. Discussion

Implementing dual-purpose poultry in modern egg production is facing a challenge in exchanging the egg layer genotypes, which for many generations have been intensively bred for high number of eggs, high feed efficiency, low bodyweight, and high egg quality. These parameters are not at the same high levels in the dual-purpose poultry. In order to implement dual-purpose genotypes in egg production, it is necessary to identify genotypes that among other production criteria have high egg qualities, which here are considered as shell strength, yolk-ratio, dry matter of egg albumen, and absence of blood and meat spots.

### 4.1. Egg Weight, Proportions, and Inclusions

One of the most important parameters in modern egg production from the farmers’ perspective is the laying rate together with the egg weight that provides the value of egg mass produced. Egg layer genotypes have for generations been selected for a fast increase in egg weight after onset of lay followed by a steady level of egg size or only a slight increase in egg weight for a one-year production period [16,17,18]. The egg weight curve of the egg layer genotype D reflected this breeding goal, while the three dual-purpose geno-types demonstrated a lower egg weight at the beginning of lay and a higher egg weight at 36 week to 54 week of age compared to the egg layer type (Figure 1, Table 1). The egg weight increase in genotype D from week 24 to week 54 was 5.4 g corresponding to a 9% increase, a value that for the genotypes A–C ranged from 7.7–9.8 g, which corresponded to 13–17% increase, with genotype B having the highest values. The egg quality may be negatively affected by a too high egg weight increase if the synthesis of calcified egg shell mass and protein in egg albumen cannot follow this increase [19]. Furthermore, the laying rate could also be expected to be lower in the genotypes A–C compared to genotype D, and as result the total egg mass output.

In a recent study from north Italy, two purebred genotypes of higher bodyweight produced eggs of lower weight compared to that of two hybrid egg layers (HyLine Brown and HyLine White) during ages of 28–44 week [20], which is in contrast to the present study with dual-purpose genotypes. The size of eggs produced mainly depends on the genotype, as crossbreeds of Naked Neck with either Rhode Island Red or with Black Australorp lay heavier eggs than the purebred hens of Nacked Neck [21].

When looking at the edible egg proportions; the egg yolk:egg albumen (w:w) was much higher for the genotype A with a ratio of 0.42 than the other three genotypes with ratios of 0.38–0.39. The eggs within genotype A contained on average 0.8 g more egg yolk and 1.2 g less egg albumen compared to the mean of eggs from genotypes B–D. In detail, the higher yolk:albumen ratio of genotype A was induced by differences in both yolk and albumen proportion. Even though genotype A laid larger eggs than genotype D, the yolk weight was also higher and also increased with age at a higher rate. This finding is in contrast to the previous literature, where larger eggs typically have relatively lower proportion of yolk, both when egg size differs between genotypes [22] and increases due to hen age [23,24], as was observed for eggs of genotype B, which laid the overall heaviest eggs with less yolk weight and relative proportion.

This finding may be very relevant from a food perspective, when using eggs as component in complex foods, where either the egg yolk or the egg albumen is the main ingredient. 

The inclusion of blood spots in eggs is a natural phenomenon occurring during ovulation, when the follicle is ruptured and a small blood hemorrhage resides, which may enter the oviduct together with the follicle and reside with the egg yolk. Furthermore, during albumen synthesis in the oviduct, the meat spots, which can be either shell pigments, blood coagulum, or tissue, reside with the egg albumen [25,26]. Their level was very low in the eggs from layer genotype D (5.2 and 0.7%, respectively), while in the dual-purpose genotypes A–C higher meat (13–16%) and blood (4–25%) spot frequencies were observed and have been ascribed to a lack of genetic selection in breeding to avoid them. The blood and meat spots are of aesthetic concern for the consumer, although completely harmless from a food safety view. In particular, genotype B showed a consistently high level of blood spots throughout the experimental period varying from 11–35% of eggs, which corresponded to another study where purebred dual-purpose genotypes showed higher levels of blood and meat spots than egg layer genotypes, and particularly white shelled layers (HyLine White) had overall low values of these parameters [20]. The exact numbers of blood and meat spots should be assessed with care, as it is a subjective parameter evaluated by the eye, and very small spots may not be detected.

### 4.2. Overall Egg Quality and Hen Age

Performance and egg quality parameters (egg weight and shell quality) of a modern dual-purpose genotype (Lohmann Dual) were compared with traditional dual-purpose genotypes and a layer genotype (Lohmann Brown Plus) from onset of lay until 33 week of age [27]. The egg laying rate, egg weight, and egg shell quality were in general worst with the traditional dual-purpose genotypes, while egg quality of the modern dual-purpose genotype was within that of the layer genotype, although number and size of eggs were inferior compared to that of the layer genotype [27]. Depending on the dual-purpose breeding strategy and which egg quality parameters are in focus, discrepancies are to be expected between genotypes.

We also observed great variability in the quality attributes between the genotypes A, B, and C when compared to the egg layer genotype D. The genotype A generally produced eggs of comparable shell quality, higher yolk mass, more red-yellow colored yolk, higher albumen DM, and higher egg weight with greater inclusion of blood spots and meat spots compared to the layer genotype D. The genotype B was in general most inferior regarding these egg quality parameters, when compared both to the egg layer genotype D and to the two other dual-purpose genotypes A and C. Finally, the genotype C presented egg qualities that were generally inferior to the eggs of genotypes D and A, but superior level than that recorded for genotype B.

In several studies, egg quality was evaluated over a shorter production period, e.g., 28–44 week of age [20], up to 33 week of age [27], and 25–44 week of age [28], although it is well known that egg quality parameters, such as shell, yolk, and albumen are modified generally towards lower quality as hens grow older [18]. A major strength of the present study was the analysis of egg qualities of dual-purpose genotypes at a higher hen age, i.e., from 21 to 54 weeks. For all egg quality parameters, the hen age effect was as expected, while there were significant interactions between genotype and age for some of them. As indicated, the dual-purpose genotypes in comparison to the egg layer genotype D had a higher increase in egg weight at older age. For shell strength parameters, all genotypes showed the same tendencies with hen age, although for shell weight and shell percentage a significant interaction was observed as genotype A resembled the pattern of the egg layer genotype D with age, while the curves as function of age for genotypes B and C differed. Finally, the dual-purpose genotypes had yolk weights that increased with age to a higher extent, and the egg albumen DM decreased less at high hen age than of the egg layer genotype D.

### 4.3. Shell Quality

It is well known that the deterioration of egg shell quality with age increases the incidence of cracked eggs due to a decrease in shell thickness, strength, and stiffness [15]. Naturally, it is important to keep the number of cracked eggs low and shell quality high both for reasons of food waste and for microbial safety, i.e., affecting the economy of egg production. The higher shell stiffness of egg layer genotype D would predictively result in lower numbers of cracked eggs [15]; however, the genotype A seemed to maintain a reasonable shell stiffness during the experimental period, and the shell thickness of the genotype A was at a numerical high level although significantly different from genotype D. Nevertheless, many other factors have an impact on the risk of egg cracking, e.g., egg handling, stress factors, diseases, dietary supply of calcium and phosphorous [29,30], and production system [31], which in the present study were similar among the genotypes. Housing systems of organic, free-range, and litter production systems are, in comparison with enriched cages, found to result in eggs with thicker egg shells [32,33], which is suggested to be due to an effect of environments which encourage hens to be more physically active [34], which with all other factors equal, has a positive impact on bone strength and calcium resorption to egg shell mineralization [35]. However, there are interactions between housing system and genotypes regarding egg shell quality.

Breeding of egg laying hens has used shell quality among the genetic selection goal for decades; hence, it is expected that egg layer genotypes lay eggs superior in shell quality compared to less genetically selected genotypes [36]. Dual-purpose crossbreeds of Nacked Neck and Rhode Island Red produce eggs of higher shell thickness than that of the purebred of Nacked Neck [21], which is possibly related to the genotypes used in crossbreeding. The genotype A in the present study was a representative of a crossbreed of male broiler and female egg layer.

### 4.4. Yolk Quality

In the study by Rizzi et al. [20], it is reported that the yolk-% of eggs of the purebred genotypes is higher (29.1% on average) than that of the egg layer genotypes (25.4% on average) [20], which is similar to the findings with the egg layer genotype D yolk-% being 24.9% and the dual-purpose genotypes A–C yolk-% of 25.8% on average, as the yolks of genotype A in general were darkest. 

Surprisingly, we found a significant difference in egg yolk color parameters L*, a*, and b* among the four genotypes, since the yolks of genotype A were darkest and most red and yellow, yolks of genotypes B and C of intermediate color, and those of genotype D were palest and least red and yellow. As the pigments for egg yolk coloring comes solely from the dietary intake of carotenoids originating from plant material or marine products [37,38,39,40,41], and since all hens in the study had identical housing, diets, and outdoor access, this difference in egg yolk color was unexpected. 

Hens of all genotypes were quite active in using the whole outdoor area, but the genotypes displayed quite different behaviors regarding foraging on the vegetation of the outdoor area. The genotype D grazed much less, based on visual evaluations of green biomass coverage of the outdoor area (by photos) than the genotypes A and B, which showed very high activity in foraging. The hens of genotype C were assessed to be between A/B and D in terms of foraging activity. At mid-summer, i.e., hens age 30 week, the vegetation coverage in the outdoor units could be negatively correlated with the egg yolk color represented by redness a* values. This meant that genotypes A and B had foraged the most, i.e., the outdoor area was mainly bare soil, and laid eggs with more red egg yolks, and vice versa for genotype D (Figure 4). At the egg samplings from week 38 and onwards, the yolk color values remained at a steady but lower level, which is speculated to be due to the fact that some vegetation still did grow, but was eaten relatively fast.

There may be an interaction effect of the genotypes used as they show different yolk colors and therefore may differ in their efficiency in depositing carotenoids in the egg yolk. Other studies have reported a possible interaction between genotype and environment when it comes to incorporating dietary fatty acids into the egg yolk [28].

### 4.5. Albumen Quality

The most significant egg albumen quality attribute is its dry matter content. It provides an indication of the albumen protein synthesis as protein comprises out ~85–90% of the dry matter [12,42]. The content of protein is important for the egg as food, as the protein is responsible for the functional properties of, for example, gel texture in boiled eggs and foaming properties in whipped foods [12,43]. Hence, the high DM content in eggs of genotype A is regarded a valuable quality characteristic in food applications, and albumen DM in both A and C genotypes exceeded that of the egg albumen of the egg layer genotype D. The observed negative effect of hen age on the albumen content of dry matter is well-known. It can be the result of a lack of essential amino acids, which was not expected to be the case in the present experiment. The literature on dual-purpose genotypes and albumen protein or albumen DM content is limited. One recent study reports no significant difference in albumen protein between a commercial egg layer and four local Portuguese genotypes [44]. Few reports are available on albumen pH and albumen height as quality attributes, which indirectly also provides indications on shell quality as both are related to the CO_2_ exchange and water evaporation through the shell, where dual-purpose and broiler genotypes often are inferior to egg layer genotypes [45,46]. Nevertheless, the genetics of egg producing poultry have a significant impact on the egg content and composition [47].

### 4.6. Egg and Meat from Dual-Purpose Poultry

The egg production and egg quality of dual-purpose hens is from an overall point of view only half the story. The meat production of the male side of dual-purpose genotypes is likewise important to deliver on yield and meat quality criteria.

By nature, the meat production traits of egg layer genotypes are expected to be lower, and from broiler types to be higher, than that of dual-purpose genotypes, due to large differences in growth rate, feed efficiency, and optimal age of slaughter that impacts the meat quality, which has been demonstrated in a range of recent studies [28,48,49,50]. The meat quality based on protein, lipids, cholesterol, and fatty acids in hens of dual-purpose varies in comparison to egg layer genotypes, but the results are also affected by the production environment and its interaction with genotype [28]. Other researchers find that the dual-purpose meat quality is competitive with the meat quality of slow-growing broilers regarding tenderness and water holding capacity [50]. 

Comparing meat quality of dual-purpose genotypes with both classical broilers (2 genotypes) and an egg layer (Lohmann Brown Plus), it can be concluded that the meat quality, measured as tenderness and water holding properties, was most favorable in the classical intensive broilers, whereas meat quality did not vary between the other types; i.e., dual-purpose genotypes and the egg layer [51]. 

## 5. Conclusions

In summary, it is not straightforward to draw a simple recommendation on the implementation of dual-purpose genotypes and a range of criteria must be met also on the meat quality side when changing from egg layer genotypes into dual-purpose genotypes for egg production. Here we addressed the egg quality, which is required to be high for both a feasible economic production of the farmer, and for the consumer willingness to pay a higher egg-price as production costs will be higher than for the egg production with egg layer genotypes. To avoid killing of day-old male layer chickens, more research is needed on e.g., feather pecking behavior [52], meat quality [28], and dietary needs for production [27,48] to be able to implement dual-purpose genotypes in the poultry production of tomorrow. 

Based on egg quality parameters of shell, albumen, and yolk of four different poultry genotypes, it can be concluded that laid eggs of genotype A were of comparable quality levels as those of the commercial egg layer genotype. The two other genotypes (B) and (C) were in general either inferior in quality parameters or not different to the two highest egg quality levels of (D) and (A). 

Eggs of genotype A had higher egg weight, higher shell-to-egg compression, more red-yellow yolk color, larger yolks by mass and percentage, higher albumen DM, and relatively lower albumen mass in comparison to the commercial egg layer (D). On the other hand, higher frequency of blood- and meat-spots, lower shell strength, and lower shell percentage were observed. These differences are significant but could be regarded as small, and can realistically pave the way for a commercial production of dual-purpose eggs from genotype (A). Among several other parameters to take into consideration, as mentioned earlier, before application of dual-purpose genotypes, it is also of interest to explore how the males of dual-purpose genotypes perform in meat production and deliver on meat quality attributes.

## Figures and Tables

**Table 1:** Effect of hen genotype A, B, C, and D, age, and their interaction on LS-means of egg characteristics between the 21st and 54th week of age, *n* = 180 for genotype A and 270 for genotypes B, C and D. For blood spot and meat spot frequency, data are calculated as mean/unit/age, *n* = 12 for genotype A and 18 for genotypes B, C and D.

Genotype (G)	A	B	C	D	Effect of			SEM
					G	Age	G * Age	
Egg weight, g	60.45 ^b^	62.14 ^a^	60.51 ^b^	59.53 ^c^	***	***	***	0.240
Egg diameter, mm	42.38 ^b^	42.84 ^a^	42.96 ^a^	42.58 ^b^	***	***	***	0.066
Blood spot%	3.9 ^b,c^	25.2 ^a^	8.2 ^b^	0.7 ^c^	***	NS	NS	2.003
Meat spot%	13.3 ^a^	15.9 ^a^	16.3 ^a^	5.2 ^b^	**	NS	NS	2.038

a–c values of same parameter with different letter superscript are significantly different at * (*p* < 0.05), ** (*p* < 0.01) or *** (*p* < 0.001). NS = non-significant. SEM = standard error of mean.

**Table 2:** Effect of hen genotype, A, B, C, and D, age, and their interaction on LS-means of egg shell parameters between the 21st and 54th week of age, *n* = 180 for genotype A and 270 for genotypes B, C and D.

Genotype (G)	A	B	C	D	Effect of			SEM
					G	Age	G * Age	
Shell strength at fracture, N	47.2 ^b^	38.3 ^d^	42.7 ^c^	50.6 ^a^	***	***	NS	0.423
Shell-to-egg compression,%	1.014 ^a^	0.951 ^c^	0.953 ^c^	0.984 ^b^	***	***	NS	0.007
Shell stiffness, N/mm ^NB^	75.8 ^b^	73.4 ^b^	75.4 ^b^	82.0 ^a^	***	***	NS	0.928
Shell thickness, mm	0.438 ^b^	0.403 ^d^	0.415 ^c^	0.444 ^a^	***	***	*	0.002
Shell weight, g	6.0 ^a^	5.5 ^c^	5.7 ^b^	6.0 ^a^	***	***	**	0.031
Shell, % (*w*/*w*)	9.9 ^b^	8.9 ^d^	9.4 ^c^	10.1 ^a^	***	***	*	0.043

a–d values of same parameter with different letter superscript are significantly different at * (*p* < 0.05), ** (*p* < 0.01) or *** (*p* < 0.001). NS = non-significant. NB: data analysis was performed on log-transformed data. SEM = standard error of mean.

**Table 3:** Effect of hen genotype A, B, C, and D, age, and their interaction on LS-means of egg yolk parameters between the 21st and 54th week of age, *n* = 180 for genotype A and 270 for genotypes B, C and D.

Genotype (G)	A	B	C	D	Effect of			SEM
					G	Age	G * Age	
Yolk colour lightness, L*	62.5 ^a,b^	62.3 ^b^	62.1 ^b^	63.3 ^a^	***	**	*	0.297
Yolk colour redness, a*	0.409 ^a^	0.271 ^a^	−0.339 ^b^	−0.605 ^b^	***	***	***	0.169
Yolk colour yellowness, b*	57.3 ^a^	55.5 ^b^	53.6 ^c^	53.5 ^c^	***	***	**	0.360
Yolk weight, g	16.3 ^a^	15.9 ^b^	15.4 ^c^	15.0 ^d^	***	***	***	0.0904
Yolk,% (*w*/*w*)	26.7 ^a^	25.4 ^b^	25.3 ^b^	24.9 ^b^	***	***	NS	0.182

a–d values of same parameter with different letter superscript are significantly different at * (*p* < 0.05), ** (*p* < 0.01) or *** (*p* < 0.001). NS = non-significant. SEM = standard error of mean.

**Table 4:** Effect of hen genotype A, B, C, and D, age, and their interaction on LS-means of egg albumen parameters between the 21st and 54th week of age, *n* = 180 for genotype A and 270 for genotypes B, C and D.

Genotype (G)	A	B	C	D	Effect of			SEM
					G	Age	G * Age	
Albumen pH	9.41	9.35	9.36	9.36	NS	***	NS	0.019
Albumen DM,%	13.58 ^a^	13.13 ^c^	13.34 ^b^	13.08 ^c^	***	***	*	0.042
Albumen, % (*w*/*w*)	63.4 ^c^	65.7 ^a^	65.3 ^a,b^	65.0 ^b^	***	***	NS	0.199

a–c values of same parameter with different letter superscript are significantly different at * (*p* < 0.05) or *** (*p* < 0.001). NS = non-significant. SEM = standard error of mean. DM = dry matter.

## Data Availability

Not applicable.

## Supplementary Materials

The following are available online at https://www.mdpi.com/2304-8158/10/4/897/s1, Table S1: Raw data of egg quality parameters with ATOL (Animal Trait Ontology for Livestock) descriptors, for hen.
