# Correction: Hammershøj et al. Dual-Purpose Poultry in Organic Egg Production and Effects on Egg Quality Parameters. *Foods* 2021, *10*, 897

**DOI:** 10.3390/foods11030311

**Published:** 2022-01-24

**Authors:** Marianne Hammershøj, Gitte Hald Kristiansen, Sanna Steenfeldt

**Affiliations:** 1 Department of Food Science, Aarhus University, Agro Food Park 48, DK-8200 Aarhus, Denmark; ghk@food.au.dk; 2 Department of Animal Science, Aarhus University, Blichers Alle 20, DK-8830 Tjele, Denmark; sanna.steenfeldt@anis.au.dk

The authors would like to make the following correction to the published paper [1].

## Text Correction

There was an error in the original publication. After publishing the results, a mistake in the chick delivery for genotype A, was discovered. This means that fewer chickens than expected were available for genotype A, resulting in only two replicate units for genotype A instead of three replicates that was planned. The results have been corrected by calculating the LS-Means of all data by two replicate units of genotype A, and by three replicate units of genotypes B, C and D, respectively. 

A correction has been made to *Abstract, lines 7–11*:

Two dual-purpose genotypes with divergent characteristics were evaluated: genotype A represented an experimental crossbreed based on a broiler type male and an egg layer female, and genotype C was a crossbreed of a layer type. These were compared to a rustic genotype B and a control genotype D, which was an egg layer. Eggs were collected six times during the period of 21–54 weeks of hen age, i.e., a total of 990 shell eggs were analyzed. 

A correction has been made to *Section 2.1. Materials, first paragraph, lines 1–7*:

Eggs were produced from four different genotypes in the study, including two dual-purpose genotypes (A, C), a rustic breed (B) and a commercial egg layer (D). The dual-purpose genotypes with divergent characteristics, and a rustic genotype were selected by the French Poultry and Aquaculture Breeders Technical Center (SYSAAF) in cooperation with two breeding companies. Genotype A represented an experimental cross breed based on a broiler type male and an egg layer female laying brown-shelled eggs, where genotype C was a cross breed of a layer type laying brown-shelled eggs. Genotype B represented a genotype that has not been selected for any specific traits and included to compare with the dual-purpose genotypes orientated more on meat or eggs production. Finally, genotype D was a control egg layer breed laying white-shelled eggs (purchased at a local pullet breeder). However, due to a mistake in the chick delivery for genotype A, fewer chickens than expected were available for genotype A, which resulted in only two replicate units for genotype A instead of three replicates that was planned. For genotype B, C and D, there were three replicate units.

A correction has been made to *Section 2.1. Materials, fourth paragraph, lines 1–3*:

Six times, at the hen ages of 21, 25, 30, 38, 46, and 54 week, 15 eggs were collected from each of the 11 outdoor units with mobile houses, representing the four genotypes in two (A) and three replicates (B, C and D), i.e., a total of 990 shell eggs were individually analyzed.

A correction has been made to *Section 2.1. Materials, fifth paragraph, lines 1–4*:

The eggs were stored at 22 °C until analysis. On day 1 after egg collection, the 165 eggs were marked and individually weighed; any visually cracked eggs were removed. For practical reasons in order to overcome 165 egg samples, the parameter analysis was distributed over several days. 

A correction has been made to *Section 2.2.4. Data Analysis, first paragraph, lines 1–4*:

A two-way analysis of variance (ANOVA) with class variables of four genotypes (A, …, D) and 6 hen ages (21, …, 54 weeks) with 2–3 replicate outdoor units (1, 2, 3) of 15 eggs analyzed per unit was included as model with interactions between age and genotype. 

A correction has been made to *Section 2.2.4. Data Analysis , fourth paragraph, lines 4–5*:

Least Squared Means (LS-means) were considered significantly different at minimum 95-% level (*p* ≤ 0.05). 

A correction has been made to *Section 2.2.4. Data Analysis, after fifth paragraph*:

Individual data are available in Supplementary Table S1, where traits are whenever possible presented in reference to ontology ATOL: https://www.atol-ontology.com/en/atol-2/ (accessed on 2 December 2021)

A correction has been made to *Section 3. Results, second paragraph, lines 2–3*:

The egg weight from genotype A was no different from those of genotype C, but both laid egg of significantly higher weight than eggs of genotype D. 

A correction has been made to *Section 3. Results, third paragraph, lines 1–3*:

Generally, the egg diameter increases as eggs get larger (Table 1, Figure 1B), and eggs from genotype B and C had greatest values (*p* < 0.001) for diameter compared to egg diameters of genotype A and D. 

A correction has been made to *Section 3. Results, fifth paragraph, lines 1–7*:

All the shell quality parameters were significantly affected by hen age (*p* < 0.001) and genotype (*p* < 0.001), while significant interactions between age and genotype were found only for the shell thickness, shell weight and shell percentage (*p* < 0.01–0.05) (Table 2, Figure 2). Overall, the egg layer genotype D had the significantly highest values of all shell parameters, apart from the shell-to-egg compression, where genotype A laid eggs that had a higher value (Table 2), while the genotypes B and C produced eggs with inferior shell quality parameters. 

A correction has been made to *Section 3. Results, seventh paragraph, line 3*:

The egg layer genotype D had significantly fewer red, and fewer yellow egg yolks …

A correction has been made to *Section 3. Results, seventh paragraph, lines 14–16*:

This genotype difference persisted throughout the total experimental period, and became more pronounced as hen age increased (Figure 3D), with a peak at 54 week in egg yolk weight from 17.2 g of genotype D eggs to 19.5 g of genotype A eggs. 

A correction has been made to *Section 3. Results, eight paragraph, lines 3–5*:

The values of yolk color redness at 30 week of hen age was shown to correlate negatively (r = −0.889) with the visual grading score of vegetation coverage in the units (Figure 4B). 

A correction has been made to *Section 3. Results, tenth paragraph, lines 1–6*:

Based on the LS-means of egg weight, albumen-% and albumen dry matter relative weight of the eggs (Table 4, Figure 5) from the four genotypes, the produced mass of albumen dry matter per egg was calculated on average to be 5.06 g/egg for egg layer genotype D, 5.20–5.27 g/egg for genotypes C and A, while genotype B eggs contained the overall highest albumen dry matter of 5.36 g/egg, mainly caused by increased egg weight and albumen weight. 

A correction has been made to *Section 4.1. Egg Weight, Proportions, and Inclusions, first paragraph, lines 8–11*:

The egg weight increase in genotype D from week 24 to week 54 was 5.4 g corresponding to a 9% increase, a value that for the genotypes A–C ranged from 7.7–9.8 g, which corresponded to 13–17% increase, with genotype B having the highest values. 

A correction has been made to *Section 4.1. Egg Weight, Proportions, and Inclusions, third paragraph, lines 6–7*:

Even though genotype A laid larger eggs than genotype D, the yolk weight was also higher and also increased with age at a higher rate. 

A correction has been made to *Section 4.1. Egg Weight, Proportions, and Inclusions, fifth paragraph, lines 5–8*:

Their level was very low in the eggs from layer genotype D (5.2 and 0.7%, respectively), while in the dual-purpose genotypes A–C higher meat (13–16%) and blood (4–25%) spot frequencies were observed and have been ascribed to a lack of genetic selection in breeding to avoid them. 

A correction has been made to *Section 4.2. Overall Egg Quality and Hen Age, second paragraph, lines 2–5*:

The genotype A generally produced eggs of comparable shell quality, higher yolk mass, more red-yellow colored yolk, higher albumen DM, and higher egg weight with greater inclusion of blood spots and meat spots compared to the layer genotype D. 

A correction has been made to *Section 4.3. Shell Quality, first paragraph, lines 6–8*:

… however, the genotype A seemed to maintain a reasonable shell stiffness during the experimental period, and the shell thickness of the genotype A was at a numerical high level although significantly different from genotype D.

A correction has been made to *Section 4.4. Yolk Quality, first paragraph, lines 3–5*:

… which is similar to the findings with the egg layer genotype D yolk-% being 24.9% and the dual-purpose genotypes A–C yolk-% of 25.8% on average, as the yolks of genotype A in general were darkest. 

A correction has been made to *Acknowledgements:*

The study has been carried out in cooperation with partners in the Horizon 2020 PPILOW project. We thank the partners for valuable support and contribution in relation to the selection of the specific genotypes, discussions of experimental design and relevant data collection, identification of ontologies for egg traits obtained in the study: SYSAAF (French Poultry and Aquaculture Breeders Technical Center) and the breeding companies Novogen and Hendrix-genetics. The Institutes INRAE (National Research Institute for Agriculture, Food and Environment), Université de Tours, BOA and INRAE EASM, France; ITAB (Institute of Organic Agriculture and Food), France, and Thuenen Institute of Organic Farming, Germany. The authors thank Anne Louise Frydendahl Hellwing, Dept. of Animal Science, Aarhus University for photos of outdoor units in Figure 4.

## Error in Figure/Table and Figure/Table Legend

In the original article, there was a mistake in Table 1 as published and the legend for Table 1. The correct legend and corrected Table 1 appear below. 

**Table 1:** Effect of hen genotype A, B, C, and D, age, and their interaction on LS-means of egg characteristics between the 21st and 54th week of age, *n* = 180 for genotype A and 270 for genotypes B, C and D. For blood spot and meat spot frequency, data are calculated as mean/unit/age, *n* = 12 for genotype A and 18 for genotypes B, C and D.

Genotype (G)	A	B	C	D	Effect of			SEM
					G	Age	G * Age	
Egg weight, g	60.45 ^b^	62.14 ^a^	60.51 ^b^	59.53 ^c^	***	***	***	0.240
Egg diameter, mm	42.38 ^b^	42.84 ^a^	42.96 ^a^	42.58 ^b^	***	***	***	0.066
Blood spot%	3.9 ^b,c^	25.2 ^a^	8.2 ^b^	0.7 ^c^	***	NS	NS	2.003
Meat spot%	13.3 ^a^	15.9 ^a^	16.3 ^a^	5.2 ^b^	**	NS	NS	2.038

a–c values of same parameter with different letter superscript are significantly different at * (*p* < 0.05), ** (*p* < 0.01) or *** (*p* < 0.001). NS = non-significant. SEM = standard error of mean.

In the original article, there was a mistake in Table 2 as published and the legend for Table 2. The correct legend and corrected Table 2 appear below. 

**Table 2:** Effect of hen genotype, A, B, C, and D, age, and their interaction on LS-means of egg shell parameters between the 21st and 54th week of age, *n* = 180 for genotype A and 270 for genotypes B, C and D.

Genotype (G)	A	B	C	D	Effect of			SEM
					G	Age	G * Age	
Shell strength at fracture, N	47.2 ^b^	38.3 ^d^	42.7 ^c^	50.6 ^a^	***	***	NS	0.423
Shell-to-egg compression,%	1.014 ^a^	0.951 ^c^	0.953 ^c^	0.984 ^b^	***	***	NS	0.007
Shell stiffness, N/mm ^NB^	75.8 ^b^	73.4 ^b^	75.4 ^b^	82.0 ^a^	***	***	NS	0.928
Shell thickness, mm	0.438 ^b^	0.403 ^d^	0.415 ^c^	0.444 ^a^	***	***	*	0.002
Shell weight, g	6.0 ^a^	5.5 ^c^	5.7 ^b^	6.0 ^a^	***	***	**	0.031
Shell, % (*w*/*w*)	9.9 ^b^	8.9 ^d^	9.4 ^c^	10.1 ^a^	***	***	*	0.043

a–d values of same parameter with different letter superscript are significantly different at * (*p* < 0.05), ** (*p* < 0.01) or *** (*p* < 0.001). NS = non-significant. NB: data analysis was performed on log-transformed data. SEM = standard error of mean.

In the original article, there was a mistake in Table 3 as published and the legend for Table 3**.** The correct legend and corrected Table 3 appear below. 

**Table 3:** Effect of hen genotype A, B, C, and D, age, and their interaction on LS-means of egg yolk parameters between the 21st and 54th week of age, *n* = 180 for genotype A and 270 for genotypes B, C and D.

Genotype (G)	A	B	C	D	Effect of			SEM
					G	Age	G * Age	
Yolk colour lightness, L*	62.5 ^a,b^	62.3 ^b^	62.1 ^b^	63.3 ^a^	***	**	*	0.297
Yolk colour redness, a*	0.409 ^a^	0.271 ^a^	−0.339 ^b^	−0.605 ^b^	***	***	***	0.169
Yolk colour yellowness, b*	57.3 ^a^	55.5 ^b^	53.6 ^c^	53.5 ^c^	***	***	**	0.360
Yolk weight, g	16.3 ^a^	15.9 ^b^	15.4 ^c^	15.0 ^d^	***	***	***	0.0904
Yolk,% (*w*/*w*)	26.7 ^a^	25.4 ^b^	25.3 ^b^	24.9 ^b^	***	***	NS	0.182

a–d values of same parameter with different letter superscript are significantly different at * (*p* < 0.05), ** (*p* < 0.01) or *** (*p* < 0.001). NS = non-significant. SEM = standard error of mean.

In the original article, there was a mistake in Table 4 as published and the legend for Table 4. The correct legend and corrected Table 4 appear below. 

**Table 4:** Effect of hen genotype A, B, C, and D, age, and their interaction on LS-means of egg albumen parameters between the 21st and 54th week of age, *n* = 180 for genotype A and 270 for genotypes B, C and D.

Genotype (G)	A	B	C	D	Effect of			SEM
					G	Age	G * Age	
Albumen pH	9.41	9.35	9.36	9.36	NS	***	NS	0.019
Albumen DM,%	13.58 ^a^	13.13 ^c^	13.34 ^b^	13.08 ^c^	***	***	*	0.042
Albumen, % (*w*/*w*)	63.4 ^c^	65.7 ^a^	65.3 ^a,b^	65.0 ^b^	***	***	NS	0.199

a–c values of same parameter with different letter superscript are significantly different at * (*p* < 0.05) or *** (*p* < 0.001). NS = non-significant. SEM = standard error of mean. DM = dry matter.

In the original article, there was a mistake in Figure 1 as published and the legend for Figure 1. The correct legend and corrected Figure 1 appear below.

**Figure 1:**
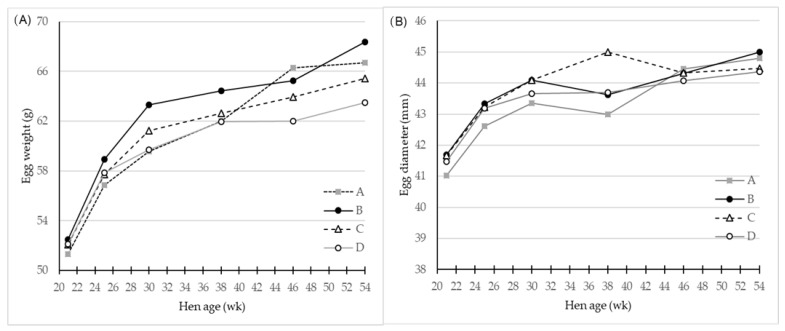
LS-means of (**A**) Egg weight (g) and (**B**) egg diameter (mm) for eggs of four different hen genotypes A, B, C, and D as differentiated by hen age (week), *n* = 30 for genotype A and 45 for genotypes B, C and D. There was a significant (*p* < 0.001) interaction effect of genotype and hen age on both parameters.

In the original article, there was a mistake in Figure 2 as published and the legend for Figure 2. The correct legend and corrected Figure 2 appear below.

**Figure 2:**
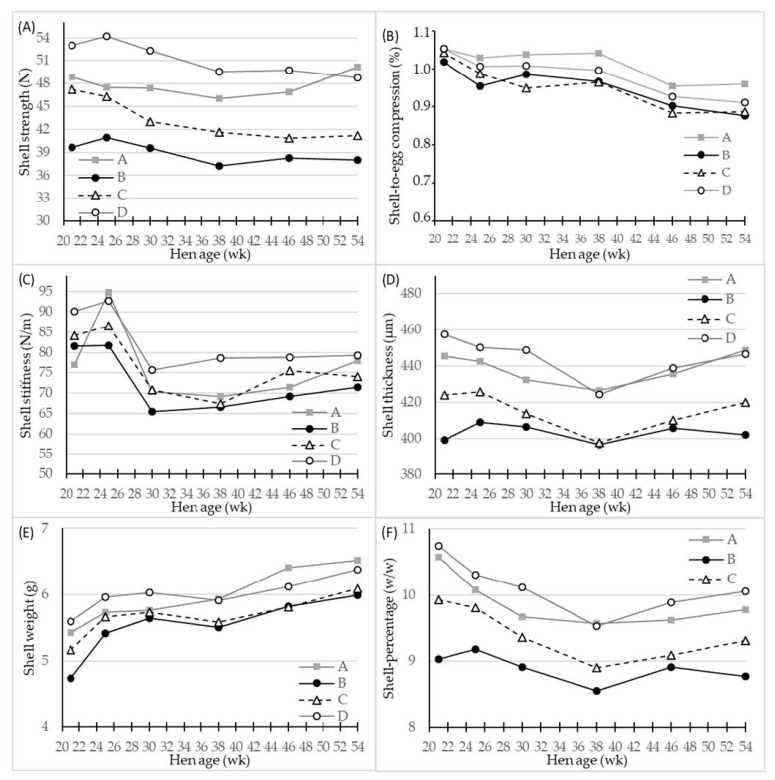
LS-means of shell parameters of four different hen genotypes (**A**–**D**) as differentiated by hen age (week). (**A**) shell strength (N) at compression, (**B**) shell-to-egg-compression (%), (**C**) shell stiffness (N/m), (**D**) shell thickness (µm) at equator, (**E**) shell weight (g) after air drying, and (**F**) shell percentage (*w*/*w*), *n* = 30 for genotype A and 45 for genotypes B, C and D.

In the original article, there was a mistake in Figure 3 as published and the legend for Figure 3. The correct legend and corrected Figure 3 appear below.

**Figure 3:**
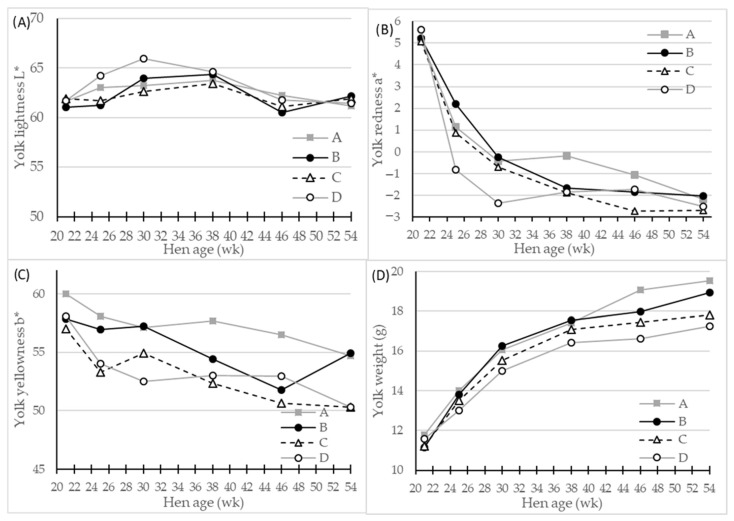
LS-means of egg yolk color parameters and egg mass of four different hen genotypes (**A**–**D**) as differentiated by hen age (week). Panels showing (**A**) L* (lightness), 0 = black, 100 = white, (**B**) a* (redness), −100 = green, 100 = red, (**C**) b* (yellowness), −100 = blue, 100 = yellow, and (**D**) egg yolk weight, *n* = 30 for genotype A and 45 for genotypes A, B and C, significant interactions of hen age * genotype (*p* < 0.01) for all parameters.

In the original article, there was a mistake in Figure 4 as published and the legend for Figure 4. The correct legend and corrected Figure 4 appear below.

**Figure 4:**
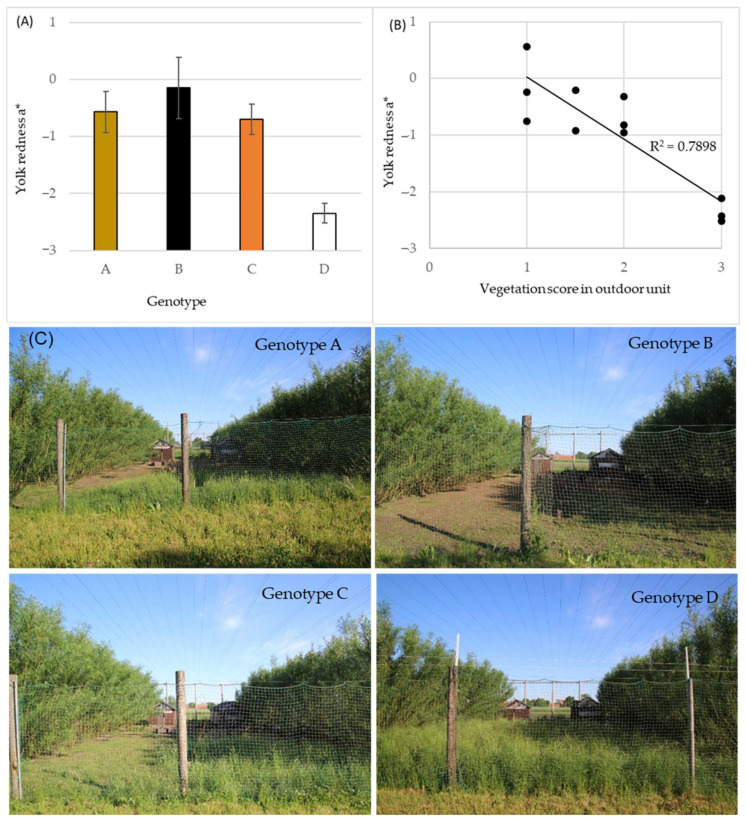
(**A**) LS-mean egg yolk color redness a* at hen age 30 week for four hen genotypes A–D, *n* = 30 for genotype A and 45 for genotypes B, C and D, vertical bars indicate standard deviations. (**B**) Correlation of scores for visual evaluation of vegetation coverage in outdoor units of organic poultry 3 weeks prior to egg collection and yolk color redness a* of panel A data. Each data point represents one pen and 15 egg yolk colors. Score 1 = mainly bare soil, 2 = partly covered with vegetation, 3 = full covered with vegetation. (**C**) Representative photos of outdoor units for hen genotypes A–D at time for vegetation scoring.

In the original article, there was a mistake in Figure 5 as published and the legend for Figure 5. The correct legend and corrected Figure 5 appear below.

**Figure 5:**
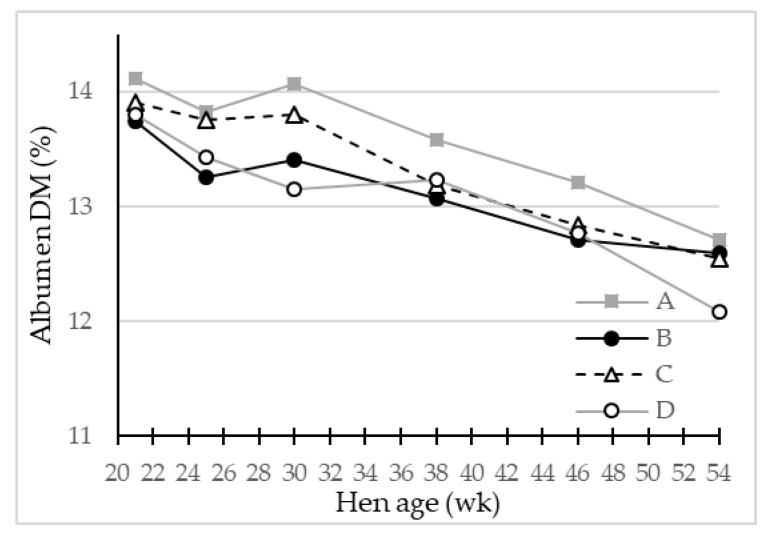
LS-means of egg albumen dry matter (DM) (%) in eggs from four different hen genotypes (A–D) as function of hen age (week) (*p* < 0.05), *n* = 30 for genotype A and 45 for genotypes B, C and D.

The authors apologize for any inconvenience caused and state that the scientific conclusions are unaffected. The original article has been updated.
