# Supplementary material for: Dual-Purpose Poultry in Organic Egg Production and Effects on Egg Quality Parameters
Source: Foods. 2021 Apr 19;10(4):897. doi: 10.3390/foods10040897 (PMC8072786; doi:10.3390/foods10040897)
Supplement: Supplementary file 1 [file foods-10-00897-s001.zip › foods-1515398-supplementary.pdf]

Supplementary Table S1. Raw data of egg quality parameters with ATOL (Animal Trait Ontology for Livestock) descriptors, for hen.

				ATOL_ 0001880	ATOL_ 0002297	ATOL_ 0002119	ATOL_ 0002126	ATOL_ 0002122	ATOL_ 0002074	ATOL_ 0002073	ATOL_ 0002075	ATOL_ 0005600	ATOL_ 0005600	ATOL_ 0001883	ATOL_ 0005587	ATOL_ 0001938	ATOL_ 1881	ATOL_ 0002121	ATOL_ 1881	ATOL_ 0001883	ATOL_ 0001882	ATOL_ 0002125
**Hen age**	**Pen**	**Genotype**	**Replicate**	**Egg weight**	**Egg diameter**	**Shell strength**	**Shell fracture**	**Shell modulus**	**Yolk lightness**	**Yolk redness**	**Yolk yellow**	**Blood spot**	**Meat spot**	**Yolk weight**	**Albumen pH**	**Albumen DM**	**Shell weight**	**Shell thickness**	**Shell**	**Yolk**	**Albumen**	**Shell-to-egg-compression**
wk	no.			g	mm	N	mm	N/mm	L*	a*	b*	1=present	1=present	g	-	% (w/w)	g	mm	% (w/w)	% (w/w)	% (w/w)	%
21	1	C	1	43,659	39,967499	36,627537	0,377499	67,562325	62,6	2,73	53,02			11,341	9,41	13,819934	3,911	0,3691667	8,9580613	25,976316	65,065622	0,944514942
21	1	C	2	51,442	41,712502	55,543613	0,432484	108,8504	63,2	2,72	53,88			9,903	9,37	13,778934	5,242	0,4316667	10,190117	19,250807	70,559076	1,036821047
21	1	C	3	51,738	41,465	39,930344	0,444984	97,590027	64,46	4,64	57,01			10,584	9,39	14,178825	4,678	0,3875	9,0417102	20,456918	70,501372	1,073155673
21	1	C	4	50,807	41,712498	45,78532	0,412498	108,8504	60,71	4,63	55,9		1	11,849	9,35	14,136825	5,124	0,425	10,085224	23,32159	66,593186	0,988907449
21	1	C	5	55,614	43,252499	44,133915	0,409985	105,08691	62,85	9,36	63,9			12,135	9,47	14,198303	5,429	0,4216667	9,7619304	21,820045	68,418024	0,947887427
21	1	C	6	51,352	42,25	54,792976	0,449997	101,3435	60,22	7,18	54,1		1	10,805	9,43	14,156303	5,339	0,4375	10,396869	21,04105	68,562081	1,065081657
21	1	C	7	55,072	42,764999	56,894764	0,399982	120,0879	62,81	5,84	54,91			11,983	9,46	13,725293	6,004	0,465	10,902092	21,758788	67,33912	0,935302255
21	1	C	8	55,096	41,919998	37,077919	0,397488	90,083107	63,45	3,45	60,61		1	12,661	9,42	13,683293	5,216	0,3958333	9,467112	22,97989	67,552998	0,948206152
21	1	C	9	44,137	39,802498	51,640297	0,449993	82,584053	62,58	4,84	55,95	1		10,69	9,5	13,318872	4,477	0,39	10,143417	24,220042	65,636541	1,13056472
21	1	C	10	47,574	41,195004	47,73698	0,37999	97,590012	64,56	1,01	57,32			10,751	9,46	13,276872	5,003	0,4391667	10,516248	22,598478	66,885273	0,92241768
21	1	C	11	55,693	42,294998	41,882004	0,334991	123,85246	65,92	3,37	57,98		1	12,142	9,44	13,780947	5,576	0,4391667	10,01203	21,801663	68,186307	0,792034557
21	1	C	12	65,096	45,355	44,884556	0,414997	75,06208	63,9	6,81	58	1		12,462	9,4	13,738947	5,804	0,4116667	8,9160624	19,144033	71,939904	0,914997244
21	1	C	13	56,322	43,149998	67,253563	0,462486	90,074501	59,28	8,3	53,86			11,014	9,46	14,060735	6,474	0,4975	11,49462	19,555414	68,949966	1,071810015
21	1	C	14	51,87	41,489998	38,278938	0,422493	86,329628	66,43	3,69	64,49		1	10,262	9,42	14,018735	4,447	0,4691667	8,5733565	19,784076	71,642568	1,018300844
21	1	C	15	53,103	42,25	58,546165	0,437485	105,09694	64,21	7,56	59,17			11,831	9,49		5,707	0,4608333	10,747039	22,279344	66,973617	1,035467456
21	2	D	1	51,748	41,095001	45,78532	0,4175	82,568283	65,22	5,99	55,69		1	10,823	9,43	14,227401	5,372	0,4316667	10,381078	20,914818	68,704105	1,015938654
21	2	D	2	49,046	41,1325	52,691189	0,464985	90,083107	66,13	4,82	59,56			11,092	9,39	14,185401	5,007	0,4275	10,208784	22,615504	67,175713	1,130456452
21	2	D	3	67,801	44,212498	39,780216	0,409988	52,54847	57,37	9,46	65,845			22,409	9,44	13,623585	6,15	0,3858333	9,0706627			0,927312454
21	2	D	4	51,433	40,677502	48,037235	0,429989	63,808853	56,81	4,87	57,87			11,381	9,4	13,581585	5,152	0,4116667	10,016915	22,127817	67,855268	1,057068352
21	2	D	5	51,508	41,599998	55,843868	0,469997	97,580727	68,93	5,02	60,65			11,146	9,45	14,567662	5,626	0,4366667	10,922575	21,639357	67,438068	1,129800535
21	2	D	6	49,417	40,862499	49,388382	0,414997	75,076401	66,45	6,08	62,13			11,656	9,41	14,525662	5,457	0,4508333	11,042759	23,587025	65,370217	1,015593784
21	2	D	7	49,583	41,087502	57,044891	0,409996	101,3435	64,57	6,57	58,65			10,905		13,518192	5,623	0,46	11,34058	21,993425	66,665994	0,997860615
21	2	D	8	65,352	43,555	47,887108	0,474995	86,329628	51,65	6,42	64,93					13,560192	6,125	0,4383333	9,3723222			1,090563655
21	2	D	9	50,337	41,005001	60,497826	0,454998	75,076401	68,64	5,14	60,61			10,19	9,39	14,216362	5,78	0,4758333	11,482607	20,243558	68,273834	1,109615873
21	2	D	10	57,602	43,105	52,991444	0,432491	90,074501	65,05	10,03	59,81			12,792	9,35	14,174362	6,097	0,4608333	10,584702	22,207562	67,207736	1,003343
21	2	D	11	52,363	41,014999	52,090679	0,419998	90,083099	55,59	7,44	53,16			10,825	9,42	13,276153	5,838	0,4566667	11,149094	20,672994	68,177912	1,024010753
21	2	D	12	63,952	43,612499	47,887108	0,422489	82,568291	55,965	11,765	59,23			16,964	9,38	13,234153	6,534	0,4625	10,217038			0,968733757
21	2	D	13	48,638	40,532501	54,342594	0,37249	97,590027	65,64	4,62	59,22			10,485	9,47	14,000216	5,273	0,46	10,841317	21,557219	67,601464	0,918990911
21	2	D	14	49,077	41,362499	46,235703	0,422489	90,074509	62,53	7,85	56,09		1	10,123	9,43	13,958216	5,023	0,4625	10,234937	20,62677	69,138293	1,021430064
21	2	D	15	51,67	40,959999	49,988892	0,437489	90,091675	60,66	5,76	59,92			11,131	9,36		5,245	0,4791667	10,150958	21,542481	68,306561	1,068088405
21	3	B	1	63,961	43,940002	48,487617	0,437492	67,562325	60,37	4,77	57,98		1	12,868	9,44	13,908043	6,116	0,4425	9,5620769	20,11851	70,319413	0,99565767
21	3	B	2	52,111	42,762501	45,184811	0,439987	86,304947	68,33	0,79	56,29			10,87	9,4	13,866043	4,837	0,4008333	9,2821093	20,85932	69,858571	1,028908482
21	3	B	3	52,86	42,735001	40,530853	0,450001	78,830238	56,75	2,87	54,38	1		10,525	9,37	14,320803	4,348	0,3675	8,2255013	19,911086	71,863413	1,053003368
21	3	B	4	52,094	41,959999	46,535957	0,442486	101,35313								14,278803	4,825	0,405	9,2621031			1,054542446
21	3	B	5	48,411	41,102501	44,734428	0,40749	105,09694	59,03	5,31	60,1	1		8,992	9,45		4,905	0,42	10,131995	18,574291	71,293714	0,991399526
21	3	B	6	51,333	41,772499	42,032131	0,459991	90,065918	62,43	0,43	59,89	1		13,224	9,47	13,374674	4,554	0,3991667	8,8714862	25,761206	65,367308	1,101181426
21	3	B	7	57,849	42,887501	47,586853	0,457493	75,0084	68	1,87	66,6			11,601	9,46	13,364674	5,892	0,4425	10,185137	20,053934	69,760929	1,066728043
21	3	B	8	56,701	42,802498	51,49017	0,447495	93,836563	62,84	3,5	59,56			12,615	9,48	13,893452	5,858	0,445	10,331387	22,248285	67,420328	1,045488046
21	3	B	9	57,177	42,684998	36,327282	0,432487	82,584038	67,46	5,4	65,67			12,347	9,44	13,851452	5,151	0,405	9,0088672	21,594347	69,396785	1,013206092
21	3	B	10	50,25	41,737499	39,479961	0,407494	90,065926	61,95	3,6	54,41			12,696	9,51	13,848993	4,671	0,395	9,2955224	25,265672	65,438806	0,976325869
21	3	B	11	54,702	41,329998	31,222942	0,372486	78,807678	59,18	5,14	57,27			13,178	9,47	13,806993	4,638	0,3783333	8,4786662	24,090527	67,430807	0,901248531
21	3	B	12	50,239	40,032501	37,528301	0,409992	56,301926	59,52	6,05	56,48	1		11,314	9,45	13,901446	4,604	0,3941667	9,1641951	22,520353	68,315452	1,024147854
21	3	B	13	53,161	41,889999	49,088127	0,484997	105,08692	67,38	1,2	57,98	1		11,68	9,45	13,859446	5,33	0,4191667	10,026147	21,970994	68,002859	1,157787089
21	3	B	14	49,651	40,137501	41,731876	0,389992	67,562317	62,03	5,56	58,99			11,327	9,4	13,102005	4,583	0,4091667	9,2304284	22,813236	67,956335	0,971639963
21	3	B	15	47,858	39,915001	33,624985	0,374992	63,797607	62,97	4,95	55,57			11,118	9,42	13,122005	4,507	0,4075	9,4174433	23,231226	67,351331	0,939476364

Supplementary Table S1. Raw data of egg quality parameters with ATOL (Animal Trait Ontology for Livestock) descriptors, for hen.

				ATOL_ 0001880	ATOL_ 0002297	ATOL_ 0002119	ATOL_ 0002126	ATOL_ 0002122	ATOL_ 0002074	ATOL_ 0002073	ATOL_ 0002075	ATOL_ 0005600	ATOL_ 0005600	ATOL_ 0001883	ATOL_ 0005587	ATOL_ 0001938	ATOL_ 1881	ATOL_ 0002121	ATOL_ 1881	ATOL_ 0001883	ATOL_ 0001882	ATOL_ 0002125
**Hen age**	**Pen**	**Genotype**	**Replicate**	**Egg weight**	**Egg diameter**	**Shell strength**	**Shell fracture**	**Shell modulus**	**Yolk lightness**	**Yolk redness**	**Yolk yellow**	**Blood spot**	**Meat spot**	**Yolk weight**	**Albumen pH**	**Albumen DM**	**Shell weight**	**Shell thickness**	**Shell**	**Yolk**	**Albumen**	**Shell-to-egg-compression**
wk	no.			g	mm	N	mm	N/mm	L*	a*	b*	1=present	1=present	g	-	% (w/w)	g	mm	% (w/w)	% (w/w)	% (w/w)	%
21	4	A	1	47,772	39,369999		0,174999	75,069244	59,34	6,22	58,6			10,156	9,45	13,878889	4,81	0,4325	10,068659	21,259315	68,672025	0,44449836
21	4	A	2	52,462	40,584999	40,380726	0,37249	75,062096	65,21	2,19	53,97			12,861	9,47	13,898889	5,598	0,4741667	10,670581	24,514887	64,814532	0,917802166
21	4	A	3	52,24	41,592499	63,95076	0,487492	93,827606	65,07	2,85	57,65			12,001	9,44	13,192437	6,199	0,5066667	11,866386	22,972818	65,160796	1,172067108
21	4	A	4	44,864	38,619999	53,291702	0,487495	63,808853	59,72	14,75	64,88	1		10,38	9,46	13,212437	4,902	0,4433333	10,926355	23,136591	65,937054	1,262286413
21	4	A	5	47,899	39,825001	44,584301	0,454998	56,291187	66,14	4,35	64,07			11,799	9,44	13,298373	4,72	0,4191667	9,8540679	24,633082	65,51285	1,14249338
21	4	A	6	49,941	40,547501	48,938	0,4925	67,549438	62,29	4,32	61,95		1	11,836	9,46	13,318373	5,183	0,4183333	10,378246	23,699966	65,921788	1,214624793
21	4	A	7	51,009	40,924999	32,874344	0,372486	82,584038	56,8	8,83	51,38			12,487	9,42	13,93247	5,204	0,4475	10,202121	24,479994	65,317885	0,910167402
21	4	A	8	50,621	41,209999	44,584301	0,377487	82,576172	64,5	2,66	60,89			12,528	9,44	13,95247	5,402	0,44	10,67146	24,748622	64,579917	0,916008273
21	4	A	9	58,372	43,3325	55,543613	0,457489	75,069244	70,35	0,56	61,49			11,869	9,41	14,793815	6,133	0,4725	10,50675	20,333379	69,159871	1,055764149
21	4	A	10	57,783	42,654999	54,943104	0,439999	82,57618	42,95	10,67	42,15			11,048	9,37	14,844374	6,533	0,4858333	11,306093	19,11981	69,574096	1,031529739
21	4	A	11	50,687	40,57	36,927792	0,412491	63,821037	62,02	3,36	59,5			12,574	9,49	13,599918	4,975	0,3983333	9,81514	24,80715	65,37771	1,01673897
21	4	A	12	52,243	41,782501	46,836212	0,442486	93,836555	50,75	8,74	54,66			12,541	9,52	14,300622	5,498	0,4391667	10,523898	24,00513	65,470972	1,059022293
21	4	A	13	51,462	42,177498	37,077919	0,394989	82,514282	59,43	6,94	52,69	1	1	9,309	9,33	14,787362	4,861	0,405	9,4458047	18,089075	72,46512	0,93649225
21	4	A	14	49,529	41,362499	53,141575	0,465	82,57618	65,05	0,24	59,97			11,41	9,47	14,188303	5,158	0,4508333	10,414101	23,037009	66,548891	1,124206736
21	4	A	15	51,117	40,369999	36,777664	0,429993	45,045837	61,48	9,28	60,34			12,289	9,48		4,956	0,4041667	9,6954047	24,040926	66,26367	1,065130073
21	5	B	1	56,209	42,162498	31,523197	0,362492	86,313164							9,44		5,109	0,4266667	9,0892918			0,859749818
21	5	B	2	48,039	40,837498	23,266178	0,24749	86,329628	64,31	1,24	56,65			9,899	9,48	13,294728	4,749	0,4233333	9,8857179	20,606174	69,508108	0,606036148
21	5	B	3	51,312	41,630001	40,080471	0,464985	82,576164	60,56	8,98	54,18			9,933	9,44	13,252728	4,467	0,3775	8,7055659	19,358045	71,936389	1,116946886
21	5	B	4	49,965	40,375	41,131363	0,457493	60,043945	56,48	7,02	66,21	1		10,409	9,48	13,716571	4,677	0,4283333	9,3605524	20,832583	69,806865	1,133109598
21	5	B	5	53,919	42,822498	18,612223	0,279991	67,568771	62,99	8,08	58,94	1		10,485	9,44	13,674571	4,04	0,3433333	7,4927206	19,445835	73,061444	0,653840885
21	5	B	6	52,964	42,099998	29,871794	0,404984	75,076393	60,06	5,05	55,12			11,849	9,51	14,22865	4,223	0,36	7,9733404	22,3718	69,65486	0,96195729
21	5	B	7	51,358	42,232502	38,429066	0,449993	71,30217	53,54	9,84	65,11			11,871	9,47	14,18665	4,373	0,3475	8,5147397	23,114218	68,371042	1,065513476
21	5	B	8	53,341	41,610001				56,42	5,05	49,17	1		12,47	9,39	12,955677	4,243	0,3416667	7,9544815	23,377889	68,667629	
21	5	B	9	48,634	40,890003	45,935448	0,467499	82,591927	62,9	2,28	57,54			10,432	9,35	12,913677	4,653	0,4041667	9,5673808	21,450014	68,982605	1,143308794
21	5	B	10	50,061	40,862499	36,177155	0,482498	71,287659	60,3	6,69	55,38			10,345	9,35	13,671255	4,208	0,3875	8,405745	20,664789	70,929466	1,180784367
21	5	B	11	51,941	41,75	28,370518	0,347488	82,568291	57,2	8,7	54,96		1	8,883	9,31	13,629255	4,44	0,3858333	8,5481604	17,102097	74,349743	0,832306587
21	5	B	12	50,981	42,322498	34,826004	0,402489	86,321396	58,67	9,37	56,69			11,734	9,45	13,789606	4,378	0,3533333	8,587513	23,016418	68,396069	0,95100483
21	5	B	13	55,582	42,322498	29,871794	0,414986	71,308975	55,81	4,66	57,94		1	11,912	9,41	13,747606	4,365	0,36	7,8532618	21,431399	70,715339	0,98053286
21	5	B	14	54,883	42,255001				65,45	6,48	62,11		1	11,994	9,3	14,512781	4,279	0,3375	7,7965855	21,853762	70,349653	
21	5	B	15	54,789	43,084999				55,98	11,44	47,03		1	10,53	9,3	14,470781	4,592	0,385	8,3812444	19,219186	72,399569	
21	6	C	1	52,505	42,182499	46,38583	0,422485	82,568283	65,08	3,98	61,96			11,62	9,51	14,090264	5,119	0,4075	9,7495477	22,131226	68,119227	1,001564654
21	6	C	2	44,438	39,0275	46,686085	0,439999	71,30899	51,29	11,37	41,74		1	9,331	9,47	14,048264	4,62	0,4216667	10,396507	20,997795	68,605698	1,127407597
21	6	C	3	48,704	41,16	46,085575	0,452492	82,568298	64,73	7,22	63			12,176	9,46	13,59219	4,62	0,3958333	9,4858739	25	65,514126	1,099348882
21	6	C	4	44,171	39,485001	46,235703	0,425003	60,049667	61,64	10,08	66,07	1		10,511	9,42	13,55019	4,528	0,4166667	10,25107	23,796156	65,952774	1,076365681
21	6	C	5	47,743	41,3325	54,192467	0,427486	90,09169	58,43	2,21	61,46			10,902	9,42	14,306714	5,363	0,4416667	11,23306	22,834761	65,932179	1,034261175
21	6	C	6	52,673	41,095001	49,088127	0,444996	78,822723	61,29	3,29	52,63			9,25	9,38	14,264714	5,457	0,4291667	10,360147	17,561179	72,078674	1,082847035
21	6	C	7	61,02	43,309998	47,586853	0,459991	78,830215	63,71	3,31	57,76			12,128	9,49	13,957098	5,994	0,4291667	9,8230088	19,875451	70,30154	1,062089636
21	6	C	8	47,632	40,9025	48,487617	0,452496	75,069244	62,31	3,58	56,26			10,256	9,45	13,915098	4,805	0,425	10,087756	21,531743	68,380501	1,106279567
21	6	C	9	55,272	43,224998	46,98634	0,467499	67,568764	57,43	3,07	53,83			12,502		13,021703	5,708	0,4291667	10,32711	22,619048	67,053843	1,081547765
21	6	C	10	54,89	41,794998	45,78532	0,424988	93,827599	58,99	4,77	52,47		1	10,131		12,979703	5,609	0,4258333	10,218619	18,456914	71,324467	1,016839384
21	6	C	11	47,777	40,485001	48,187363	0,454994	56,307301	61,37	4,19	54,34			9,357	9,48	13,823528	4,679	0,4008333	9,7934152	19,584737	70,621847	1,123858191
21	6	C	12	47,109	40,380001	44,734428	0,432491	56,301926	60,56	6,52	57,77			11,748	9,44	13,781528	4,513	0,3916667	9,5799104	24,93791	65,48218	1,071052475
21	6	C	13	50,511	41,327499	51,189915	0,407494	93,836555	60,86	6,7	57,36		1	10,624	9,45	13,668059	5,391	0,4391667	10,672923	21,033042	68,294035	0,986011759
21	6	C	14	44,608	39,067501	46,085575	0,39249	56,301926	62,18	2,16	58,33			9,857	9,41	13,626059	4,863	0,4458333	10,901632	22,096933	67,001435	1,00464578
21	6	C	15	55,497	42,66	44,734428	0,457497	78,815186	59,81	3,05	54,69			11,484	9,42		5,34	0,4283333	9,6221417	20,69301	69,684848	1,07242616

Supplementary Table S1. Raw data of egg quality parameters with ATOL (Animal Trait Ontology for Livestock) descriptors, for hen.

**Hen age**<br>wk	**Pen**<br>no.	**Genotype**	**Replicate**	ATOL_ 0001880<br>**Egg weight**<br>g	ATOL_ 0002297<br>**Egg diameter**<br>mm	ATOL_ 0002119<br>**Shell strength**<br>N	ATOL_ 0002126<br>**Shell fracture**<br>mm	ATOL_ 0002122<br>**Shell modulus**<br>N/mm	ATOL_ 0002074<br>**Yolk lightness**<br>L*	ATOL_ 0002073<br>**Yolk redness**<br>a*	ATOL_ 0002075<br>**Yolk yellow**<br>b*	ATOL_ 0005600<br>**Blood spot**<br>1=present	ATOL_ 0005600<br>**Meat spot**<br>1=present	ATOL_ 0001883<br>**Yolk weight**<br>g	ATOL_ 0005587<br>**Albumen pH**<br>-	ATOL_ 0001938<br>**Albumen DM**<br>% (w/w)	ATOL_ 1881<br>**Shell weight**<br>g	ATOL_ 0002121<br>**Shell thickness**<br>mm	ATOL_ 1881<br>**Shell**<br>% (w/w)	ATOL_ 0001883<br>**Yolk**<br>% (w/w)	ATOL_ 0001882<br>**Albumen**<br>% (w/w)	ATOL_ 0002125<br>**Shell-to-egg-compression**<br>%
21	7	A	1	54,315	41,952499	40,831108	0,407482	86,329628	52,23	2,79	62,45			12,435	9,5	14,525597	5,605	0,4475	10,319433	22,894228	66,786339	0,971293748
21	7	A	2	47,646	40,274998	47,887108	0,472496	56,301941	61,25	5,25	57,29		1	10,745	9,46	14,483597	4,883	0,4141667	10,248499	22,551736	67,199765	1,173174484
21	7	A	3	49,414	39,952499	58,396038	0,472496	63,814945	64,88	3,52	63,73			11,793	9,46	14,080269	5,429	0,4633333	10,986765	23,865706	65,147529	1,18264442
21	7	A	4	50,22	40,904999	53,892212	0,467495	86,329636	65,58	4,77	63,49			10,544	9,42	14,038269	5,092	0,4308333	10,139387	20,995619	68,864994	1,142879871
21	7	A	5	53,421	41,220001	39,029579	0,442497	82,576172	58,44	4,22	74,18			12,89	9,46	14,361813	4,62	0,375	8,6482844	24,129088	67,222628	1,073500702
21	7	A	6	55,663	42,645	47,586853	0,445	78,830215	60,53	3,99	57,95			12,77	9,42	14,319813	5,459	0,4375	9,8072328	22,941631	67,251136	1,043498652
21	7	A	7	51,003	40,577503	57,044891	0,469994	56,301926	65,02	7,52	59,36			10,99	9,5	14,162995	6,933	0,4925	13,593318	21,547752	64,85893	1,158262498
21	7	A	8	54,113	41,264999	44,284042	0,412483	86,313164	63,84	4,41	60,94			13,479	9,46	14,120995	5,742	0,4508333	10,611129	24,908987	64,479885	0,999595323
21	7	A	9	50,444	41,4375	65,452034	0,474995	101,3145	57,85	5,13	55,2		1	11,992	9,5	13,968368	5,714	0,4716667	11,327413	23,772897	64,899691	1,146292609
21	7	A	10	52,619	41,380001	53,591957	0,472492	75,069252	57,78	5,7	60,54			11,174	9,46	13,926368	5,506	0,4583333	10,463901	21,235675	68,300424	1,141836608
21	7	A	11	47,605	40,059998	59,146679	0,462486	71,308983	60,74	4,84	54,85		1	10,727	9,5	14,463962	5,505	0,4591667	11,563911	22,533347	65,902741	1,154483333
21	7	A	12	52,489	40,967499	46,085575	0,404987	97,590019	63,8	6,76	58,73			11,996	9,46	14,421962	5,692	0,4466667	10,844177	22,854312	66,301511	0,988556807
21	7	A	13	47,045	40,612499	52,541061	0,43499	63,814938	67,21	3,56	66,02			11,784	9,47	14,629637	4,963	0,4433333	10,549474	25,048358	64,402168	1,071074203
21	7	A	14	51,7	41,412498	51,940552	0,417492	101,34347	64,73	7,8	62,61	1		12,604	9,43	14,587637	5,761	0,4691667	11,143133	24,37911	64,477756	1,008130444
21	7	A	15	53,251	41,282501	50,139019	0,442497	82,57618	56,63	4,35	60,99			12,356	9,47		5,688	0,46	10,68149	23,20332	66,11519	1,071875466
21	8	D	1	52,139	41,130001	55,993996	0,440002	90,091698	65,03	6,8	60,9			11,035	9,33	13,873243	5,838	0,48	11,196993	21,164579	67,638428	1,069783587
21	8	D	2	52,863	41,947498	57,044891	0,434998	93,845505	65,69	5,07	58,86			11,702	9,29	13,831243	5,598	0,4383333	10,589637	22,136466	67,273897	1,03700583
21	8	D	3	49,663	41,432499	58,245911	0,417492	105,09694	61,5	1,53	54,3			10,877	9,39	14,234879	5,82	0,4933333	11,718986	21,901617	66,379397	1,007643782
21	8	D	4	53,052	41,720001	62,149231	0,452488	108,86079	65	3,37	59,45			12,149	9,35	14,192879	5,891	0,4858333	11,1042	22,900173	65,995627	1,084582908
21	8	D	5	51,28	41,605	60,197571	0,422489	101,33381	64,66	4,31	60,18		1	10,64	9,42	14,429233	6,24	0,5333333	12,168487	20,74883	67,082683	1,015476505
21	8	D	6	52,776	42,0275	56,294254	0,452488	90,083092	64,75	0,44	52,93			11,597	9,38	14,387233	5,679	0,5058333	10,760573	21,974003	67,265424	1,076647433
21	8	D	7	52,021	41,9925	37,378174	0,3825	86,329643	52,9	8,97	51,76			10,975	9,45	14,711886	5,194	0,4383333	9,9844294	21,097249	68,918321	0,910876942
21	8	D	8	50,1	41,3825	60,79808	0,439991	97,590019	58,11	3,71	62,59			10,314	9,41	14,669886	5,622	0,46	11,221557	20,586826	68,191617	1,063229626
21	8	D	9	51,349	40,9375	52,390934	0,484997	71,322578	65,53	7,71	63,42			11,8	9,45	13,799502	5,467	0,4466667	10,646751	22,98	66,37325	1,184725496
21	8	D	10	52,479	42,037498	55,393486	0,432487	90,09169	56,87	6,89	49,07			11,836	9,41	13,757502	5,912	0,4708333	11,265459	22,553783	66,180758	1,028812419
21	8	D	11	50,083	41,075001	56,294254	0,447495	101,34348	64,84	3,56	56,47			11,351	9,49	13,866122	5,631	0,4625	11,243336	22,664377	66,092287	1,089458281
21	8	D	12	52,912	42,705002	45,78532	0,387493	78,822723	63,76	4,52	56,61			12,564	9,45	13,824122	5,862	0,465	11,078772	23,745086	65,176142	0,90737146
21	8	D	13	54,113	42,392498	57,195019	0,444996	101,33382	48	7,33	48,36			10,799	9,42	13,371126	5,795	0,4608333	10,709072	19,956388	69,334541	1,049704596
21	8	D	14	52,402	41,862499	60,497826	0,469997	112,60385	60,73	6,96	57,75			12,295	9,38	13,329126	5,785	0,4641667	11,039655	23,462845	65,4975	1,122716061
21	8	D	15	53,138	41,224998	59,597061	0,447491	93,845505	62,46	7,35	55,77			10,763	9,37		5,872	0,465	11,050472	20,254808	68,694719	1,085484589
21	9	D	1	52,098	41,670002	41,131363	0,417492	86,329628	63,06	6,62	60,32			10,764	9,47	13,987667	4,958	0,4183333	9,5166801	20,661062	69,822258	1,0019006
21	9	D	2	52,158	41,029999	56,894764	0,457489	101,33382	57,69	0,47	53,49			11,647	9,43	13,945667	6,266	0,4975	12,013497	22,330227	65,656275	1,115010995
21	9	D	3	46,187	40,2575	52,390934	0,445	63,802773	58,19	7,77	64,38			11,354	9,43	13,105317	4,866	0,4425	10,535432	24,582675	64,881893	1,10538409
21	9	D	4	47,407	40,047501	53,141575	0,419998	75,069252	60,18	9,59	56,68			11,049			5,318	0,4566667	11,217753	23,306685	65,475563	1,048749584
21	9	D	5	48,158	39,9375	51,039787	0,447498	67,568764	69,49	2,67	64,83			9,9933	9,45	13,420668	5,337	0,4583333	11,082271	20,751069	68,16666	1,120495775
21	9	D	6	51,105	41,247498	60,79808	0,459995	93,827606	64,66	1,63	60,95			12,066	9,41	13,378668	5,542	0,4725	10,84434	23,610214	65,545446	1,115207036
21	9	D	7	51,71	41,564999	62,599613	0,429981	120,12227	61,28	7,02	54,29			12,276	9,43	13,553338	5,968	0,4825	11,541288	23,740089	64,718623	1,034478552
21	9	D	8	49,608	40,934998	53,141575	0,444988	101,33382	62,77	6	54,85			10,454	9,39	13,511338	5,066	0,4266667	10,212063	21,073214	68,714723	1,087060026
21	9	D	9	54,663	41,934998	56,144127	0,417496	101,33381	65,05	6,29	58,32			11,375	9,44	13,327315	5,906	0,4541667	10,804383	20,809323	68,386294	0,99557892
21	9	D	10	53,941	41,579998	64,251015	0,467495	108,84003	59,14	2,25	60,83			11,189	9,4	13,285315	6,262	0,5166667	11,60898	20,743034	67,647986	1,124326653
21	9	D	11	49,17	41,037502	47,586853	0,452488	90,074501	64,88	5,77	60,51			10,151	9,44	13,190417	4,874	0,4308333	9,9125483	20,644702	69,44275	1,10262072
21	9	D	12	47,219	39,697498	49,988892	0,432484	71,315781	60,74	2,18	64,14			9,449	9,4	13,148417	5,116	0,4416667	10,834622	20,011013	69,154366	1,089449013
21	9	D	13	49,533	41,310001	55,993996	0,45499	90,074501	59,86	7,42	52,17			10,96	9,43	13,446769	5,373	0,4533333	10,847314	22,126663	67,026023	1,101403992
21	9	D	14	53,207	41,709999	48,037235	0,449993	97,590012	63,68	5,5	57,88			12,605	9,39	13,404769	5,19	0,4341667	9,7543556	23,690492	66,555153	1,07886121
21	9	D	15	50,171	40,942501	37,678429	0,424992	97,580704	54,84	0,94	47,74			11,43	9,32		4,949	0,4291667	9,8642642	22,782085	67,353651	1,03802159

Supplementary Table S1. Raw data of egg quality parameters with ATOL (Animal Trait Ontology for Livestock) descriptors, for hen.

				ATOL_0001880	ATOL_0002297	ATOL_0002119	ATOL_0002126	ATOL_0002122	ATOL_0002074	ATOL_0002073	ATOL_0002075	ATOL_0005600	ATOL_0005600	ATOL_0001883	ATOL_0005587	ATOL_0001938	ATOL_1881	ATOL_0002121	ATOL_1881	ATOL_0001883	ATOL_0001882	ATOL_0002125
**Hen age**	**Pen**	**Genotype**	**Replicate**	**Egg weight**	**Egg diameter**	**Shell strength**	**Shell fracture**	**Shell modulus**	**Yolk lightness**	**Yolk redness**	**Yolk yellow**	**Blood spot**	**Meat spot**	**Yolk weight**	**Albumen pH**	**Albumen DM**	**Shell weight**	**Shell thickness**	**Shell**	**Yolk**	**Albumen**	**Shell-to-egg-compression**
wk	no.			g	mm	N	mm	N/mm	L*	a*	b*	1=present	1=present	g	-	% (w/w)	g	mm	% (w/w)	% (w/w)	% (w/w)	%
21	11	C	1	50,755	41,5075	42,032131	0,469994	93,836563	47,26	4,09	42,08			11,015	9,51	14,09306	4,667	0,4816667	9,1951532	21,702295	69,102551	1,132311028
21	11	C	2	48,175	39,695	51,340042	0,442497	67,555878	45,29	5,88	53,32	1		11,171	9,47	14,05106	5,196	0,4441667	10,785677	23,188376	66,025947	1,114742411
21	11	C	3	54,909	42,807499	43,23315	0,467487	71,322578	57,39	2,71	58,98			10,924	9,45	14,013042	5,056	0,4041667	9,2079623	19,894735	70,897303	1,092068004
21	11	C	4	52,741	41,829998	43,23315	0,429985	93,845505	62,78	4,27	55,07			11,615	9,41	13,971042	5,138	0,4183333	9,7419465	22,022715	68,235339	1,027934546
21	11	C	5	60,592	43,700001	54,492722	0,447495	67,568756	61,76	1,76	63,32			12,17	9,47	14,582463	6,233	0,4625	10,286837	20,08516	69,628004	1,024015995
21	11	C	6	54,162	41,110001	55,093231	0,482494	90,074501	54,46	7,77	51,99		1	12,942	9,43	14,540463	5,498	0,4525	10,151028	23,894982	65,95399	1,173665746
21	11	C	7	48,7	40,642498	36,177155	0,399982	75,06208	56,10	6,91	61,72			10,714	9,39	13,955714	4,257	0,3666667	8,7412731	22	69,258727	0,984147185
21	11	C	8	48,771	40,610001	45,78532	0,457493	82,568298	58,70	4,52	57,02			10,113	9,35	13,913714	4,785	0,4041667	9,8111583	20,735683	69,453159	1,126552546
21	11	C	9	58,74	43,427498	51,49017	0,447498	67,568764	53,78	4,25	59,75			10,56	9,41	13,71494	5,817	0,4333333	9,9029622	17,977528	72,11951	1,030448496
21	11	C	10	51,272	41,395	42,182259	0,409996	93,836555	56,41	2,97	49,17		1	12,95	9,37	13,67294	4,965	0,3925	9,683648	25,25745	65,058902	0,990448122
21	11	C	11	56,189	43,27	43,983788	0,484997	63,821037	61,25	1,62	63,66			13,35	9,48	14,302003	5,097	0,3916667	9,0711705	23,759099	67,169731	1,120862029
21	11	C	12	51,996	41,842499	50,439278	0,469997	93,836563	56,76	3,37	46,38			10,81	9,44	14,260003	5,427	0,4458333	10,437341	20,790061	68,772598	1,123252701
21	11	C	13	49,787	40,227501	40,981236	0,442501	63,808853	57,55	2,10	49,99			11,251	9,44	14,201108	4,729	0,3966667	9,4984635	22,598269	67,903268	1,099996244
21	11	C	14	51,349	41,087498	45,635193	0,469986	97,599327	56,44	0,33	44,17	1		9,926	9,4	14,159108	4,805	0,4066667	9,3575337	19,330464	71,312002	1,143866195
21	11	C	15	61,31	44,255001	47,887108	0,439991	78,830215	61,51	3,25	58,60			12,743	9,46		5,718	0,4083333	9,3263742	20,784538	69,889088	0,99421758
21	12	B	1	50,894	41,120003	43,383278	0,437489	101,34348	43,43	10,08	43,55	1	1	9,38	9,41	14,364077	4,678	0,4041667	9,1916532	18,430463	72,377883	1,063932315
21	12	B	2	53,763	42,457504	58,095783	0,497494	86,346107	53,95	8,68	66,33			10,786	9,45	14,406077	5,679	0,4558333	10,563027	20,062125	69,374849	1,171745753
21	12	B	3	51,629	41,092503	30,622431	0,417496	78,830215	61,47	5,63	60,62	1		11,245	9,27	13,404769	4,228	0,3841667	8,189196	21,780395	70,030409	1,015990678
21	12	B	4	48,805	40,852501	39,930344	0,417496	90,074501	61,20	8,79	62,19			11,111	9,31	13,446769	4,568	0,43	9,3596968	22,76611	67,874193	1,021959463
21	12	B	5	52,309	42,025005	46,98634	0,439995	93,827614	59,57	3,95	57,60	1	1	11,611	9,4	13,877977	4,923	0,4125	9,4113824	22,196945	68,391673	1,046983814
21	12	B	6	51,474	41,852501	41,882004	0,417496	105,08692	61,70	12,71	63,32			10,487	9,44	13,919977	4,927	0,4208333	9,5718227	20,373392	70,054785	0,997541342
21	12	B	7	50,606	40,564999	45,034683	0,437489	78,822708	52,00	11,43	61,69			11,356	9,38	13,170786	4,935	0,4391667	9,7518081	22,440027	67,808165	1,078488872
21	12	B	8	54,151	41,3125	46,38583	0,429993	93,845505	53,50	10,33	51,89			10,038	9,42	13,212786	5,172	0,415	9,5510702	18,537054	71,911876	1,040830257
21	12	B	9	57,548	42,57	25,818348	0,462494	52,525967	58,87	1,77	52,01			11,941	9,4	14,492336	3,972	0,3425	6,9020644	20,749635	72,348301	1,086431759
21	12	B	10	51,258	42,227501	53,892212	0,489994	82,584045	60,89	8,48	58,90			11,854	9,44	14,534336	4,818	0,4133333	9,3995084	23,126146	67,474345	1,160367032
21	12	B	11	52,762	40,372498	34,225494	0,464985	63,80278	51,54	8,85	59,70			11,695	9,38	13,511032	4,373	0,3891667	8,2881619	22,165574	69,546264	1,151737007
21	12	B	12	54,483	42,545002	46,686085	0,462486	75,069244	63,99	1,09	58,20	1		11,076	9,42	13,553032	5,055	0,4166667	9,2781235	20,329277	70,3926	1,087051306
21	12	B	13	50,988	41,829998	35,576641	0,429989	90,091698	48,98	8,59	60,79		1	10,506	9,41	13,278492	4,145	0,3616667	8,1293638	20,604848	71,265788	1,027944108
21	12	B	14	51,093	41,387501	50,139019	0,414997	97,590012	58,47	10,83	59,83			10,126	9,45	13,320492	5,395	0,4516667	10,559176	19,818762	69,622062	1,002710939
21	12	B	15	48,331	38,592499	34,675877	0,407486	71,308975	60,96	6,95	59,13			9,495	9,21		4,591	0,4333333	9,4990793	19,645776	70,855145	1,055868396
25	1	C	1	63,835	44,852501	51,039787	0,387505	86,321381	58,17	2,7	56,6			11,667	9,29	14,583542	6,651	0,4625	10,419049	18,276807	71,304143	0,863954052
25	1	C	2	58,041	43,047501	47,586853	0,417507	105,08691	60,69	-5,2	46,53			13,677	9,33	14,625542	5,868	0,4466667	10,110095	23,564377	66,325529	0,969875115
25	1	C	3	56,575	43,175003	49,388382	0,432495	108,86079	61,55	2,14	53,88			13,282	9,28	14,667129	5,54	0,4225	9,7923111	23,476801	66,730888	1,001725466
25	1	C	4	56,474	43,997498	43,23315	0,437489	78,822708	54,84	4,24	53,45			13,194	9,32	14,709129	5,119	0,405	9,0643482	23,362963	67,572688	0,994349724
25	1	C	5	58,104	43,264999	43,23315	0,409988	93,818657	61,42	0,4	55,45		1	14,22	9,38	13,594783	5,788	0,4141667	9,9614484	24,473358	65,565193	0,9476205
25	1	C	6	62,11	44,614998	52,090679	0,420002	82,576157	61,8	0,29	52,03			13,757	9,42	13,636783	6,178	0,3941667	9,9468685	22,149412	67,903719	0,941391951
25	1	C	7	60,046	43,919998	48,938	0,437492	63,808853	61,73	2,95	53,4			13,668	9,22	13,971614	5,93	0,435	9,8757619	22,762549	67,361689	0,996111156
25	1	C	8	57,163	43,230003	45,034683	0,394985	93,827606	63,7	-3,5	49,93	1		13,801	9,26	14,013614	5,955	0,4583333	10,417578	24,14324	65,439183	0,913682564
25	1	C	9	57,781	43,379997	45,485065	0,467503	71,308983	61,55	0,51	55,07			13,95	9,42	13,786667	5,341	0,3991667	9,243523	24,142884	66,613593	1,07769256
25	1	C	10	58,858	43,800003	42,482513	0,419998	67,562325	67,45	-3,02	54,04			14,17	9,46	13,828667	5,508	0,4241667	9,3581161	24,074892	66,566992	0,958899478
25	1	C	11	52,17	41,887505	43,83366	0,455002	101,32414							9,41	13,779821	4,82	0,3991667	9,2390263	0	90,760974	1,086247558
25	1	C	12	55,991	42,695	63,049995	0,452503	116,35734	66,14	-4,43	48,23			12,545	9,45	13,821821	6,31	0,4783333	11,269668	22,405387	66,324945	1,0598501
25	1	C	13	57,865	43,345001	51,640297	0,462498	90,077469	61,68	-0,77	50,2			13,921	9,39	13,776314	5,764	0,425	9,9611164	24,057721	65,981163	1,067015779
25	1	C	14	59,369	43,805	44,434174	0,432503	67,58165	64,52	-3,7	48,31	1		13,857	9,43	13,818314	5,804	0,4325	9,7761458	23,340464	66,88339	0,987337062
25	1	C	15	53,591	42,172501	41,731876	0,427494	90,100281	57,24	1,19	52,15			13,913	9,33		5,072	0,4058333	9,4642757	25,961449	64,574276	1,013679506

Supplementary Table S1. Raw data of egg quality parameters with ATOL (Animal Trait Ontology for Livestock) descriptors, for hen.

				ATOL_0001880	ATOL_0002297	ATOL_0002119	ATOL_0002126	ATOL_0002122	ATOL_0002074	ATOL_0002073	ATOL_0002075	ATOL_0005600	ATOL_0005600	ATOL_0001883	ATOL_0005587	ATOL_0001938	ATOL_1881	ATOL_0002121	ATOL_1881	ATOL_0001883	ATOL_0001882	ATOL_0002125
**Hen age**	**Pen**	**Genotype**	**Replicate**	**Egg weight**	**Egg diameter**	**Shell strength**	**Shell fracture**	**Shell modulus**	**Yolk lightness**	**Yolk redness**	**Yolk yellow**	**Blood spot**	**Meat spot**	**Yolk weight**	**Albumen pH**	**Albumen DM**	**Shell weight**	**Shell thickness**	**Shell**	**Yolk**	**Albumen**	**Shell-to-egg-compression**
wk	no.			g	mm	N	mm	N/mm	L*	a*	b*	1=present	1=present	g	-	% (w/w)	g	mm	% (w/w)	% (w/w)	% (w/w)	%
25	2	D	1	59,23	44,285004	38,128811	0,412495	63,821037	66,34	-1,4	54,77			13,363	9,23	13,797027	4,938	0,3891667	8,3369914	22,561202	69,101807	0,931455262
25	2	D	2	61,796	43,850006	56,594509	0,43499	75,069237	65,17	-0,88	56,55			14,737	9,27	13,839027	6,565	0,4741667	10,623665	23,847822	65,528513	0,991995303
25	2	D	3	60,677	44,389999	46,98634	0,439987	60,04966	67,14	-1,22	54,09			10,836	9,15	13,572429	5,347	0,3983333	8,8122353	17,858497	73,329268	0,991184974
25	2	D	4	61,521	43,6325	43,083023	0,462502	67,568771	68,97	-2,62	51,14			13,65	9,19	13,614429	4,997	0,3725	8,1224297	22,187546	69,690025	1,05999427
25	2	D	5	60,05	43,904999	59,446934	0,467487	78,822701	68,32	-4,8	47,75			12,929	9,34	13,213345	6,282	0,4516667	10,461282	21,530391	68,008326	1,064769413
25	2	D	6	58,298	44,215004	50,439278	0,429989	60,049671	69,54	-0,77	53,34			12,288	9,38	13,255345	6,135	0,4666667	10,523517	21,07791	68,398573	0,972495671
25	2	D	7	53,211	41,919998	55,243359	0,432499	116,34622	63,16	3,16	54,74			12,586	9,29	13,25853	5,45	0,435	10,242243	23,653004	66,104753	1,031724763
25	2	D	8	59,259	43,739998	57,795528	0,455002	71,315781	64,5	-1,49	52,88			12,225	9,33	13,30053	5,993	0,4533333	10,113232	20,629778	69,25699	1,040242389
25	2	D	9	55,776	43,057503	60,647953	0,467503	82,568283	63,34	-3,91	43,38			14,458	9,33	13,273798	6,08	0,4566667	10,900746	25,921543	63,177711	1,085764309
25	2	D	10	57,113	42,934998	47,887108	0,425007	86,337875	58,99	1,54	59,86			12,268	9,37	13,315798	5,635	0,4341667	9,8664052	21,480223	68,653371	0,989884756
25	2	D	11	57,624	43,202499	55,843868	0,4725	86,3461	64,38	-2,02	46,43			11,597	9,35	13,403756	6,239	0,455	10,827086	20,125295	69,047619	1,093686733
25	2	D	12	56,941	43,195	52,240807	0,440002	97,571396	61,05	-2,78	49,55			13,173	9,39	13,445756	5,739	0,4425	10,078854	23,134473	66,786674	1,018641046
25	2	D	13	55,04	42,120003	52,991444	0,415005	116,35734	65,86	-3,99	46,21			12,046	9,33	13,828057	5,713	0,3766667	10,379724	21,885901	67,734375	0,985291953
25	2	D	14	56,692	43,292503	60,347698	0,46249	101,34348	65,86	-3,99	46,21			14,035	9,37	13,870057	5,826	0,4316667	10,276582	24,756579	64,966838	1,0682912
25	2	D	15	50,365	41,184998		0,254982	71,329391	60,36	0,21	64,25			11,466	9,37		4,192	0,3608333	8,3232403	22,76581	68,91095	0,619113785
25	3	B	1	62,374	43,887497	28,520645	0,362507	60,038322	66,17	-2,69	55,43			14,817	9,15	13,264575	5,049	0,3725	8,094719	23,75509	68,150191	0,825991512
25	3	B	2	62,004	44,0625	38,729324	0,425007	56,307308	63,14	3,4	57,32			14,427	9,19	13,306575	5,47	0,4108333	8,8220115	23,267854	67,910135	0,964554894
25	3	B	3	55,232	42,787506	42,632641	0,412491	78,830215	67,76	-0,88	60,79			12,929	9,36	13,868973	5,458	0,425	9,8819525	23,408531	66,709516	0,964045439
25	3	B	4	55,533	42,282501	34,525749	0,359993	101,33381	65,48	0,54	58,16			13,062	9,4	13,910973	5,46	0,4275	9,8319918	23,52115	66,646859	0,851399495
25	3	B	5	61,608	44,590004	43,683533	0,395	78,822701	58,62	-0,35	62,42			14,414	9,31	12,757978	5,922	0,4283333	9,612388	23,396312	66,9913	0,885848766
25	3	B	6	57,75	43,180008	35,726768	0,429993	67,542999	60,09	4,81	57,56			13,384	9,35	12,799978	4,98	0,39	8,6233766	23,175758	68,200866	0,995815008
25	3	B	7	60,837	44	39,029579	0,384998	86,3461	58,41	4,18	61,36	1		13,18	9,36	12,983146	5,246	0,4033333	8,6230419	21,664448	69,71251	0,874995455
25	3	B	8	57,559	43,052498	32,724216	0,394997	82,584038	60,64	4,08	53,83			12,877	9,4	13,025146	4,869	0,3866667	8,4591463	22,371827	69,169027	0,917477541
25	3	B	9	62,681	44,217499	46,686085	0,467487	60,049671	61,8	-0,83	55,85			14,578	9,31	13,646891	5,667	0,4158333	9,0410172	23,257446	67,701536	1,057244328
25	3	B	10	59,036	44,097504	47,286594	0,452503	71,322578	64,3	0,69	56,46			14,233	9,35	13,688891	5,664	0,4083333	9,5941459	24,109018	66,296836	1,026141978
25	3	B	11	61,486	44,792503	48,33749	0,33749	93,827606	63,9	-1,96	52,56			14,372	9,2	11,356246	6,055	0,4358333	9,8477702	23,374427	66,777803	0,753451978
25	3	B	12	57,021	43,607498	43,533405	0,449997	72,064194	55,32	-1,56	47,49			13,919	9,24	11,398246	4,967	0,375	8,7108258	24,410305	66,878869	1,031925748
25	3	B	13	61,545	44,1325	43,983788	0,429996	60,049671	65,02	1,95	61,68			15,178	9,35	13,289836	5,746	0,4008333	9,336258	24,66163	66,002112	0,974329576
25	3	B	14	58,061	43,305				62,68	1,22	52,36			13,965	9,39	13,331836	5,618	0,4216667	9,6760304	24,05229	66,27168	
25	3	B	15	60,935	44,794998	55,543613	0,467491	78,830231	64,37	-2,73	50,86		1	14,037	9,4		5,816	0,4266667	9,5445967	23,036022	67,419381	1,043623219
25	4	A	1	54,988	41,599998	40,831108	0,397499	112,59315	53,89	4,78	61,51			13,217	9,38	13,952352	5,534	0,4266667	10,064014	24,036153	65,899833	0,955526488
25	4	A	2	56,595	42,290001	49,838764	0,462494	105,08692	60,53	0,79	54,3	1		14,3	9,42	13,994352	5,819	0,4508333	10,281827	25,26725	64,450923	1,093624945
25	4	A	3	55,456	42,269997				66,01	0,48	62,07			13,936	9,4	14,009718	5,356	0,4216667	9,6581073	25,129833	65,21206	
25	4	A	4	54,439	42,065002	44,734428	0,427498	97,590027	65,02	-1,71	53,71			12,247	9,44	14,051718	5,428	0,42	9,970793	22,496739	67,532468	1,016279519
25	4	A	5	54,745	41,962502	46,535957	0,417496	105,07689	60,36	0,52	59,77			13,972	9,38	13,575674	5,6	0,4475	10,229245	25,521965	64,24879	0,994926375
25	4	A	6	58,498	43,175003	50,28915	0,470005	82,552551	62,53	0,79	54,38			13,579	9,29	13,645798	5,662	0,4325	9,6789634	23,212759	67,108277	1,088604441
25	4	A	7	55,857	42,800003	48,787872	0,472504	93,836563	64,84	1,09	59,55			13,601	9,33	13,687798	5,323	0,4158333	9,5296919	24,349679	66,120629	1,103981231
25	4	A	8	56,85	43,527496	45,635193	0,464996	71,315781	54,11	-0,17	61,74		1	15,474	9,41	13,868943	5,524	0,4283333	9,7167986	27,218997	63,064204	1,06828107
25	4	A	9	62,828	43,597504	49,538509	0,377499	86,321381	62,6	4,62	54,7			13,734	9,28	14,504897	6,898	0,4866667	10,979181	21,85968	67,161138	0,865872964
25	4	A	10	61,743	43,547501	48,037235	0,429993	82,568306	55,42	7,55	66,17			12,887	9,38	14,439059	6,245	0,4491667	10,114507	20,872002	69,013491	0,987411425
25	4	A	11	68,524	46,290001	58,84642	0,4725	120,09934	63,29	5,3	60,8			14,223	9,43	14,61642	7,042	0,4658333	10,276691	20,756231	68,967077	1,020738798
25	4	A	12	54,903	42,192505	47,887108	0,467495	97,590027	59,03	-1,46	57,9			12,377	9,46	14,817992	5,496	0,4316667	10,010382	22,543395	67,446223	1,108004846
25	4	A	13	57,845	43,082497	53,44183	0,449997	97,565781	66,66	0,66	60,45			13,957	9,37		6,12	0,4533333	10,579998	24,128274	65,291728	1,04450074
25	4	A	14	56,192	42,862503	53,44183	0,479996	93,836563	65,93	-4,2	50,29			14,277	9,41		5,991	0,475	10,66166	25,407531	63,930809	1,119850607
25	4	A	15	56,288	42,785004	51,790424	0,449993	90,100281	67,25	-3,77	52,57			14,642	9,52		5,959	0,4758333	10,586626	26,012649	63,400725	1,051754021

Supplementary Table S1. Raw data of egg quality parameters with ATOL (Animal Trait Ontology for Livestock) descriptors, for hen.

				ATOL_ 0001880	ATOL_ 0002297	ATOL_ 0002119	ATOL_ 0002126	ATOL_ 0002122	ATOL_ 0002074	ATOL_ 0002073	ATOL_ 0002075	ATOL_ 0005600	ATOL_ 0005600	ATOL_ 0001883	ATOL_ 0005587	ATOL_ 0001938	ATOL_ 1881	ATOL_ 0002121	ATOL_ 1881	ATOL_ 0001883	ATOL_ 0001882	ATOL_ 0002125
**Hen age**	**Pen**	**Genotype**	**Replicate**	**Egg weight**	**Egg diameter**	**Shell strength**	**Shell fracture**	**Shell modulus**	**Yolk lightness**	**Yolk redness**	**Yolk yellow**	**Blood spot**	**Meat spot**	**Yolk weight**	**Albumen pH**	**Albumen DM**	**Shell weight**	**Shell thickness**	**Shell**	**Yolk**	**Albumen**	**Shell-to-egg-compression**
wk	no.			g	mm	N	mm	N/mm	L*	a*	b*	1=present	1=present	g	-	% (w/w)	g	mm	% (w/w)	% (w/w)	% (w/w)	%
25	5	B	1	56,733	42,355003	31,072815	0,377495	90,083107	61,7	4,22	61,14	1		13,183	9,25	13,538892	4,826	0,3775	8,506513	23,236917	68,25657	0,89126425
25	5	B	2	55,386	42,084999	30,021921	0,337502	93,827606	63,64	1,13	60,23			12,591	9,29	13,580892	4,829	0,3816667	8,7188098	22,733182	68,548009	0,801953209
25	5	B	3	59,908	42,779999	37,528301	0,397491	86,346107	60,85	1,98	56,32			13,661	9,32	13,370608	5,496	0,3975	9,1740669	22,803298	68,022635	0,929151494
25	5	B	4	55,999	42,527496	44,884556	0,487499	86,329628	59,17	1,1	58,3		1	12,315	9,36	13,412608	5,117	0,3991667	9,1376632	21,991464	68,870873	1,146314845
25	5	B	5	60,352	43,407501	45,034683	0,447506	82,560417	55,15	1,65	53,37			13,456	9,32	13,349474	5,687	0,4141667	9,4230514	22,295864	68,281084	1,030941634
25	5	B	6	58,217	43,652496	40,530853	0,462494	71,315788	57,74	3,22	68,93			14,166	9,36	13,391474	5,039	0,3833333	8,6555473	24,333099	67,011354	1,05949039
25	5	B	7	61,323	44,112503	47,136467	0,464996	71,322586	64,51	0,3	55,04		1	15,629	9,36	13,079137	5,773	0,4175	9,4140861	25,486359	65,099555	1,054113842
25	5	B	8	62,93	44,347504	43,683533	0,402512	78,830231	67,82	-2,04	59,98			13,781	9,4	13,121137	6,115	0,4333333	9,717146	21,898935	68,383919	0,90763169
25	5	B	9	57,573	42,840004	46,98634	0,409996	93,836555	59,86	0,33	52,87			13,822	9,38	12,908624	5,766	0,4366667	10,015111	24,007781	65,977107	0,95704006
25	5	B	10	59,779	43,577499	32,874344	0,372494	67,562325	60	3,95	55,83		1	15,633	9,42	12,950624	5,652	0,4166667	9,4548253	26,151324	64,393851	0,854785173
25	5	B	11	56,136	43,717499	34,375622	0,382484	67,568756	64	-1,52	56,6			14,118	9,37	13,512541	4,844	0,3666667	8,6290438	25,149637	66,22132	0,874899088
25	5	B	12	55,253	43,440002	25,818348	0,339996	67,568756	57,79	5,11	58,24			11,801	9,41	13,554541	4,561	0,3616667	8,2547554	21,358116	70,387128	0,782679522
25	5	B	13	56,819	43,315002	40,230598	0,447502	71,315781	61,23	5,54	51,68			12,716	9,35	13,04868	4,736	0,3733333	8,33524	22,379838	69,284922	1,033133971
25	5	B	14	57,127	42,580002	41,882004	0,43499	86,329636	64,41	-1,66	54,16			13,871	9,39	13,09068	5,252	0,4183333	9,1935512	24,280988	66,525461	1,021582855
25	5	B	15	59,125	43,222504	40,831108	0,447502	82,568291	61,55	1,44	61,08			13,406	9,33		5,47	0,4141667	9,2515856	22,673996	68,074419	1,035344921
25	6	C	1	59,536	44,155006	42,482513	0,392494	56,307301	63,49	-1,3	50,33		1	13,83	9,35	13,216925	6,12	0,4483333	10,279495	23,229643	66,490863	0,888900343
25	6	C	2	52,747	40,949997	35,726768	0,382492	90,065918	60,51	3,72	56,03			12,948	9,39	13,258925	5,03	0,3916667	9,5360874	24,547368	65,916545	0,934046466
25	6	C	3	62,494	43,872498	51,49017	0,452503	60,055389	64,16	-3,37	50,41			14,727	9,36	13,451746	6,2	0,4441667	9,9209524	23,565462	66,513585	1,031404685
25	6	C	4	60,759	43,794998	52,240807	0,464993	63,814945	62,75	3,99	58,06			14,152	9,4	13,493746	6,121	0,4558333	10,074228	23,292023	66,63375	1,061749107
25	6	C	5	60,307	42,790001	55,543613	0,465	97,599319	68,1	-0,65	53,97			13,913	9,41	13,198814	6,395	0,4525	10,604076	23,07029	66,325634	1,086702475
25	6	C	6	52,971	42,227501	57,345146	0,480003	112,58239	59,95	3,79	53,88			13,736	9,45	13,240814	5,545	0,43	10,467992	25,93117	63,600838	1,136707095
25	6	C	7	60,295	44,199997	47,73698	0,434998	67,568756	64,29	-2,63	48,09			14,134	9,37	14,027712	6,082	0,4458333	10,087072	23,441413	66,471515	0,984158438
25	6	C	8	54,959	42,695	48,787872	0,444984	90,09169	59,21	-1,72	55,56			12,25	9,41	14,069712	5,447	0,4283333	9,9110246	22,289343	67,799632	1,042239138
25	6	C	9	55,434	42,317497	43,083023	0,459999	82,584053	63,44	0,31	52,12			12,013	9,37	13,898822	5,042	0,4108333	9,095501	21,670816	69,233683	1,08701845
25	6	C	10	60,692	44,165001	23,266178	0,272491	60,043949	63,24	-1,45	49,46			13,198	9,41	13,940822	5,87	0,4225	9,6717854	21,745864	68,58235	0,616984023
25	6	C	11	54,422	41,82	44,284042	0,424999	93,836555	60,93	1,86	54,24			13,367	9,36	13,088541	5,428	0,4208333	9,9739076	24,561758	65,464334	1,016257771
25	6	C	12	55,707	42,279999	39,930344	0,409996	93,845505	60,02	2,27	48,66			13,698	9,4	13,130541	5,267	0,3908333	9,4548261	24,589369	65,955804	0,969716201
25	6	C	13	56,144	43,1325	57,345146	0,482491	90,09169	54,86	5,62	48,47			13,613	9,38	12,845192	6,03	0,4441667	10,740239	24,24658	65,01318	1,118625167
25	6	C	14	58,97	44,3675	46,085575	0,434994	75,076408	61,05	0,01	47,58			13,055	9,42	12,887192	5,63	0,4266667	9,5472274	22,138375	68,314397	0,980433876
25	6	C	15	54,278	42,447502	35,126259	0,394989	93,836563	60,73	2,66	53,8			13,546	9,37		4,949	0,3775	9,1178746	24,956704	65,925421	0,930535323
25	7	A	1	52,848	39,877502	42,332386	0,360001	82,568306	60,06	2,61	56,15			12,879	9,27	13,781595	5,448	0,4616667	10,30881	24,369891	65,321299	0,902767179
25	7	A	2	51,65	41,93	48,787872	0,432487	101,3435	58,68	3,08	53,31			13,092	9,31	13,823595	5,174	0,4391667	10,017425	25,347531	64,635044	1,031450036
25	7	A	3	53,27	41,184998	46,686085	0,422497	97,599327	57,93	7,33	61,06			14,959	9,41	13,499055	5,319	0,4275	9,9849822	28,081472	61,933546	1,025851695
25	7	A	4	51,868	42,18	51,640297	0,424992	120,11079	64,29	2,01	59,2	1		13,781	9,45	13,541055	5,397	0,4266667	10,40526	26,569368	63,025372	1,007567568
25	7	A	5	56,002	42,764999	51,039787	0,465	90,083107	65,93	-0,77	57,61		1	14,497	9,38	12,884318	5,885	0,4433333	10,508553	25,886575	63,604871	1,087337802
25	7	A	6	60,631	43,745003	38,128811	0,455006	67,562325	69,56	-2	57,28			13,906	9,42	12,926318	5,416	0,455	8,9327242	22,935462	68,131814	1,040132515
25	7	A	7	51,727	41,682503	45,034683	0,422493	105,117									5,193	0,4233333	10,039244			1,01359796
25	7	A	8	58,601	42,842499	31,673325	0,327492	97,580704	65,82	0,59	62,12			14,454	9,41	13,473262	5,593	0,4191667	9,5442057	24,665108	65,790686	0,764409191
25	7	A	9	54,677	41,122505	43,533405	0,447498	101,32257	64,49	1,79	62,13			15,318	9,45	13,515262	5,576	0,445	10,198072	28,015436	61,786492	1,088207054
25	7	A	10	56,562	42,467499	48,33749	0,410007	101,35313	62,57	3,78	62,22			14,452	9,38	13,922809	5,893	0,4491667	10,418656	25,550723	64,030621	0,965460669
25	7	A	11	60,864	43,1875	54,492722	0,499996	75,062088	64,57	2,04	58,19			15,324	9,42	13,964809	6,438	0,46	10,577681	25,177445	64,244874	1,15773314
25	7	A	12	58,068	43,239998	51,039787	0,457493	105,08692	65,6	-0,53	55,31			13,781	9,41	13,585771	6,011	0,4533333	10,351657	23,73252	65,915823	1,058031964
25	7	A	13	52,328	41,417503	45,184811	0,419998	101,34348	66,57	3,17	63,43			15,032	9,45	13,627771	5,228	0,425	9,9908271	28,726494	61,282678	1,014059201
25	7	A	14	60,419	43,642502	39,179707	0,467491	82,536789	67,25	-2,58	52,62			14,292	9,41		5,411	0,4116667	8,9557921	23,654811	67,389397	1,071182857
25	7	A	15	61,057	43,139999	50,139019	0,48	86,321396	66,75	-3,07	53,72			14,203	9,45		5,974	0,45	9,7842999	23,26187	66,95383	1,112656493

Supplementary Table S1. Raw data of egg quality parameters with ATOL (Animal Trait Ontology for Livestock) descriptors, for hen.

				ATOL_ 0001880	ATOL_ 0002297	ATOL_ 0002119	ATOL_ 0002126	ATOL_ 0002122	ATOL_ 0002074	ATOL_ 0002073	ATOL_ 0002075	ATOL_ 0005600	ATOL_ 0005600	ATOL_ 0001883	ATOL_ 0005587	ATOL_ 0001938	ATOL_ 1881	ATOL_ 0002121	ATOL_ 1881	ATOL_ 0001883	ATOL_ 0001882	ATOL_ 0002125
**Hen age**	**Pen**	**Genotype**	**Replicate**	**Egg weight**	**Egg diameter**	**Shell strength**	**Shell fracture**	**Shell modulus**	**Yolk lightness**	**Yolk redness**	**Yolk yellow**	**Blood spot**	**Meat spot**	**Yolk weight**	**Albumen pH**	**Albumen DM**	**Shell weight**	**Shell thickness**	**Shell**	**Yolk**	**Albumen**	**Shell-to-egg-compression**
wk	no.			g	mm	N	mm	N/mm	L*	a*	b*	1=present	1=present	g	-	% (w/w)	g	mm	% (w/w)	% (w/w)	% (w/w)	%
25	8	D	1	57,875	43,387497	61,39859	0,467495	101,36282	66,45	-2,53	53,27			13,509	9,33	14,269922	6,25	0,4641667	10,799136	23,341685	65,859179	1,07748783
25	8	D	2	58,894	42,995003	59,597061	0,43499	105,0769	69,92	-2,99	53,35			13,619	9,37	14,311922	6,302	0,475	10,700581	23,124597	66,174823	1,011722223
25	8	D	3	60,004	43,370003	54,042339	0,44001	90,074501	63	-3,98	53,69			11,981	9,36	13,327059	6,224	0,4633333	10,372642	19,967002	69,660356	1,014549157
25	8	D	4	60,038	44,197502	56,894764	0,445	78,830231	57,71	0,99	64,22			12,551	9,4	13,369059	6,172	0,4666667	10,280156	20,905093	68,814751	1,006844233
25	8	D	5	55,003	41,607498	54,642849	0,407505	120,1108	63,23	1,25	54,57			12,616	9,39	13,20711	5,929	0,465	10,779412	22,936931	66,283657	0,979402799
25	8	D	6	57,049	43,772499	59,296806	0,447514	86,321404	57,5	2,32	60,92			14,734	9,43	13,24911	6,068	0,46	10,63647	25,826921	63,536609	1,022363379
25	8	D	7	57,87	43,567497	56,294254	0,450001	101,33382	66,37	-2,31	52,86			13,656	9,31	13,682443	5,983	0,4508333	10,33869	23,597719	66,063591	1,03288238
25	8	D	8	59,648	43,394997	53,892212	0,410004	90,09169	67,45	-2,78	56,6			13,734	9,35	13,724443	6,466	0,4791667	10,840263	23,02508	66,134657	0,944818593
25	8	D	9	59,391	44,017502	59,296806	0,464989	82,584045	66,21	-1,53	54,94			15,297	9,3	13,915358	6,171	0,4508333	10,390463	25,756428	63,853109	1,056372985
25	8	D	10	55,127	43,110001	60,948208	0,429989	105,07689	61,26	-3,78	51,09			13,407	9,34	13,957358	6,361	0,5	11,53881	24,320206	64,140984	0,997422849
25	8	D	11	56,257	42,445007	58,245911	0,422504	131,3837	66,28	-1,75	56,22			12,11	9,33	13,158916	6,389	0,495	11,356809	21,52621	67,116981	0,995415079
25	8	D	12	58,232	42,747498	51,039787	0,397495	108,8504	61,68	-2,61	44,93			12,124	9,37	13,200916	6,16	0,4725	10,578376	20,820168	68,601456	0,929867287
25	8	D	13	57,468	42,635002	49,988892	0,439991	112,60388	65,14	-2,25	50,45			12,517	9,37	13,119467	5,927	0,445	10,313566	21,780817	67,905617	1,031994792
25	8	D	14	64,45	45,252502	58,245911	0,442497	63,808846	58,04	1,7	62,19			14,291	9,41	13,161467	6,573	0,4766667	10,198604	22,173778	67,627618	0,977839855
25	8	D	15	58,793	43,6175	53,742085	0,445007	90,083084	69,96	-3,25	49,85			12,616	9,31		6,323	0,4725	10,754682	21,458337	67,786981	1,020248753
25	9	D	1	56,855	43	44,584301	0,404991	97,590027	64,33	2,02	58,55			14,519	9,23	13,047182	5,45	0,4191667	9,5857884	25,536892	64,877319	0,941839535
25	9	D	2	56,51	42,792503	60,497826	0,470005	101,34348	66,83	1,98	56,39			12,169	9,27	13,089182	6,07	0,4616667	10,741462	21,534242	67,724297	1,098334912
25	9	D	3	54,405	42,095001	50,28915	0,395004	112,59312	68,91	-2,52	52			12,931	9,31	13,609042	5,733	0,45	10,537634	23,768036	65,69433	0,938363204
25	9	D	4	58,99	43,782501		0,462494	82,568306	66,42	-0,327	52,51			13,883	9,35	13,651042	6,088	0,4566667	10,320393	23,534497	66,145109	1,056344406
25	9	D	5	55,39	42,352501	60,197571	0,467499	120,13369	64,94	3,81	57,56			12,305	9,36	13,257454	5,805	0,4666667	10,480231	22,215201	67,304568	1,103828555
25	9	D	6	59,358	43,695	45,935448	0,419998	78,822708	64,69	0,21	61,59		1	12,685	9,4	13,299454	5,776	0,4391667	9,7307861	21,370329	68,898885	0,961203799
25	9	D	7	55,668	42,8825	53,141575	0,452492	105,08692	67,28	1	55,23			13,462	9,36	13,667549	5,643	0,4366667	10,136883	24,182654	65,680463	1,055190346
25	9	D	8	56,778	43,157501	57,795528	0,470001	105,08692	57,56	1,3	63,89			13,258	9,4	13,709549	6,195	0,4725	10,910916	23,350594	65,73849	1,089036643
25	9	D	9	62,75	43,827499	54,642849	0,454994	75,054924	65,43	-0,31	56,29			14,224	9,33	12,768648	6,702	0,4733333	10,680478	22,667729	66,651793	1,038147306
25	9	D	10	58,969	43,445	61,548717	0,452492	93,872375	63,63	2,17	58,93			12,488	9,37	12,810648	6,277	0,4766667	10,644576	21,177229	68,178195	1,041528369
25	9	D	11	56,297	43,077499	56,744637	0,477505	97,571396	55,53	3,48	57,5			12,171	9,36	12,922316	5,891	0,4475	10,464146	21,619269	67,916585	1,10847893
25	9	D	12	56,084	41,417503	44,133915	0,382484	112,61462	61,07	-5,33	38,99			12,117	9,4	12,964316	5,948	0,455	10,60552	21,605092	67,789387	0,923483968
25	9	D	13	58,43	42,552498	52,090679	0,429996	116,35733	62,75	2,34	51,21			13,084	9,33	13,185286	6,129	0,4625	10,489475	22,392607	67,117919	1,010507068
25	9	D	14	61,63	44,195	52,390934	0,409985	71,315773	61,98	3,64	58,19			12,721	9,37	13,227286	6,265	0,4533333	10,165504	20,640922	69,193575	0,927672814
25	9	D	15	55,464	42,450005	51,340042	0,419998	105,0769	65,94	-1,52	52,53			12,39	9,39		5,887	0,45	10,614092	22,338814	67,047094	0,989394465
25	11	C	1	55,573	43,222504	46,38583	0,46249	93,845512	59,19	0,28	53,18			12,571	9,42	13,760015	5,206	0,3983333	9,3678585	22,620697	68,011444	1,070021302
25	11	C	2	57,845	42,252502	39,630089	0,3825	105,07689	62,96	7,28	60,98			13,572	9,44	13,780015	5,721	0,4258333	9,8902239	23,462702	66,647074	0,905271835
25	11	C	3	55,73	41,610001	49,988892	0,450005	93,827606	60,22	8,31	57,42			13,607	9,46	14,209385	5,787	0,4466667	10,383994	24,415934	65,200072	1,081482791
25	11	C	4	58,878	42,782501		0,447502	67,562325	59,81	-0,93	45,73			13,268	9,48	14,229385	5,585	0,425	9,4857162	22,534733	67,979551	1,04599308
25	11	C	5	54,2	42,537506	40,680981	0,41	105,11698	62,44	-1,06	50,62			13,231	9,39	13,699467	4,944	0,3866667	9,1217712	24,411439	66,46679	0,963855286
25	11	C	6	59,997	43,332497	37,378174	0,382504	101,33382	65,63	1,53	60,81	1		13,237	9,43	13,741467	5,85	0,4275	9,7504875	22,06277	68,186743	0,882718575
25	11	C	7	58,333	43,529999	57,044891	0,43248	93,854462	66,04	-3,79	47,19			12,386	9,36	13,566265	6,277	0,4666667	10,760633	21,233264	68,006103	0,993521732
25	11	C	8	57,376	43,510002	49,688637	0,452496	93,818657	60,75	4,22	55,26			13,977	9,4	13,608265	5,264	0,4158333	9,1745678	24,36036	66,465073	1,039981566
25	11	C	9	58,275	43,485001	48,33749	0,4575	82,576172	69,33	-1,15	63,59			14,598	9,36	13,778027	5,67	0,42	9,7297297	25,050193	65,220077	1,052086902
25	11	C	10	53,719	42,512497	40,831108	0,392502	105,10696	59,97	5,32	59,02			13,664	9,4	13,820027	4,949	0,4183333	9,2127553	25,436065	65,351179	0,923262635
25	11	C	11	60,229	43,93	40,831108	0,40749	78,837746	65,61	1,13	59,02			13,698	9,4	13,637285	5,693	0,4125	9,4522572	22,743197	67,804546	0,927589347
25	11	C	12	59,719	43,447502	46,535957	0,414989	86,329636	64,54	0,4	57,6	1		13,037	9,46	13,697285	6,01	0,4375	10,063799	21,830573	68,105628	0,955150425
25	11	C	13	60,099	44,5075	56,744637	0,427494	86,321396	55,71	2,9	51,42			13,053	9,35	13,7353	5,898	0,445	9,8138072	21,719163	68,467029	0,960498792
25	11	C	14	58,254	43,805	37,228046	0,39999	75,062096	54,31	4,01	60,31			15,224	9,39	13,7773	5,156	0,4091667	8,8508944	26,133828	65,015278	0,913114941
25	11	C	15	60,443	44,482498	51,49017	0,439991	82,57618	58,4	3,49	53,31		1	13,704	9,44		5,848	0,4341667	9,6752312	22,672601	67,652168	0,98913285

Supplementary Table S1. Raw data of egg quality parameters with ATOL (Animal Trait Ontology for Livestock) descriptors, for hen.

				ATOL_ 0001880	ATOL_ 0002297	ATOL_ 0002119	ATOL_ 0002126	ATOL_ 0002122	ATOL_ 0002074	ATOL_ 0002073	ATOL_ 0002075	ATOL_ 0005600	ATOL_ 0005600	ATOL_ 0001883	ATOL_ 0005587	ATOL_ 0001938	ATOL_ 1881	ATOL_ 0002121	ATOL_ 1881	ATOL_ 0001883	ATOL_ 0001882	ATOL_ 0002125
**Hen age**	**Pen**	**Genotype**	**Replicate**	**Egg weight**	**Egg diameter**	**Shell strength**	**Shell fracture**	**Shell modulus**	**Yolk lightness**	**Yolk redness**	**Yolk yellow**	**Blood spot**	**Meat spot**	**Yolk weight**	**Albumen pH**	**Albumen DM**	**Shell weight**	**Shell thickness**	**Shell**	**Yolk**	**Albumen**	**Shell-to-egg-compression**
wk	no.			g	mm	N	mm	N/mm	L*	a*	b*	1=present	1=present	g	-	% (w/w)	g	mm	% (w/w)	% (w/w)	% (w/w)	%
25	12	B	1	61,178	42,735001	38,879452	0,397491	101,34348	56,05	4,14	49,59			12,684	9,4	13,362905	5,711	0,4366667	9,3350551	20,732943	69,932002	0,930129848
25	12	B	2	57,331	41,912498	35,8769	0,362492	112,6146	58,66	8,3	55,84	1		13,506	9,42	13,382905	5,593	0,4433333	9,7556296	23,557935	66,686435	0,864878061
25	12	B	3	63,235	43,532501	40,981236	0,399994	82,576172	62,16	1,59	57,77	1		14,649	9,37	13,314624	6,015	0,4325	9,5121373	23,165968	67,321895	0,918839926
25	12	B	4	61,08	43,705002	23,566435	0,324986	67,549438	52,19	7,98	59,25		1	12,828	9,39	13,334624	3,968	0,3166667	6,4963982	21,001965	72,501637	0,743589944
25	12	B	5	58,77	43,342499	52,240807	0,469994	97,580711	66,48	-1,36	53,03	1		13,986	9,34	13,605374	5,663	0,4216667	9,6358686	23,797856	66,566275	1,084372177
25	12	B	6	55,93	41,57	46,686085	0,470005	93,83654	60,83	5,88	56,87			14,607	9,38	13,647374	5,569	0,4325	9,9570892	26,116574	63,926336	1,130635073
25	12	B	7	57,901	42,860001	42,632641	0,447498	90,091698	60,91	-0,48	56,13			13,789	9,37	13,245444	5,3	0,3966667	9,1535552	23,814787	67,031657	1,044092369
25	12	B	8	56,652	42,402496	46,535957	0,41	101,3628	65,32	2,46	62,24			14,045	9,41	13,287444	5,743	0,4558333	10,13733	24,791711	65,07096	0,966924211
25	12	B	9	54,945	41,824997	40,680981	0,417492	108,84003	57,05	6,93	56,32			14,332	9,39	13,72147	5,182	0,4083333	9,4312494	26,084266	64,484484	0,998187758
25	12	B	10	61,215	44,160004	43,983788	0,4575	71,315788	61,26	2,4	54,73		1	14,193	9,43	13,76347	5,568	0,4133333	9,0958099	23,185494	67,718696	1,036005341
25	12	B	11	57,552	42,517502	41,581749	0,402504	93,836563	63,76	-0,5	53,47			13,267	9,36	13,113989	5,506	0,4175	9,5670003	23,052196	67,380803	0,946678382
25	12	B	12	60,481	44,559998	41,131363	0,419994	64,063576	56,44	5,54	61,35			14,08	9,42	13,173989	5,242	0,3941667	8,6671847	23,280038	68,052777	0,942535949
25	12	B	13	59,616	43,862503	44,584301	0,402493	78,837738	58,5	7,77	58,16			14,038	9,33	13,541146	5,468	0,4191667	9,1720344	23,54737	67,280596	0,917624332
25	12	B	14	59,953	43,709999	50,589405	0,422497	82,591919	59,63	5,61	61,16			12,941	9,37	13,583146	5,766	0,4366667	9,6175337	21,585242	68,797224	0,966591191
25	12	B	15	59,905	42,389999	48,037235	0,412495	101,32414	60,88	8,4	59,34			13,863	9,35		5,989	0,4583333	9,997496	23,141641	66,860863	0,973095093
30	1	C	1	66,697	45,737503	38,128586	0,364986	71,308983	52,84	0,09	50,71	1		16,772	9,5	13,401196	6,43	0,4383333	9,6406135	25,146558	65,212828	0,798001587
30	1	C	2	66,51	46,375	38,578968	0,342495	105,0769	58,2	-0,67	63,34	1		16,673	9,52	13,421196	6,488	0,44	9,7549241	25,068411	65,176665	0,738533693
30	1	C	3	59,281	43,697502	49,988667	0,424995	75,076408	63,81	1,38	56,54			14,946	9,53	13,739506	5,758	0,4166667	9,7130615	25,212125	65,074813	0,972584199
30	1	C	4	65,021	44,1325	44,734203	0,389992	63,802773	70,02	-4,3	51,54			15,891	9,55	13,759506	6,42	0,445	9,8737331	24,439796	65,686471	0,88368436
30	1	C	5	67,681	44,762501	26,268505	0,309998	60,043949	63,42	0,1	56,16			15,751	9,42	13,900971	5,557	0,3825	8,2105761	23,27241	68,517014	0,692539499
30	1	C	6	63,978	44,735001	46,68586	0,399986	63,802773	56,49	2,36	64,95			16,614	9,46	13,942971	5,91	0,4133333	9,2375504	25,968302	64,794148	0,89412315
30	1	C	7	66,381	45,517502	54,792751	0,449993	71,315773	59,58	0,71	58,69			16,005	9,54	13,275089	6,523	0,4391667	9,826607	24,110815	66,062578	0,988615324
30	1	C	8	65,341	44,872501	51,640072	0,41	78,830215	65,62	-4,31	48,58			16,335	9,58	13,317089	6,536	0,4441667	10,002908	24,999617	64,997475	0,913699907
30	1	C	9	60,39	43,2075	40,530628	0,422493	67,568764	56,18	0,41	60,61			15,581	9,54	13,094942	6,198	0,4516667	10,263289	25,800629	63,936082	0,977823295
30	1	C	10	57,769	43,5825	44,283817	0,379986	75,069237	60,55	-2,59	56,5		1	14,219	9,58	13,136942	5,648	0,4016667	9,7768699	24,613547	65,609583	0,871877474
30	1	C	11	64,473	44,252502	45,034458	0,382488	60,055401	67,15	-3,65	49,68			16,492	9,53	13,908473	6,546	0,4533333	10,153087	25,5797	64,267213	0,86433079
30	1	C	12	64,663	44,800003	46,986115	0,382484	75,062096	62,2	-1,8	47,21			15,778	9,59	13,968473	6,338	0,445	9,8015867	24,400353	65,798061	0,853758871
30	1	C	13	62,89	45,475002	47,436497	0,419998	75,069244	59,67	-0,52	56,42			13,807	9,48	13,848159	5,595	0,4	8,8964859	21,954206	69,149308	0,923579948
30	1	C	14	55,852	42,975002	36,777439	0,447502	60,061119	64,65	-1,85	56,71			16,497	9,52	13,890159	4,77	0,375	8,5404283	29,536991	61,922581	1,041307689
30	1	C	15	60,015	43,785004	44,433945	0,414989	63,802773	55,98	2,38	47,79		1	14,268	9,52		5,791	0,4208333	9,6492544	23,774056	66,576689	0,947787969
30	2	D	1	60,786	43,6525	47,286369	0,397488	71,322594	56,62	-0,61	60,19			15,446	9,61	13,086961	5,889	0,425	9,6880861	25,410456	64,901458	0,910573278
30	2	D	2	63,275	44,775002	59,596836	0,402489	82,584045	56,84	-3,13	56,21			16,239	9,63	13,106961	6,47	0,4508333	10,225207	25,664164	64,110628	0,898914533
30	2	D	3	58,967	43,572502	54,492496	0,435001	75,06208	69,94	-5,62	47,53			16,527	9,55	13,292398	6,038	0,455	10,239626	28,027541	61,732834	0,998338356
30	2	D	4	59,391	43,650002	55,693516	0,402485	86,321404	68,56	-4,8	44,06			15,605	9,57	13,312398	6,488	0,475	10,924214	26,275025	62,800761	0,922073268
30	2	D	5	63,334	43,397503	48,787647	0,444996	67,55188	61,9	-3,91	49,53			13,337	9,53	13,027206	6,637	0,4916667	10,479363	21,058199	68,462437	1,025395401
30	2	D	6	62,394	44,095001	54,192242	0,432491	63,802773	66,78	2,05	55,9			16,546	9,57	13,069206	6,573	0,4758333	10,534667	26,518576	62,946758	0,980816397
30	2	D	7	63,861	44,727501	48,937775	0,432499	67,562325	69,6	-3,37	54,29			15,641	9,38	13,358765	6,038	0,4216667	9,4549099	24,492257	66,052833	0,966964374
30	2	D	8	56,821	43,122505				61,03	-3,54	44,5			12,194	9,42	13,400765	5,147	0,4083333	9,0582707	21,460376	69,481354	
30	2	D	9	63,517	44,467499	52,991219	0,399986	86,321404	69,91	-0,47	52,28			14,216	9,42	13,120609	6,416	0,4516667	10,101233	22,38141	67,517358	0,899501904
30	2	D	10	64,037	44,7225	53,741859	0,429993	75,06208	67,21	-0,29	53,03			14,398	9,46	13,162609	6,131	0,4425	9,5741524	22,483877	67,941971	0,961469059
30	2	D	11	57,203	43,34	44,433945	0,409985	75,062096	68,56	-2,64	54,5			16,21	9,49	13,273995	5,568	0,4241667	9,7337552	28,337675	61,92857	0,945973696
30	2	D	12	56,167	43,072502	52,841091	0,4375	75,076408	65,38	-2,78	52,28				9,55	13,333995	6,13	0,4541667	10,913882			1,015729246
30	2	D	13	63,162	43,639999	57,495049	0,457489	67,555885	65,7	-3,04	50,96			13,183	9,56	12,93295	6,534	0,4283333	10,344828	20,871727	68,783446	1,048324955
30	2	D	14	59,963	43,767502	60,797855	0,444996	75,053894	63,7	-2,83	57,89			14,878	9,6	12,97495	6,262	0,4641667	10,443107	24,811967	64,744926	1,016726977
30	2	D	15	63,2	44,612499	54,942879	0,414993	78,830231	69,07	-2,71	53,82			16,188	9,53		6,609	0,4716667	10,457278	25,613924	63,928797	0,930216888

Supplementary Table S1. Raw data of egg quality parameters with ATOL (Animal Trait Ontology for Livestock) descriptors, for hen.

				ATOL_ 0001880	ATOL_ 0002297	ATOL_ 0002119	ATOL_ 0002126	ATOL_ 0002122	ATOL_ 0002074	ATOL_ 0002073	ATOL_ 0002075	ATOL_ 0005600	ATOL_ 0005600	ATOL_ 0001883	ATOL_ 0005587	ATOL_ 0001938	ATOL_ 1881	ATOL_ 0002121	ATOL_ 1881	ATOL_ 0001883	ATOL_ 0001882	ATOL_ 0002125
**Hen age**	**Pen**	**Genotype**	**Replicate**	**Egg weight**	**Egg diameter**	**Shell strength**	**Shell fracture**	**Shell modulus**	**Yolk lightness**	**Yolk redness**	**Yolk yellow**	**Blood spot**	**Meat spot**	**Yolk weight**	**Albumen pH**	**Albumen DM**	**Shell weight**	**Shell thickness**	**Shell**	**Yolk**	**Albumen**	**Shell-to-egg-compression**
wk	no.			g	mm	N	mm	N/mm	L*	a*	b*	1=present	1=present	g	-	% (w/w)	g	mm	% (w/w)	% (w/w)	% (w/w)	%
30	3	B	1	67,663	45,275002	43,53318	0,464996	60,055397	67,21	-0,28	58,6	1		17,247	9,5	13,690281	5,955	0,4091667	8,8009695	25,489559	65,709472	1,027047994
30	3	B	2	64,756	45,462502	43,833435	0,459995	78,822708					1		9,52	13,710281	5,673	0,405	8,7605782			1,011811888
30	3	B	3	58,607	43,032501	39,479736	0,394997	75,062096	63,77	-0,56	48,44			14,923	9,5	13,31008	5,33	0,3975	9,0944768	25,462829	65,442695	0,917903889
30	3	B	4	60,86	43,430004	39,329609	0,477497	63,802773	57,99	-1,15	64,45			18,449	9,52	13,33008	5,128	0,3908333	8,4258955	30,313835	61,260269	1,099463403
30	3	B	5	64,134	44,132504	36,777439	0,412487	52,54847	62,42	2,72	58,05			17,062	9,47	12,821713	5,52	0,3891667	8,6069791	26,603674	64,789347	0,934655781
30	3	B	6	57,082	43,715004	44,13369	0,432491	71,315781	69,62	-1,43	59,96			16,135	9,51	12,863713	5,067	0,3933333	8,8767037	28,266354	62,856943	0,98934224
30	3	B	7	63,578	44,432503				64,95	-1,62	59,51			18,614	9,52	13,155044	5,671	0,4008333	8,9197521	29,277423	61,802825	
30	3	B	8	56,295	42,8675	34,225269	0,382488	63,814945	64,96	-0,74	55,5			15,285	9,56	13,197044	5,143	0,4133333	9,1358025	27,151612	63,712585	0,892256371
30	3	B	9	59,68	42,577499	32,723991	0,397491	71,308983	63,38	2,27	58,54			14,837	9,57	13,39831	5,271	0,4025	8,8321046	24,860925	66,306971	0,93357057
30	3	B	10	63,184	44,1325	35,426289	0,404987	48,790363	68,35	-0,67	57,16			16,452	9,61	13,44031	5,363	0,3766667	8,4879083	26,038238	65,473854	0,917661587
30	3	B	11	59,671	43,442501	42,632416	0,427498	71,315781	60,23	-1	63,87	1		14,774	9,53		5,86	0,435	9,8205158	24,759096	65,420388	0,984054762
30	3	B	12	66,695	45,067501	30,622206	0,382484	52,54847	65,68	0,26	60,55			15,789	9,59		5,476	0,3933333	8,2105105	23,673439	68,116051	0,848691389
30	3	B	13	66,27	42,782501	33,624756	0,419998	71,322578	68,65	-0,48	64,36			15,441	9,55	13,159901	5,858	0,4158333	8,8395956	23,300136	67,860269	0,981705113
30	3	B	14	65,133	42,632504	36,176929	0,452499	67,562325	67,37	-0,87	59,77			14,977	9,59	13,201901	5,933	0,4183333	9,1090538	22,994488	67,896458	1,061394376
30	3	B	15	67,204	45,172501	30,321951	0,389988	63,808853	62,1	0,14	53,19			15,734	9,54		5,527	0,3975	8,2242128	23,412297	68,36349	0,863330547
30	4	A	1	59,345	43,834999	46,385605	0,409985	67,555878	66,5	-0,92	58,07		1	16,171	9,48	14,216232	5,902	0,4383333	9,9452355	27,249136	62,805628	0,935291455
30	4	A	2	64,098	44,424999	52,540836	0,417484	75,054924	58,85	-3,35	47,03			14,043	9,24	15,178704	6,073	0,4416667	9,4745546	21,90864	68,616806	0,939750162
30	4	A	3	58,774	42,387501	43,232925	0,479996	41,230125	65,89	-0,67	56,23			16,385	9,62	13,648355	6,025	0,4466667	10,251131	27,877973	61,870895	1,132399855
30	4	A	4	60,055	43,8125		0,367493	45,009125	67,04	0,41	62,68	1	1	13,916	9,28	15,220704	3,441	0,2925	5,7297477	23,172092	71,09816	0,838785735
30	4	A	5	64,055	43,877502	53,591732	0,429993	67,568771	59,36	-0,89	64,27			17,233	9,57	13,887339	6,739	0,4791667	10,520646	26,903442	62,575911	0,979985141
30	4	A	6	56,91	42,845001	43,833435	0,499989	67,557503	65,64	1,14	58,97			16,068	9,61	13,929339	5,267	0,4	9,254964	28,234054	62,510982	1,166971615
30	4	A	7	55,062	42,07	55,693516	0,48	97,580711	64,19	2,29	58,24			15,922	9,55	14,184934	6,147	0,5025	11,163779	28,916494	59,919727	1,14095555
30	4	A	8	59,388	43,8675	46,08535	0,424992	71,315781	64,77	-1,76	51,91			16,262	9,59	14,226934	5,587	0,4208333	9,4076244	27,382636	63,209739	0,968808343
30	4	A	9	64,094	45,055	61,69862	0,414986	82,584053	62,1	4,67	57,93			17,242	9,56	14,041029	6,989	0,4991667	10,904297	26,901114	62,194589	0,921065365
30	4	A	10	64,09	43,852501	51,18969	0,439987	71,315781	66,82	-2,11	52,32			15,882	9,6	14,613146	6,661	0,4766667	10,393197	24,780777	64,826026	1,003333881
30	4	A	11	60,926	43,7425	54,192242	0,457493	67,549438	69	2,83	64,15		1	17,385	9,64	14,655146	6,39	0,4633333	10,488133	28,534616	60,977251	1,045877579
30	4	A	12	65,065	44,635002	47,736755	0,369987	67,562317	62,51	2,88	58,47			16,831	9,39	14,802175	6,563	0,46	10,086836	25,867978	64,045186	0,828916732
30	4	A	13	57,179	42,837502	51,790199	0,489994	71,322586	64,76	3,11	59,06			16,323	9,58	13,821221	5,437	0,4175	9,5087357	28,547194	61,94407	1,143843542
30	4	A	14	62,986	44,68	60,047218	0,45499	71,308983	58,55	0,42	61,35			15,374	9,62	13,863221	6,606	0,4758333	10,488045	24,408599	65,103356	1,018330349
30	4	A	15	64,099	45,107502	53,441605	0,454994	63,821037							9,57		6,581	0,4583333	10,266931		89,733069	1,008688089
30	5	B	1	68,844	45,485001	40,380501	0,402489	60,055397	67,62	-3,84	53,23			16,149	9,58	12,766079	6,658	0,4216667	9,6711405	23,457382	66,871478	0,884882909
30	5	B	2	65,341	44,642502	48,487392	0,450001	67,55587	71,15	-3,89	58,21	1		16,253	9,6	12,786079	6,191	0,4325	9,4749086	24,874122	65,65097	1,008010259
30	5	B	3	64,65	45,045002	37,528076	0,422485	60,061111	63,45	1,2	59,48			16,4	9,53	13,979637	5,168	0,37	7,9938128	25,367363	66,638824	0,937917596
30	5	B	4	65,128	45,060001	36,327057	0,412491	63,808853	64,3	-2,37	51,86			17,205	9,55	13,999637	5,391	0,3816667	8,2775458	26,417209	65,305245	0,915426078
30	5	B	5	62,456	43,997501	31,372845	0,392494	56,279167	64,53	-1,56	56,92			16,161	9,56	13,500545	5,525	0,3883333	8,8462277	25,875817	65,277956	0,892082484
30	5	B	6	64,196	44,495003	34,525524	0,399986	56,263069	63,17	-1,59	49,22			15,962	9,6	13,542545	5,854	0,3966667	9,1189482	24,864478	66,016574	0,898945888
30	5	B	7	57,814	42,93	30,472078	0,449993	63,773735	64,16	-2,2	53,44	1		14,795	9,57	13,110628	4,569	0,35	7,9029301	25,590687	66,506383	1,048201724
30	5	B	8	64,419	44,147499	46,235477	0,439999	67,549431							9,61	13,152628	6,067	0,425	9,4180288			0,996656685
30	5	B	9	67,926	44,137501	42,632416	0,482498	52,525967	65,71	0,13	53,74			16,082	9,5	13,528618	6,252	0,4283333	9,2041339	23,675765	67,120101	1,093170182
30	5	B	10	65,549	45,162502	40,830883	0,454998	56,285835	58,1	2,08	53,03				9,54	13,570618	5,232	0,3741667	7,9818151			1,007468541
30	5	B	11	61,6	43,352501	33,174377	0,40749	63,80278	60,23	4,54	57,24	1		15,305	9,55	13,519921	5,478	0,3941667	8,8928571	24,845779	66,261364	0,939945772
30	5	B	12	61,075	43,845001	46,235477	0,465	67,562325	64,49	-3,27	47,3			17,771	9,61	13,579921	6,696	0,4116667	10,963569	29,097012	59,939419	1,060554201
30	5	B	13	65,445	44,342503	49,988667	0,462482	60,061119	67,06	-0,63	57,44			16,888	9,64	13,452971	6,22	0,4216667	9,5041638	25,804874	64,690962	1,042976758
30	5	B	14	63,638	44,32				58,98	3,47	62,68	1		15,615	9,68	13,494971	5,747	0,4108333	9,0307678	24,537226	66,432006	
30	5	B	15	60,48	43,162502	39,029354	0,467487	63,804153	65,25	-2,59	57,44	1	1	17,034	9,58		5,475	0,4091667	9,0525794	28,164683	62,782738	1,083085962

Supplementary Table S1. Raw data of egg quality parameters with ATOL (Animal Trait Ontology for Livestock) descriptors, for hen.

				ATOL_ 0001880	ATOL_ 0002297	ATOL_ 0002119	ATOL_ 0002126	ATOL_ 0002122	ATOL_ 0002074	ATOL_ 0002073	ATOL_ 0002075	ATOL_ 0005600	ATOL_ 0005600	ATOL_ 0001883	ATOL_ 0005587	ATOL_ 0001938	ATOL_ 1881	ATOL_ 0002121	ATOL_ 1881	ATOL_ 0001883	ATOL_ 0001882	ATOL_ 0002125
**Hen age**	**Pen**	**Genotype**	**Replicate**	**Egg weight**	**Egg diameter**	**Shell strength**	**Shell fracture**	**Shell modulus**	**Yolk lightness**	**Yolk redness**	**Yolk yellow**	**Blood spot**	**Meat spot**	**Yolk weight**	**Albumen pH**	**Albumen DM**	**Shell weight**	**Shell thickness**	**Shell**	**Yolk**	**Albumen**	**Shell-to-egg-compression**
wk	no.			g	mm	N	mm	N/mm	L*	a*	b*	1=present	1=present	g	-	% (w/w)	g	mm	% (w/w)	% (w/w)	% (w/w)	%
30	6	C	1	54,587	42,510002	44,283817	0,46249	75,069244	59,67	-2,48	55,02			14,179	9,59	13,100632	5,362	0,4175	9,8228516	25,975049	64,202099	1,087955724
30	6	C	2	64,563	46,022499	50,138794	0,449993	90,074516	62,39	-0,17	58,1			14,853	9,61	13,120632	6,314	0,4425	9,7795951	23,005437	67,214968	0,977767418
30	6	C	3	62,499	44,7075	52,841091	0,439999	67,562317	60,53	0,79	63,22			14,981	9,57	14,487397	6,139	0,4241667	9,8225572	23,969984	66,207459	0,984172678
30	6	C	4	60,425	43,32	36,777439	0,394993	78,809563	62,41	1,59	58,2			14,215	9,59	14,507397	5,769	0,4208333	9,5473728	23,525031	66,927596	0,911802862
30	6	C	5	62,858	44,57	50,739307	0,469997	75,020142	62,74	2,91	58,63			15,496	9,57	13,961609	5,924	0,4291667	9,4244169	24,652391	65,923192	1,054514247
30	6	C	6	57,271	42,872505	31,973354	0,439987	56,301941	60,45	0,06	53,05			15,72	9,61	14,003609	5,002	0,3708333	8,7339142	27,448447	63,817639	1,026268467
30	6	C	7	58,423	42,762501	39,479736	0,452496	67,562325	62,07	0,15	50,57			16,51	9,58	13,608738	5,319	0,4091667	9,1042911	28,259418	62,636291	1,058160747
30	6	C	8	55,683	42,227501	34,075142	0,4175	71,315781	65,21	-0,69	53,67			15,177	9,62	13,650738	4,871	0,3683333	8,7477327	27,256075	63,996193	0,98869218
30	6	C	9	61,181	45,1175	47,436497	0,432491	67,549431	57,23	-2,98	52,95		1	15,419	9,59	13,963962	5,791	0,415	9,4653569	25,202269	65,332374	0,958588131
30	6	C	10	64,248	45,025002	46,986115	0,40749	63,808853	65,55	-0,69	51,01			14,991	9,63	14,005962	6,276	0,445	9,7683975	23,333022	66,898581	0,905030498
30	6	C	11	57,704	43,465	49,688412	0,44249	78,830231	68,91	-2,7	50,65			16,111	9,41	14,408122	5,977	0,4483333	10,358034	27,920075	61,721891	1,018037501
30	6	C	12	63,173	44,080006	34,225269	0,427498	52,543453	65,07	-3,2	50,67		1	14,902	9,47	14,468122	5,155	0,3775	8,1601317	23,589192	68,250677	0,969822917
30	6	C	13	59,515	43,295002	43,53318	0,502491	60,066845	53,57	3,52	47,47		1	14,014	9,54	13,129893	5,749	0,4166667	9,6597496	23,547005	66,793245	1,160621265
30	6	C	14	63,844	45,732506	44,13369	0,455002	71,322586	64,3	-1,86	53,68			16,332	9,58	13,171893	5,672	0,3891667	8,8841551	25,581104	65,534741	0,994920331
30	6	C	15	60,433	43,3125	48,937775	0,437489	86,321396	66,73	0,9	57,34			14,129	9,66		5,998	0,4316667	9,925041	23,37961	66,695349	1,010075613
30	7	A	1	56,799	42,822502	42,932671	0,467503	71,30336									5,402	0,4308333	9,5107308	0	90,489269	1,091722758
30	7	A	2	56,711	41,895	37,678204	0,432487	93,814041	67,21	-0,36	60,16			16,537	9,66	13,303499	5,024	0,3925	8,8589515	29,160128	61,980921	1,032311732
30	7	A	3	60,512	43,139999				66,35	-0,75	60,02			17,266	9,68	13,323499	5,72	0,4191667	9,4526705	28,533184	62,014146	
30	7	A	4	57,579	42,695	45,48484	0,464989	75,076393	66,26	-2,03	52,19			13,818	9,39	13,935313	5,298	0,41	9,2012713	23,998333	66,800396	1,089094742
30	7	A	5	58,243	43,760002	52,540836	0,442501	75,069244	63,04	-1,68	51,92		1	15,393	9,41	13,955313	5,927	0,4516667	10,17633	26,428927	63,394743	1,01119968
30	7	A	6	59,201	43,52	38,428841	0,482498	56,268688	66,51	-2,27	54,77			14,621	9,5	14,123968	5,18	0,405	8,7498522	24,697218	66,55293	1,108681066
30	7	A	7	57,487	42,834999	48,63752	0,464985	71,315773	66,79	-0,26	59,69			14,029	9,54	14,165968	5,922	0,445	10,301459	24,403778	65,294762	1,08552588
30	7	A	8	57,514	42,279999	40,98101	0,442497	78,822701	67,28	-3,34	58,01			16,82	9,6	13,333816	5,714	0,4366667	9,9349724	29,245053	60,819974	1,046587064
30	7	A	9	56,731	42,222504	36,477184	0,427494	75,069244	57,54	-3,39	43,24			16,104	9,64	13,375816	5,649	0,4333333	9,9575188	28,386596	61,655885	1,012479033
30	7	A	10	59,489	43,290001	52,841091	0,499989	63,808853	52,99	-2,98	42,14			15,756	9,6	14,103294	5,725	0,4141667	9,6236279	26,485569	63,890803	1,154975718
30	7	A	11	60,475	44,502502	39,179482	0,449993	60,049873	58,95	-0,31	67,82			16,604	9,64	14,145294	5,44	0,4125	8,9954527	27,455974	63,548574	1,011163372
30	7	A	12	60,078	43,322502	43,53318	0,447498	71,292099	66,79	-0,85	57,1			17,373	9,62	14,208042	5,845	0,4358333	9,7290189	28,917407	61,353574	1,032945881
30	7	A	13	55,309	42,900002	27,91991	0,439991	56,256931	55,71	-1,54	60,18		1	16,455	9,68	14,268042	4,238	0,3433333	7,6624058	29,751035	62,586559	1,025619999
30	7	A	14	59,261	42,487499	51,489944	0,502499	86,265152	63,17	-0,99	56,82		1	17,289	9,54	13,648065	5,54	0,4108333	9,3484754	29,174331	61,477194	1,182698469
30	7	A	15	55,589	41,955002	48,337265	0,467499	78,762703	55,9	0,67	64,05			16,648	9,58	13,690065	5,86	0,455	10,541654	29,948371	59,509975	1,114286683
30	8	D	1	59,619	43,510002	48,787647	0,477497	71,277565	70,3	-6,92	52,55		1	15,768	9,62	13,067182	5,856	0,4333333	9,8223721	26,447944	63,729683	1,097441917
30	8	D	2	57,469	43,23	49,238029	0,504993	71,255058	69,71	-1,64	62,18			14,341	9,64	13,087182	6,041	0,4416667	10,511754	24,954323	64,533923	1,16815406
30	8	D	3	61,856	43,702503	36,777439	0,382488	67,534195	67,82	-1,99	55,18			15,936	9,55	13,584633	5,709	0,4125	9,2295008	25,763063	65,007437	0,875208452
30	8	D	4	56,2	43,027504	60,497601	0,469986	75,069237	63,84	1,04	56,63			15,352	9,57	13,604633	5,965	0,4558333	10,613879	27,316726	62,069395	1,092292037
30	8	D	5	61,218	44,555	52,841091	0,46999	7,495672	67,29	-1,58	58,66		1	13,193	9,47	14,244335	6,02	0,4441667	9,833709	21,550851	68,61544	1,054853552
30	8	D	6	59,425	43,830002	53,591732	0,469994	75,025757	69,25	-2,55	57,29			15,908	9,51	14,286335	5,798	0,4375	9,7568363	26,769878	63,473286	1,072311153
30	8	D	7	63,476	46,275002	50,439053	0,452496	116,35169	67,97	-3,19	50,07			14,47	9,45	13,561343	5,878	0,42	9,2601928	22,796017	67,94379	0,977841125
30	8	D	8	51,954	41,460003	50,288921	0,427486	108,84003	69,65	-4,94	30,59			14,113	9,49	13,603343	5,326	0,4416667	10,251376	27,164415	62,584209	1,031080485
30	8	D	9	53,21	41,370007	50,138794	0,454983	97,590019	65,83	-4,43	50,2			14,787	9,52	10,096773	5,258	0,4158333	9,8816012	27,789889	62,32851	1,099789517
30	8	D	10	55,048	43,41	53,741859	0,479992	75,04673	70,02	-1,71	59,46			13,271	9,56	10,138773	5,546	0,4291667	10,074844	24,108051	65,817105	1,105717577
30	8	D	11	56,274	42,702499	37,978458	0,432491	86,295876	66,71	0,31	46,34			13,742	9,48	13,518164	5,199	0,4183333	9,2387248	24,419803	66,341472	1,012800211
30	8	D	12	62,532	44,137501	58,095558	0,457493	67,555885	65,5	-1,15	52,71			15,038	9,54	13,578164	6,149	0,4591667	9,8333653	24,048487	66,118148	1,036517677
30	8	D	13	59,56	43,375	50,889435	0,419994	82,584045	67,81	1,41	57,36			15,762	9,58	12,524721	6,423	0,47	10,784083	26,46407	62,751847	0,968285879
30	8	D	14	59,379	43,41	58,54594	0,439999	82,568298	61,99	-0,29	56,3			16,826	9,62	12,566721	6,638	0,4741667	11,179036	28,336617	60,484346	1,013589035
30	8	D	15	58,833	44,220001	45,184586	0,447498	63,814945	69,39	-4,03	51,5			15,727	9,55		5,39	0,4083333	9,161525	26,731596	64,106879	1,011980981

Supplementary Table S1. Raw data of egg quality parameters with ATOL (Animal Trait Ontology for Livestock) descriptors, for hen.

				ATOL_ 0001880	ATOL_ 0002297	ATOL_ 0002119	ATOL_ 0002126	ATOL_ 0002122	ATOL_ 0002074	ATOL_ 0002073	ATOL_ 0002075	ATOL_ 0005600	ATOL_ 0005600	ATOL_ 0001883	ATOL_ 0005587	ATOL_ 0001938	ATOL_ 1881	ATOL_ 0002121	ATOL_ 1881	ATOL_ 0001883	ATOL_ 0001882	ATOL_ 0002125
**Hen age**	**Pen**	**Genotype**	**Replicate**	**Egg weight**	**Egg diameter**	**Shell strength**	**Shell fracture**	**Shell modulus**	**Yolk lightness**	**Yolk redness**	**Yolk yellow**	**Blood spot**	**Meat spot**	**Yolk weight**	**Albumen pH**	**Albumen DM**	**Shell weight**	**Shell thickness**	**Shell**	**Yolk**	**Albumen**	**Shell-to-egg-compression**
wk	no.			g	mm	N	mm	N/mm	L*	a*	b*	1=present	1=present	g	-	% (w/w)	g	mm	% (w/w)	% (w/w)	% (w/w)	%
30	9	D	1	60,981	43,490002	55,543388	0,432491	93,836555	60,83	-0,31	60,26			13,151	9,51	13,958136	6,483	0,4675	10,63118	21,565734	67,803086	0,99446075
30	9	D	2	57,761	44,0975	49,388157	0,43499	60,055389	62,3	-4,64	46,88			14,644	9,53	13,978136	5,664	0,44	9,8059244	25,352747	64,841329	0,986427802
30	9	D	3	59,465	43,522499	58,846195	0,427486	90,083092	67,98	-4,86	44,63			14,21	9,62	12,861972	6,49	0,4741667	10,913983	23,89641	65,189607	0,982218415
30	9	D	4	58,54	43,587502	59,596836	0,457493	75,070778	63,97	-2	54,19			16,014	9,64	12,881972	6,424	0,4683333	10,973693	27,355654	61,670653	1,04959674
30	9	D	5	58,652	43,307499	63,500153	0,489994	93,827606							9,54	13,615364	6,34	0,4741667	10,809521	0	89,190479	1,131429917
30	9	D	6	59,098	43,892502	53,591732	0,424995	71,315781	65,09	-0,12	56,04			15,738	9,58	13,657364	6,243	0,4625	10,563809	26,630343	62,805848	0,968263327
30	9	D	7	59,951	42,950001	56,894539	0,46249	78,817078	68,01	-2,98	50,69			14,882	9,61	13,272944	6,336	0,4575	10,568631	24,823606	64,607763	1,076810219
30	9	D	8	57,38	42,687504	53,141346	0,49749	67,494804	65,4	-5,21	47,56			14,782	9,65	13,314944	5,834	0,4441667	10,167306	25,761589	64,071105	1,165423024
30	9	D	9	59,163	43,889999	44,13369	0,407482	71,30336	65,99	-1,32	51,92			15,544	9,62	13,181158	5,646	0,4316667	9,5431266	26,273177	64,183696	0,928416517
30	9	D	10	60,087	43,0825	56,444157	0,462486	82,576172	64,18	-1,5	54,5			16,16	9,66	13,223158	6,575	0,4791667	10,942467	26,894337	62,163197	1,073489236
30	9	D	11	59,247	43,8675	53,141346	0,452492	78,813354	69,03	-4,92	33,61			16,252	9,48	12,576838	6,119	0,455	10,327949	27,430925	62,241126	1,031497122
30	9	D	12	61,077	44,215	33,174377	0,409988	52,53722	63,12	-1,47	54,52			17,431	9,54	12,636838	4,878	0,4383333	7,9866398	28,539385	63,473975	0,92725998
30	9	D	13	59,838	43,4375	60,797855	0,467487	71,315781	67,51	-0,34	56,82			12,961	9,5	13,61689	6,347	0,47	10,606972	21,660149	67,732879	1,076229065
30	9	D	14	61,67	43,697502	53,141346	0,409988	78,822708	60,78	-1,64	59,73			14,752	9,54	13,65889	6,398	0,47	10,374574	23,920869	65,704557	0,938241275
30	9	D	15	60,648	44,1875	47,436497	0,402489	71,308975	63,62	-2,62	55,44			13,458	9,54		5,654	0,45	9,3226487	22,190344	68,487007	0,910866195
30	11	C	1	60,52	44,677502	29,721441	0,359985	71,322586	63,9	4,5	57,52		1	14,666	5,58	13,534106	5,022	0,3683333	8,2980833	24,233311	67,468605	0,805741109
30	11	C	2	58,858	43,362499	42,031906	0,452496	71,293274	65,21	-2,15	53,79			15,031	5,6	13,554106	5,374	0,4116667	9,1304496	25,537735	65,331816	1,043519194
30	11	C	3	61,565	44,415001	22,365189	0,267498	67,562325	63,83	-1,3	57,72			16,52	9,48	13,821926	5,482	0,3975	8,90441	26,833428	64,262162	0,60226949
30	11	C	4	60,465	43,260002	50,739307	0,447491	82,576172	69,22	-2,91	61,4			16,264	9,5	13,841926	6,038	0,4283333	9,9859423	26,898206	63,115852	1,034422051
30	11	C	5	61,058	44,120003	36,026802	0,387493	60,061111	57,95	2,07	56,82			15,038	9,43	14,230687	5,193	0,3841667	8,505028	24,629041	66,865931	0,878270566
30	11	C	6	61,556	43,639999	37,978458	0,407482	63,80278	64,29	0,8	58,6		1	17,168	9,47	14,272687	5,359	0,3866667	8,7058938	27,890051	63,404055	0,933735127
30	11	C	7	60,739	44,022499	48,487392	0,402485	82,576172	64,45	-1,86	54,56			17,327	9,56	13,915044	5,648	0,4125	9,2988031	28,526976	62,174221	0,914271132
30	11	C	8	61,61	44,3125	41,281265	0,422493	56,301926	62,81	0,6	55,3			17,016	9,6	13,957044	5,622	0,4091667	9,125142	27,618893	63,255965	0,953439774
30	11	C	9	62,388	44,822502	40,830883	0,41	56,312668	64,97	-1,54	53,86			15,452	9,52	14,078617	5,4	0,3866667	8,6555107	24,767584	66,576906	0,914719129
30	11	C	10	60,191	44,82	40,380501	0,42749	60,055389	65,25	-4,3	46,51		1	16,681	9,56	14,120617	5,069	0,3675	8,4215248	27,713446	63,86503	0,95379295
30	11	C	11	57,481	42,447502	46,68586	0,449997	78,822708	67,39	0,02	54			14,647	9,61	13,77138	5,46	0,4191667	9,4987909	25,481463	65,019746	1,060125988
30	11	C	12	60,204	43,485001	41,731651	0,452496	71,304527	63,99	0,16	58,37			15,088	9,67	13,83138	5,516	0,4033333	9,1621819	25,061458	65,77636	1,040579486
30	11	C	13	58,084	43,247501	43,383053	0,462502	78,822708	66,06	-2,69	48,55			14,314	9,6	14,125165	5,154	0,4016667	8,8733558	24,64362	66,483025	1,069430578
30	11	C	14	58,102	42,755001	53,141346	0,437485	90,09169	61,4	-3,35	56,4			15,653	9,64	14,167165	6,307	0,4575	10,855048	26,940553	62,204399	1,023237024
30	11	C	15	60,305	43,880001	50,58918	0,452496	71,30217	65,74	-2,35	58,82			14,502	9,59		5,681	0,415	9,4204461	24,047757	66,531797	1,031212374
30	12	B	1	67,995	43,244999	34,525524	0,427486	67,555878					1	15,658	9,51	13,610448	6,064	0,4333333	8,9183028	23,028164	68,053533	0,988521239
30	12	B	2	57,887	42,032501	44,584076	0,422489	97,580704	63,84	-0,1	58,32			16,291	9,53	13,630448	5,642	0,4241667	9,7465752	28,142761	62,110664	1,005148373
30	12	B	3	62,125	45,072502	48,787647	0,459999	56,296574	61,99	-0,25	55,67			16,148	9,58	14,169104	5,581	0,4075	8,983501	25,992757	65,023742	1,020575694
30	12	B	4	65,408	45,202499	43,683308	0,447495	67,575203	63,15	1,49	64,16	1		16,448	9,6	14,189104	5,66	0,4041667	8,6533757	25,146771	66,199853	0,989978452
30	12	B	5	60,288	44,0825	42,482288	0,399994	60,055401	63,44	0,95	57,34			15,886	9,57	13,302464	5,745	0,4708333	9,5292596	26,350186	64,120555	0,907375943
30	12	B	6	66,228	43,990002	42,932671	0,424999	60,05571	62,95	-2,34	49,16		1	15,98	9,61	13,344464	6,152	0,455	9,2891224	24,128767	66,58211	0,966126348
30	12	B	7	65,987	43,767502	44,884331	0,437489	63,7976	65,4	3,65	63,18			17,016	5,54	13,036912	6,452	0,4533333	9,7776835	25,786897	64,435419	0,999574981
30	12	B	8	66,804	45,685001	34,975906	0,467491	78,807678	62,86	-0,25	57,25			15,975	5,58	13,078912	5,198	0,3766667	7,7809712	23,913239	68,30579	1,023292087
30	12	B	9	67,644	45,645	33,324501	0,469986	71,302185							9,5	13,512285	5,143	0,3641667	7,6030394	0	92,396961	1,029654946
30	12	B	10	62,848	43,122501	47,136242	0,502502	71,316963	58,71	-0,63	60,07			18,614	9,54	13,554285	5,855	0,4241667	9,3161278	29,61749	61,066382	1,165289555
30	12	B	11	57,057	42,994999	42,182034	0,464989	75,063622	68,04	-3,54	49,83			15,825	9,54	13,771209	4,945	0,3816667	8,6667718	27,735422	63,597806	1,081495548
30	12	B	12	64,435	45,350002	47,736755	0,477497	82,568291	63,72	0,28	56,67			16,718	9,6	13,831209	5,58	0,3958333	8,6598898	25,945526	65,394584	1,052915058
30	12	B	13	64,907	44,5825	37,227821	0,384998	63,808853	61,92	1,88	60,71			16,985	9,5	12,859396	5,906	0,4183333	9,0991727	26,16821	64,732617	0,863563057
30	12	B	14	60,942	44,115002	41,731651	0,417496	63,775101	65,38	2,44	64,51			16,185	9,54	12,901396	5,703	0,4308333	9,3580782	26,558039	64,083883	0,946381007
30	12	B	15	59,8	42,870007	36,176929	0,469986	63,808853	57,37	0,07	57,4		1	14,938	9,53		4,958	0,3925	8,2909699	24,979933	66,729097	1,096304929

Supplementary Table S1. Raw data of egg quality parameters with ATOL (Animal Trait Ontology for Livestock) descriptors, for hen.

				ATOL_ 0001880	ATOL_ 0002297	ATOL_ 0002119	ATOL_ 0002126	ATOL_ 0002122	ATOL_ 0002074	ATOL_ 0002073	ATOL_ 0002075	ATOL_ 0005600	ATOL_ 0005600	ATOL_ 0001883	ATOL_ 0005587	ATOL_ 0001938	ATOL_ 1881	ATOL_ 0002121	ATOL_ 1881	ATOL_ 0001883	ATOL_ 0001882	ATOL_ 0002125
**Hen age**	**Pen**	**Genotype**	**Replicate**	**Egg weight**	**Egg diameter**	**Shell strength**	**Shell fracture**	**Shell modulus**	**Yolk lightness**	**Yolk redness**	**Yolk yellow**	**Blood spot**	**Meat spot**	**Yolk weight**	**Albumen pH**	**Albumen DM**	**Shell weight**	**Shell thickness**	**Shell**	**Yolk**	**Albumen**	**Shell-to-egg-compression**
wk	no.			g	mm	N	mm	N/mm	L*	a*	b*	1=present	1=present	g	-	% (w/w)	g	mm	% (w/w)	% (w/w)	% (w/w)	%
38	1	C	1	58,155					61,53	1,81	53,57	1		15,804	9,34	12,840242	5,04	0,3766667	8,6664947	27,175651	64,157854	
38	1	C	2	63,93					58,09	3,74	52,9			18,538	9,36	12,860242	6,264	0,4358333	9,7982168	28,997341	61,204442	
38	1	C	3	59,285					65,41	-2,89	53			18,324	9,45	13,596121	5,318	0,4041667	8,9702286	30,908324	60,121447	
38	1	C	4	66,729					62,15	-1,23	50,85			16,462	9,47	13,616121	5,676	0,3958333	8,5060468	24,669934	66,82402	
38	1	C	5	63,403					62,09	-3,71	54,16		1	19,272	9,41	12,965316	6,52	0,455	10,283425	30,396038	59,320537	
38	1	C	6	66,06	45,250004	43,232925	0,412483	90,083092	67,61	-2,69	56,31			17,319	9,45	13,007316	6,328	0,4166667	9,5791705	26,217075	64,203754	0,91156456
38	1	C	7	66,861	45,585003		0,414997	33,773132	61,53	-2,33	48,65		1	16,258	9,42	13,238853	5,182	0,3566667	7,7504076	24,316119	67,933474	0,910380548
38	1	C	8	66,226	45,147503	39,179482	0,369987	82,568298	64,43	-1,02	55,84		1	18,302	9,46	13,280853	6,324	0,4183333	9,5491197	27,635672	62,815209	0,819507116
38	1	C	9	63,515	44,005001	44,734203	0,422493	56,301945	69,86	-4,62	47,28			15,466	9,37	13,571535	6,08	0,4291667	9,5725419	24,350154	66,077305	0,960102239
38	1	C	10	66,98	45,205006	44,734203	0,424999	52,548485	61,3	-2,92	52,87			17,063	9,41	13,613535	6,103	0,4133333	9,1116751	25,474769	65,413556	0,94015915
38	1	C	11	55,299	42,382504	40,080246	0,462486	52,543468	65,08	-2,92	49,44			15,549	9,43	12,215557	4,617	0,38	8,3491564	28,118049	63,532794	1,09121915
38	1	C	12	62,549	43,9575	25,96825	0,43499	48,755623	57,98	0,59	49,12		1	16,531	9,49	12,275557	4,547	0,305	7,2695007	26,42888	66,30162	0,989569471
38	1	C	13	64,958	44,160004	39,329609	0,367489	67,575218	57,74	2	55,07			18,37	9,43	12,803729	6,116	0,4291667	9,4153145	28,279812	62,304874	0,832176102
38	1	C	14	62,988	44,7925	45,48484	0,392487	78,830231	64,02	-3,11	49,97			18,929	9,47	12,845729	5,876	0,4275	9,328761	30,051756	60,619483	0,876233744
38	1	C	15	62,141	44,872501	48,487392	0,414986	60,055389	67,14	-4,1	48,41		1	17,989	9,46		5,693	0,4008333	9,1614232	28,948681	61,889896	0,924811389
38	2	D	1	60,295	38,255001	52,394535	0,387501	75,06208	61,88	-0,34	57,95			17,026	9,34	12,511266	6,163	0,43	10,221411	28,237831	61,540758	1,012942073
38	2	D	2	65,57	39,327499	50,14262	0,442501	48,790352	62,56	-2,52	50,04			17,86	9,36	12,531266	6,11	0,4258333	9,3182858	27,238066	63,443648	1,125169439
38	2	D	3	61,545	38,822498	50,593002	0,347496	78,822708	66,34	-3,49	49,38			16,369	9,33	13,563487	6,652	0,4716667	10,808352	26,596799	62,594849	0,895089234
38	2	D	4	68,917	40,130001	49,842365	0,389996	108,87117	64,72	-3,79	49,41			19,069	9,35	13,583487	6,356	0,4275	9,2226882	27,669516	63,107796	0,971831523
38	2	D	5	62,193	38,072498	45,788921	0,409996	63,808853	66,54	-2,94	47,58			21,878	9,11	12,341715	5,874	0,4308333	9,4447928	35,177592	55,377615	1,076882321
38	2	D	6	61,187	38,814999		0,154995	82,576172	66,7	-4,72	44,6			17,577	9,15	12,383715	6,269	0,4433333	10,24564	28,72669	61,027669	0,399317285
38	2	D	7	65,85	39,110001	52,244408	0,39999	71,315781	66,84	-2,93	51,78			17,772	9,38	12,981147	6,564	0,4433333	9,9681093	26,98861	63,04328	1,022730733
38	2	D	8	59,535	37,975002	47,890709	0,444984	67,562317	72,59	-5,19	46,39			17,026	9,42	13,023147	5,599	0,3941667	9,4045519	28,598304	61,997145	1,171781373
38	2	D	9	61,898	38,172501	45,488667	0,36499	71,308983	67,14	-1,67	52,87			16,379	9,37	13,21224	6,103	0,4316667	9,8597693	26,461275	63,678956	0,956159514
38	2	D	10	59,478	37,987499	50,442875	0,4175	71,315788	64,55	-1,41	51,43			17,352	9,41	13,25424	5,946	0,4208333	9,9969737	29,173812	60,829214	1,099045768
38	2	D	11	63,996	39,43	43,687134	0,397488	63,80278	65,94	-3,93	50,05		1	15,703	9,28	12,97588	5,828	0,41	9,1068192	24,537471	66,35571	1,008085214
38	2	D	12	66,058	39,657501	41,885601	0,39249	75,069244	65,07	-3,79	50,15			18,101	9,34	13,03588	5,834	0,4033333	8,8316328	27,401677	63,76669	0,989699275
38	2	D	13	62,495	38,764999	40,684582	0,359993	75,069244	68,59	-3,15	51,84			16,505	9,38	13,334482	5,868	0,4066667	9,3895512	26,410113	64,200336	0,928654738
38	2	D	14	69,159	41,762501	37,231647	0,3825	90,09169	65,67	-1,53	54,02			17,457	9,42	13,376482	6,267	0,4108333	9,0617273	25,241834	65,696439	0,915893423
38	2	D	15	72,394	41,837502	51,794025	0,457489	86,329628	70,56	-4,06	53,79			19,543	9,43		6,697	0,4216667	9,2507666	26,995331	63,753902	1,093490238
38	3	B	1	66,833	39,627499	35,12986	0,419994	63,814953	59,04	-0,39	53,83	1		18,798	9,27	13,074886	5,218	0,365	7,8075202	28,126824	64,065656	1,059854925
38	3	B	2	67,051	38,5825	28,974628	0,334991	60,049671	59,89	-0,34	58,16			19,396	9,29	13,094886	5,471	0,3875	8,1594607	28,927234	62,913305	0,868245966
38	3	B	3	68,262	39,057499	28,674374	0,344982	52,563515	65,59	-2,26	56,28		1	18,245	9,36	12,585559	5,357	0,3875	7,8477044	26,727901	65,424394	0,883267001
38	3	B	4	62,952	38,072498	36,030624	0,407486	60,049667	67,28	-3,19	54,39			16,786	9,38	12,605559	5,79	0,415	9,1974838	26,66476	64,137756	1,070289635
38	3	B	5	62,992	37,852501	42,786369	0,414989	60,049667	66,3	-4,26	50,34		1	16,349	9,38	13,251543	5,932	0,425	9,4170688	25,954089	64,628842	1,096331785
38	3	B	6	64,59	37,634998	34,52935	0,409985	45,037258	64,13	0,7	56,09	1		18,133	9,42	13,293543	5,838	0,4158333	9,0385509	28,074005	62,887444	1,089371653
38	3	B	7	66,516	39,112503	34,229095	0,36499	56,301926	59,51	-1,07	55,42			17,862	9,4	12,487834	5,89	0,3941667	8,8550123	26,853689	64,291298	0,933179858
38	3	B	8	65,671	37,962502	36,180752	0,384987	60,055389	65,32	-0,02	57,55		1	17,302	9,44	12,529834	5,639	0,4008333	8,586743	26,346485	65,066772	1,014124412
38	3	B	9	65,138	39,295002	37,381775	0,372482	60,055401	61,5	-1,09	58,98		1	18,043	9,36	12,52733	5,875	0,3983333	9,0193128	27,699653	63,281034	0,9479119
38	3	B	10	66,368	38,900002	25,821949	0,349998	41,295959	60,84	-0,79	60,06			20,137	9,4	12,56933	5,176	0,3941667	7,7989392	30,34143	61,859631	0,899737743
38	3	B	11	63,766	38,139999	46,089176	0,429985	60,055389	65,79	-4,37	52,77		1	16,854	9,39	12,627713	5,839	0,3975	9,1569175	26,431013	64,412069	1,127385976
38	3	B	12	65,653	39,369999	35,279987	0,382496	56,307301	64,33	-4,54	47,9			17,752	9,45	12,687713	5,669	0,3958333	8,634792	27,03913	64,326078	0,971541808
38	3	B	13	65,439	38,0075	43,537006	0,424995	56,301926	67,88	-1,02	56,98	1		16,731	9,32	12,867244	5,905	0,4366667	9,0236709	25,567322	65,409007	1,1181872
38	3	B	14	68,453	38,84	31,076414	0,362492	56,301926	61,81	-2,16	55,66			20,009	9,36	12,909244	5,434	0,3775	7,9382934	29,230275	62,831432	0,933295572
38	3	B	15	65,349	39,780003	40,534454	0,419994	63,7967	64,77	-4,24	44,65			18,656	9,45		5,88	0,4175	8,9978424	28,548256	62,453901	1,055791776

Supplementary Table S1. Raw data of egg quality parameters with ATOL (Animal Trait Ontology for Livestock) descriptors, for hen.

Hen age wk	Pen no.	Genotype	Replicate	ATOL_0001880 Egg weight g	ATOL_0002297 Egg diameter mm	ATOL_0002119 Shell strength N	ATOL_0002126 Shell fracture mm	ATOL_0002122 Shell modulus N/mm	ATOL_0002074 Yolk lightness L*	ATOL_0002073 Yolk redness a*	ATOL_0002075 Yolk yellow b*	ATOL_0005600 Blood spot 1=present	ATOL_0005600 Meat spot 1=present	ATOL_0001883 Yolk weight g	ATOL_0005587 Albumen pH -	ATOL_0001938 Albumen DM % (w/w)	ATOL_1881 Shell weight g	ATOL_0002121 Shell thickness mm	ATOL_1881 Shell % (w/w)	ATOL_0001883 Yolk % (w/w)	ATOL_0001882 Albumen % (w/w)	ATOL_0002125 Shell-to-egg-compression %
38	4	A	1	59,41	38,112499	41,585346	0,409992	56,296562	65,71	-1,79	55,86			16,663	9,47	13,654596	5,44	0,4025	9,1567076	28,047467	62,795826	1,075741583
38	4	A	2	63,283	37,9025	42,936497	0,389996	60,043949	64,96	-0,54	61,53			17,518	9,49	13,674596	6,534	0,4916667	10,325048	27,682	61,992952	1,02894532
38	4	A	3	64,182	37,767502	33,478458	0,359997	60,049671	65,46	3,65	60,6			17,709	9,42	14,049533	5,648	0,4075	8,7999751	27,591848	63,608177	0,953192509
38	4	A	4	62,912	37,640003	45,488667	0,394993	60,061134	67,22	-1,33	56,32			17,702	9,43	13,029638	6,505	0,4525	10,33984	28,137716	61,522444	1,049396835
38	4	A	5	58,053	35,93	32,877945	0,389999	75,047775	63,33	-1,16	56,34			16,02	9,47	13,071638	5,598	0,4158333	9,6429125	27,595473	62,761614	1,085441136
38	4	A	6	59,641	37,8475	41,134964	0,389996	63,808861	62,73	-1,5	57,79			17,438	9,47	13,26835	5,562	0,4308333	9,3257994	29,238276	61,435925	1,030440584
38	4	A	7	63,376	37,2775	40,834709	0,41	52,538445	68,7	-1,3	57,78			17,537	9,51	13,31035	6,382	0,4466667	10,070058	27,671358	62,258584	1,099859164
38	4	A	8	66,137	39,919998	41,585346	0,364994	82,576172	65,47	-0,37	55,41			17,83	9,41	14,070743	6,605	0,455	9,9868455	26,959191	63,053964	0,914313673
38	4	A	9	62,334	37,9725	43,837261	0,369987	71,315781	61,73	1,02	60,72			17,501	9,45	13,417045	6,597	0,4725	10,583309	28,07617	61,34052	0,974355125
38	4	A	10	60,281	37,385002	34,078968	0,379997	52,543461	67,92	0,96	60,34			18,058	9,49	13,459045	5,822	0,405	9,6581012	29,956371	60,385528	1,016442369
38	4	A	11	57,974	37,877502	46,839813	0,422489	71,302177	64,48	-0,7	58,01			16,855	9,44	13,572798	5,577	0,4266667	9,6198296	29,073378	61,306793	1,115408825
38	4	A	12	57,509	37,18	49,842365	0,442497	48,79966	61,62	-3,75	54,03			17,357	9,5	13,632798	5,51	0,4333333	9,581109	30,181363	60,237528	1,190147929
38	4	A	13	65,163	39,697498				55,14	-1,57	47,81			16,25	9,42	14,652615	5,949	0,4216667	9,1294139	24,937465	65,933122	
38	4	A	14	67,235	39,715	41,435219	0,389996	67,555878	66,02	1,13	60,37	1		16,48	9,38	14,007533	5,726	0,4058333	8,5163977	24,511043	66,972559	0,981986655
38	4	A	15	60,319	37,577499	56,147724	0,464985	60,055397	62,83	-0,89	52,4			15,572	9,42		6,167	0,4475	10,223976	25,816078	63,959946	1,237402734
38	5	B	1	58,081	37,552502	35,580242	0,392494	52,54847	64,84	-2,68	49,72	1		17,193	9,34	13,685937	5,282	0,4166667	9,094196	29,601763	61,304041	1,045187349
38	5	B	2	65,837	38,272503	21,768503	0,324986	41,292023	64,44	0,4	55,76			17,052	9,36	13,705937	4,71	0,3633333	7,1540319	25,90033	66,945638	0,849137042
38	5	B	3	57,999	37,52	26,722713	0,319996	56,307301	66,54	-2,76	53,6			16,128	9,36	13,152279	5,21	0,4141667	8,9829135	27,807376	63,209711	0,852867804
38	5	B	4	51,748	35,670002	24,170546	0,344994	63,802773	65,9	-3,23	57,95	1		14,993	9,38	13,172279	4,395	0,3541667	8,4930819	28,9731	62,533818	0,967182452
38	5	B	5	60,26	37,7075	42,18586	0,404991	60,055401	67,22	-4,49	50,21			16,088	9,39	13,216692	5,876	0,4308333	9,7510787	26,697644	63,551278	1,074033017
38	5	B	6	65,335	38,782501	29,274883	0,314999	71,302177	66,16	-2,68	46,04			18,271	9,43	13,258692	5,282	0,3833333	8,0844876	27,965103	63,950409	0,812219408
38	5	B	7	61,285					64,52	-0,64	49,64			16,813	9,34	13,726297	4,808	0,3633333	7,8453129	27,434119	64,720568	
38	5	B	8	68,895	40,654999	23,870289	0,277496	90,083092	65,88	-4,19	49,9			18,365	9,38	13,768297	6,135	0,4325	8,9048552	26,656506	64,438639	0,682563047
38	5	B	9	65,313	38,695	36,631138	0,362488	56,301926	65,73	-2,03	52,71			17,387	9,4	13,145667	5,668	0,3933333	8,6782111	26,62104	64,700749	0,93678253
38	5	B	10	69,664	40,455002	36,180752	0,37999	86,33786	64,95	1,64	59,22			17,831	9,44	13,187667	6,276	0,4158333	9,0089573	25,595717	65,395326	0,939290523
38	5	B	11	70,052	40,705002	35,880497	0,392487	86,337868	66,19	-3,83	49,62	1		18,94	9,32	13,004188	6,197	0,4175	8,8462856	27,037058	64,116656	0,964223021
38	5	B	12	61,904	37,8825	37,531902	0,4175	56,307301	62,13	-2,46	52,9	1		17,221	9,38	13,064188	5,342	0,3975	8,6294908	27,818881	63,551628	1,102091995
38	5	B	13	62,055	38,607502	28,524246	0,342495	52,548481	67,23	0,87	58,07			16,258	9,31	14,125801	4,572	0,3608333	7,3676577	26,199339	66,433003	0,887120332
38	5	B	14	64,406	38,615002	33,028072	0,384998	52,54847	65,8	-2,15	55,38			17,104	9,35	14,167801	5,62	0,3983333	8,7258951	26,556532	64,717573	0,997016652
38	5	B	15	69,036	40,700001	22,218885	0,502502	63,808853	66,43	-2,67	49,72			19,241	9,43		5,417	0,375	7,8466307	27,870966	64,282403	1,234648618
38	6	C	1	60,089	39,3675	44,437771	0,414997	56,296562	59,45	-4,25	44,36	1		14,908	9,29	13,499434	5,641	0,4025	9,3877415	24,809865	65,802393	1,054161428
38	6	C	2	63,037	38,705002	31,526798	0,372486	56,296574	66,65	-3,19	52,77	1	1	16,335	9,31	13,519434	5,297	0,3758333	8,4030014	25,913352	65,683646	0,962371737
38	6	C	3	59,361	37,515003	40,2342	0,470001	37,534622	59,15	-3,8	38,46			17,093	9,3	13,663089	5,026	0,3758333	8,4668385	28,795	62,738161	1,252834766
38	6	C	4	59,545	38,1875	39,183308	0,382496	71,315781	68,02	-3,33	54,01			16,166	9,32	13,683089	5,306	0,3983333	8,9109077	27,149215	63,939877	1,001626187
38	6	C	5	65,985	39,32	38,132412	0,362492	63,821037	64,94	-0,17	58,89			16,54	9,36	13,312333	6,084	0,41	9,2202773	25,066303	65,71342	0,92190234
38	6	C	6	64,116	38,887501				64,05	-0,51	55,16			16,232	9,4	13,354333	6,046	0,405	9,4297835	25,316614	65,253603	
38	6	C	7	57,655	38,355				64,8	-3,2	55,56			16,325	9,4	13,502615	4,089	0,3241667	7,0921863	28,314977	64,592837	
38	6	C	8	63,308	37,794998	28,374119	0,379986	48,79501	52,55	-2,89	44,76				9,44	13,544615	5,022	0,3608333	7,9326467			1,005387009
38	6	C	9	62,001	39,607498	34,229095	0,359997	60,055401	66,22	-4,71	50,79			13,924	9,39	13,419197	5,666	0,3983333	9,1385623	22,457702	68,403735	0,908911237
38	6	C	10	63,803	39,737499	42,335987	0,397491	60,055401	64,71	-0,86	53,02			17,974	9,43	13,461197	5,751	0,415	9,0136827	28,171089	62,815228	1,000291941
38	6	C	11	61,793	37,752502	30,626032	0,347488	56,291195	59,65	-1,43	54,14			17,736	9,43	13,236332	5,403	0,3933333	8,7437088	28,70228	62,554011	0,920437008
38	6	C	12	55,568	36,870003	40,684582	0,394993	60,049667	63,18	-1,42	58,62		1	16,378	9,49	13,296332	5,199	0,3866667	9,3561042	29,473798	61,170098	1,071312633
38	6	C	13	64,199	39,022499	35,880497	0,407497	60,055397	61,41	2,12	56,09	1		17,07	9,41	13,611061	5,274	0,3733333	8,2150812	26,589199	65,19572	1,044261671
38	6	C	14	61,644	38,017502	25,671822	0,374992	30,030567	65,38	-2,95	52,38		1	18,551	9,45	13,653061	5,111	0,3641667	8,2911557	30,093764	61,61508	0,986366753
38	6	C	15	57,417	37,365005	44,137516	0,442486	60,061119	57,86	-1,31	56,45			16,807	9,44		5,321	0,4016667	9,2672902	29,271818	61,460891	1,184225721

Supplementary Table S1. Raw data of egg quality parameters with ATOL (Animal Trait Ontology for Livestock) descriptors, for hen.

				ATOL_0001880	ATOL_0002297	ATOL_0002119	ATOL_0002126	ATOL_0002122	ATOL_0002074	ATOL_0002073	ATOL_0002075	ATOL_0005600	ATOL_0005600	ATOL_0001883	ATOL_0005587	ATOL_0001938	ATOL_1881	ATOL_0002121	ATOL_1881	ATOL_0001883	ATOL_0001882	ATOL_0002125
**Hen age**	**Pen**	**Genotype**	**Replicate**	**Egg weight**	**Egg diameter**	**Shell strength**	**Shell fracture**	**Shell modulus**	**Yolk lightness**	**Yolk redness**	**Yolk yellow**	**Blood spot**	**Meat spot**	**Yolk weight**	**Albumen pH**	**Albumen DM**	**Shell weight**	**Shell thickness**	**Shell**	**Yolk**	**Albumen**	**Shell-to-egg-compression**
wk	no.			g	mm	N	mm	N/mm	L*	a*	b*	1=present	1=present	g	-	% (w/w)	g	mm	% (w/w)	% (w/w)	% (w/w)	%
38	7	A	1	59,545	37,702499	48,491219	0,409985	67,575211	66,03	0,55	59,53			14,93	9,45	13,997516	6,1	0,4333333	10,244353	25,073474	64,682173	1,087421287
38	7	A	2	61,684	37,8475	35,12986	0,349998	63,802773	58,03	0,7	53,69		1	19,501	9,47	14,017516	5,745	0,3658333	9,3135983	31,614357	59,072045	0,924758571
38	7	A	3	63,36	39,357498	46,239304	0,417496	56,296574	67,83	-2,37	55,74			18,363	9,35	13,906743	5,474	0,4125	8,6395202	28,982008	62,378472	1,060778813
38	7	A	4	62,711	38,467499	33,778713	0,414997	60,055401	63,19	-2,46	55,89			18,215	9,37	13,926743	5,188	0,3825	8,2728708	29,045941	62,681188	1,07882501
38	7	A	5	60,742	37,482498	43,386879	0,404984	56,307301	64,04	1,33	56,6			17,734	9,42	13,74176	6,021	0,4483333	9,9124164	29,195614	60,891969	1,080461606
38	7	A	6	61,524	37,654999	43,837261	0,397488	63,814953	59,72	-0,78	52,97			16,695	9,46	13,78376	6,906	0,4358333	11,224888	27,135752	61,63936	1,055604861
38	7	A	7	64,579	38,189999	32,427563	0,332493	52,54847	65,23	-1,5	59			18,455	9,37	13,096871	6,012	0,4166667	9,3095279	28,577401	62,113071	0,87062846
38	7	A	8	63,022	38,077499	43,987389	0,369987	75,069244	63,29	2,22	54,5		1	18,384	9,41	13,138871	6,09	0,4441667	9,6632922	29,170766	61,165942	0,971668334
38	7	A	9	66,266	38,759998	33,628586	0,382484	52,54847	68,79	-0,87	61,73			19,544	9,28	13,078257	5,193	0,3875	7,836598	29,493254	62,670148	0,986800877
38	7	A	10	61,318	37,247501	45,038284	0,432484	52,558491	61,26	1,45	53,21			17,437	9,32	13,120257	5,987	0,4441667	9,763854	28,437001	61,799145	1,161108768
38	7	A	11	61,535	38,080002	57,19862	0,4375	75,069244	59,59	1,6	67,75			16,242	9,35	13,229669	6,088	0,4358333	9,8935565	26,394735	63,711709	1,148896998
38	7	A	12	64,318	38,360001	40,534454	0,402489	60,049671	66,54	-2,18	58,35			18,823	9,41	13,289669	6,085	0,4116667	9,4608041	29,265524	61,273671	1,04924137
38	7	A	13	54,508	36,125	42,936497	0,449993	86,337875	56,49	5,92	66,45			15,832	9,32	13,53886	5,005	0,3816667	9,1821384	29,045278	61,772584	1,245655363
38	7	A	14	63,842	38,5825	41,735474	0,36499	63,814938	68,2	-2,04	58,33			17,132	9,36	13,58086	6,068	0,43	9,5047148	26,834999	63,660286	0,945998834
38	7	A	15	63,385	37,877499	38,432667	0,352497	63,814945	60,73	1,22	62,05			18,576	9,36		6,184	0,4525	9,7562515	29,306618	60,93713	0,930623746
38	8	D	1	59,174	38,095001	44,137516	0,347485	82,576172	61,09	-0,42	60,07			15,692	9,39	13,110932	5,798	0,4375	9,7982222	26,518403	63,683374	0,912153802
38	8	D	2	65,082	39,645	53,2953	0,434982	60,049671	69,6	0,13	61,92			17,195	9,41	13,130932	6,262	0,4391667	9,621708	26,420516	63,957776	1,097192584
38	8	D	3	66,855	39,540001	46,539558	0,419994	60,061134	65,36	-4,12	53,81			16,852	9,32	13,642656	6,064	0,415	9,0703762	25,206791	65,722833	1,062200277
38	8	D	4	56,56	37,727501	41,585346	0,364998	75,076408	70,09	-3,77	49,18			16,947	9,34	13,662656	5,297	0,4058333	9,3652758	29,962871	60,671853	0,967458725
38	8	D	5	65,054	39,200001	37,832157	0,349983	60,061119	63,93	-3,05	51,64			17,198	9,38	13,242889	6,106	0,4158333	9,3860485	26,436499	64,177453	0,892813753
38	8	D	6	56,807	37,560001	45,939049	0,42749	71,308975	65,05	-2,87	54,99			14,498	9,42	13,284889	5,583	0,42	9,8280142	25,521503	64,650483	1,138152259
38	8	D	7	58,976	38,067501	41,735474	0,399994	67,568764	66,43	-0,97	57,79			14,36	9,35	13,718021	5,435	0,3975	9,2156131	24,348888	66,435499	1,050749299
38	8	D	8	63,279	38,0275	49,391983	0,394985	71,315781	66,18	-1,93	55,91			17,622	9,39	13,760021	6,491	0,46	10,257747	27,848101	61,894151	1,038682532
38	8	D	9	63,758	39,265003	58,549767	0,379993	97,580711	53,13	1,28	52			17,405	9,34	13,431188	6,713	0,4625	10,528875	27,298535	62,17259	0,967765111
38	8	D	10	60,43	38,215	45,338539	0,379997	63,802773	62,64	-0,04	49,75			14,94	9,38	13,473188	5,702	0,4516667	9,4357107	24,72282	65,841469	0,994366087
38	8	D	11	60,527	38,352501	38,132412	0,359993	71,322594	58,52	-0,25	58,49			16,298	9,36	13,157991	5,757	0,43	9,5114577	26,926826	63,561716	0,938642828
38	8	D	12	58,47	38,0975	47,290195	0,362499	78,822708	63,04	-4,65	47,79			18,196	9,42	13,217991	5,82	0,4291667	9,9538225	31,120233	58,925945	0,951503379
38	8	D	13	55,045	37,287498	45,188412	0,417496	56,301926	65,33	-3,95	50,26			16,309	9,32	12,687205	5,46	0,4425	9,9191571	29,628486	60,452357	1,119667509
38	8	D	14	60,601	37,294998	43,086624	0,412487	60,049667	64,29	-0,28	51,53			13,508	9,36	12,729205	6,079	0,4416667	10,031188	22,290061	67,678751	1,106011589
38	8	D	15	56,539	37,564999	53,145172	0,419991	67,575211	65,46	-2,8	53,5			14,916	9,41		5,775	0,4466667	10,214188	26,38179	63,404022	1,118038097
38	9	D	1	61,334	37,947502	44,137516	0,37999	78,830231	63,11	1,09	54,5			14,306	9,26	12,353636	5,922	0,4291667	9,6553298	23,324746	67,019924	1,001357085
38	9	D	2	63,953	38,852501	28,974628	0,264992	67,562325	62,46	-3,17	53,76			16,637	9,28	12,373636	6,383	0,455	9,9807671	26,014417	64,004816	0,682046183
38	9	D	3	59,738	38,422501	36,48101	0,380001	56,296562	67,78	-2,38	50,18			15,059	9,25	14,213386	4,975	0,38	8,3280324	25,20841	66,463558	0,989006416
38	9	D	4	61,279	39,407501	39,483562	0,367493	63,7967	48,62	4,53	47,87			13,782	9,27	14,233386	5,457	0,4041667	8,9051714	22,490576	68,604253	0,932545812
38	9	D	5	60,962	38,66				58,1	-1,95	53,72			14,732	9,32	14,710544	5,1	0,3766667	8,3658673	24,165874	67,468259	
38	9	D	6	58,154	38,047501	45,038284	0,357494	86,313164	67,41	1,81	58,16			13,958	9,36	14,752544	5,396	0,4116667	9,2788114	24,001788	66,7194	0,939599161
38	9	D	7	59,328	38,037498	42,18586	0,389988	67,568764	68	-1,8	56,39			15,933	9,27	13,314783	5,61	0,4316667	9,4559061	26,855785	63,688309	1,025272482
38	9	D	8	60,72	38,3825	46,239304	0,404987	67,568764	64,38	-2,03	48,94			15,688	9,31	13,356783	5,542	0,415	9,127141	25,836627	65,036232	1,055134501
38	9	D	9	65,019	39,425003	52,244408	0,432487	56,301926	63,6	-1,42	60,77			15,226	9,36	13,435917	6,406	0,445	9,8525047	23,41777	66,729725	1,0969866
38	9	D	10	65,535	38,857502	41,585346	0,357491	67,555885	68,17	-2,08	56,07			15,412	9,4	13,477917	6,428	0,4466667	9,8084993	23,517205	66,674296	0,9200051
38	9	D	11	58,119	37,755001		0,44249	71,30899	66,36	-2,38	52,25			15,651	9,36	12,542799	5,78	0,4316667	9,9451126	26,929231	63,125656	1,172003677
38	9	D	12	60,557	37,737503	45,488667	0,374985	82,576164	65,68	-1,31	56,63			17,23	9,42	12,602799	5,93	0,4375	9,792427	28,452532	61,755041	0,993666698
38	9	D	13	65,552	38,990002	46,839813	0,367489	78,815193	66,21	-2,04	50,19		1	15,847	9,13	12,943286	6,494	0,45	9,906639	24,174701	65,91866	0,942521111
38	9	D	14	55,634	38,690002				61,74	-1,15	55,38			16,094	9,17	12,985286	3,179	0,27	5,7141316	28,928353	65,357515	
38	9	D	15	65,539	40,305	49,391983	0,4375	105,10697	60,89	3,94	60,21			16,295	9,21		6,94	0,4466667	10,589115	24,863059	64,547826	1,085473266

Supplementary Table S1. Raw data of egg quality parameters with ATOL (Animal Trait Ontology for Livestock) descriptors, for hen.

**Hen age**<br>wk	**Pen**<br>no.	**Genotype**	**Replicate**	ATOL_0001880<br>**Egg weight**<br>g	ATOL_0002297<br>**Egg diameter**<br>mm	ATOL_0002119<br>**Shell strength**<br>N	ATOL_0002126<br>**Shell fracture**<br>mm	ATOL_0002122<br>**Shell modulus**<br>N/mm	ATOL_0002074<br>**Yolk lightness**<br>L*	ATOL_0002073<br>**Yolk redness**<br>a*	ATOL_0002075<br>**Yolk yellow**<br>b*	ATOL_0005600<br>**Blood spot**<br>1=present	ATOL_0005600<br>**Meat spot**<br>1=present	ATOL_0001883<br>**Yolk weight**<br>g	ATOL_0005587<br>**Albumen pH**<br>-	ATOL_0001938<br>**Albumen DM**<br>% (w/w)	ATOL_1881<br>**Shell weight**<br>g	ATOL_0002121<br>**Shell thickness**<br>mm	ATOL_1881<br>**Shell**<br>% (w/w)	ATOL_0001883<br>**Yolk**<br>% (w/w)	ATOL_0001882<br>**Albumen**<br>% (w/w)	ATOL_0002125<br>**Shell-to-egg-compression**<br>%
38	11	C	1	66,986	39,919998	37,08152	0,377499	78,807678	68,06	-2,15	51,94			16,06	9,35	14,250122	5,584	0,4033333	8,3360702	23,975159	67,688771	0,945638825
38	11	C	2	64,485	37,352501	35,73037	0,349998	67,542992	62,31	-2,24	54,92			18,392	9,37	14,270122	6,213	0,4291667	9,6347988	28,521362	61,84384	0,937013562
38	11	C	3	61,642	38,7925	45,488667	0,397484	60,049667	66,56	-3,88	49,1		1	15,969	9,38	12,729097	5,499	0,405	8,9208656	25,906038	65,173096	1,024641361
38	11	C	4	64,767	39,192501	40,834709	0,429989	48,790356	67,77	-1,37	60,99			18,063	9,4	12,749097	5,357	0,3833333	8,2711875	27,889203	63,83961	1,097120595
38	11	C	5	62,761	39,137501	41,585346	0,359997	71,315781	59,81	0,59	57,34		1	16,268	9,38	13,853539	5,973	0,4291667	9,5170568	25,920556	64,562387	0,91982623
38	11	C	6	60,743	38,477501	41,735474	0,332489	71,308975	66,41	0,89	58,69			17,617	9,42	13,895539	5,887	0,4366667	9,6916517	29,002519	61,305829	0,864112771
38	11	C	7	62,48	37,82	34,829605	0,357498	67,562325	61,22	-4,71	42,22			17,976	9,42	12,282665	5,723	0,4016667	9,1597311	28,770807	62,069462	0,945261766
38	11	C	8	65,935	38,2575	27,173098	0,302494	71,308983	67	-3,51	54,2			17,018	9,46	12,324665	6,013	0,4066667	9,1195875	25,810268	65,070145	0,790678952
38	11	C	9	62,696	38,77	36,631138	0,36499	67,555885	62,88	-0,62	57,02			17,662	9,41	12,026858	5,481	0,3833333	8,7421845	28,170856	63,086959	0,941423781
38	11	C	10	66,538	37,9925	25,671822	0,299992	60,061111	64,71	-3,73	50,56			16,961	9,45	12,068858	6,038	0,4008333	9,0745138	25,490697	65,434789	0,789608475
38	11	C	11	60,972	37,622501	37,68203	0,372498	56,285831	63,65	1,08	56,83			17,196	9,29	12,746413	5,877	0,445	9,6388506	28,20311	62,15804	0,990093668
38	11	C	12	60,578	38,029999	33,778713	0,324993	78,807678	60,7	3,14	54,59			15,722	9,35	12,806413	5,233	0,3783333	8,6384496	25,953316	65,408234	0,854570099
38	11	C	13	62,825	37,5975	34,229095	0,380001	60,049671	67,82	-5,13	51,7			18,482	9,4	13,181147	5,669	0,3966667	9,0234779	29,418225	61,558297	1,010708159
38	11	C	14	62,228	39,717499	37,832157	0,407486	67,549431	64,74	-4,16	41,01			18,013	9,44	13,223147	5,177	0,375	8,3194061	28,946776	62,733818	1,025960874
38	11	C	15	65,372	39,052502	33,928841	0,359997	60,055397	66,38	-3,45	51,62			17,133	9,45		5,569	0,3858333	8,5189378	26,208468	65,272594	0,921828261
38	12	B	1	64,683	38,595001				63,05	-0,24	54,85	1		17,009	9,43	12,791932	5,991	0,4166667	9,2620936	26,295936	64,441971	
38	12	B	2	62,455	38,810001	43,837261	0,384995	75,069244	66,73	-1,55	57,69		1	16,16	9,45	12,811932	5,788	0,4141667	9,2674726	25,87463	64,857898	0,991999459
38	12	B	3	65,489	38,150002	34,078968	0,347496	67,555885	61,8	-0,11	56,94	1	1	16,817	9,43	13,109195	5,877	0,4158333	8,9740262	25,679122	65,346852	0,91086758
38	12	B	4	61,886	38,092503	33,478458	0,319996	75,062088	67,74	-0,53	56,6		1	16,673	9,45	13,129195	5,483	0,3933333	8,8598391	26,941473	64,198688	0,840049812
38	12	B	5	66,852	39,794998	31,97718	0,394989	67,562325	64,87	-0,3	57,97	1		18,274	9,4	13,583186	5,274	0,3716667	7,8890684	27,335009	64,775923	0,992559417
38	12	B	6	62,746	37,422501	34,52935	0,404991	48,79501	65,17	-2,56	53,47	1		19,194	9,44	13,625186	5,553	0,4025	8,8499665	30,589998	60,560036	1,082212544
38	12	B	7	63,228	37,215	31,376671	0,39249	48,790356	57,16	-1	63,12			19,271	9,41	12,59224	5,528	0,385	8,742962	30,478585	60,778453	1,054655381
38	12	B	8	67,154	39,137501	30,926287	0,322498	67,562325	66,1	-3,26	54,9			17,704	9,45	12,63424	5,819	0,4191667	8,6651577	26,363284	64,971558	0,824012754
38	12	B	9	64,279	37,775002	25,972076	0,372486	52,54847	62,82	1	55,9	1		15,751	9,26	12,7344	4,555	0,355	7,0862957	24,504115	68,409589	0,986064806
38	12	B	10	66,921	39,625	27,473352	0,357494	56,301926	64,84	-0,08	58,06			17,713	9,3	12,7764	5,162	0,3616667	7,7135727	26,468523	65,817905	0,90219306
38	12	B	11	67,634	38,294998	37,68203	0,367485	56,307308				1		16,257	9,42	12,613678	6,136	0,4266667	9,0723601	24,036727	66,890913	0,959616188
38	12	B	12	58,051	37,912498	45,488667	0,397491	71,322586	55,74	-1,77	40,02			16,201	9,48	12,673678	5,539	0,4166667	9,54161	27,908219	62,550171	1,048443181
38	12	B	13	64,455	38,877499	22,969524	0,324997	52,543457	67,07	-2,18	54,77		1	17,355	9,4	13,143629	5,026	0,3791667	7,7976883	26,925762	65,27655	0,835951407
38	12	B	14	62,962	38,700001	33,328327	0,334995	71,315781	64,91	-0,22	58,52			16,881	9,44	13,185629	5,444	0,3866667	8,6464852	26,81141	64,542105	0,865620133
38	12	B	15	63,331	38,5975	24,921183	0,317482	56,291195	66,29	-0,01	60,79			17,746	9,42		4,954	0,3716667	7,8223935	28,021032	64,156574	0,822545502
46	1	C	1	62,828	44,404999	51,039112	0,439991	71,322586	48,99	1,9	46,08			17,467	9,29	12,579132	6,196	0,435	9,861845	27,801299	62,336856	0,99085916
46	1	C	2	68,112	45,109997	36,927116	0,379993	56,301926	57,44	-3,03	40,34			18,316	9,31	12,599132	6,122	0,4091667	8,9881372	26,891003	64,12086	0,842369819
46	1	C	3	64,427	44,34	40,680305	0,437496	52,553486	49,96	-1,34	54,64			17,537	9,35	13,058783	5,416	0,4	8,4064135	27,219954	64,373632	0,986684709
46	1	C	4	67,984	46,260002	20,413078	0,247498	97,580704	65,47	-3,04	55,66			17,665	9,37	13,078783	5,795	0,415	8,5240645	25,984055	65,49188	0,535015109
46	1	C	5	66,665	46,125	44,883881	0,394993	108,8504	65,26	-4,63	44,07			16,835	9,19	12,350134	6,422	0,4358333	9,6332408	25,253131	65,113628	0,856353388
46	1	C	6	61,29	43,164997	45,784645	0,377483	97,580711	58,52	-2,56	54,71			16,231	9,23	12,392134	6,084	0,4266667	9,9265786	26,482297	63,591124	0,874511818
46	1	C	7	67,397	44,259998	41,58107	0,392494	60,038509	62,41	-3,54	49,01	1		16,539	9,41	12,83092	6,677	0,4483333	9,9069692	24,539668	65,553363	0,886791726
46	1	C	8	66,346	44,947498	44,883881	0,409985	60,055401	56,54	-3,08	52,16		1	17,955	9,45	12,87292	5,986	0,4166667	9,0223977	27,062671	63,914931	0,912141984
46	1	C	9	67,664	44,904999	49,237579	0,392498	71,322586	60,32	-2,2	55,25			19,482	9,37	12,475417	6,732	0,4375	9,9491606	28,792268	61,258572	0,874063041
46	1	C	10	65,854	44,682499	57,494598	0,404984	90,083092	57,85	-4,64	45,81			16,775	9,41	12,517417	7,22	0,4875	10,963647	25,473016	63,563337	0,906359333
46	1	C	11	69,148	45,989998	39,479286	0,379986	112,59315	64,74	-2,87	50,7			17,859	9,24	13,211447	5,793	0,3841667	8,3776827	25,827211	65,795106	0,826236174
46	1	C	12	64,632	44,995003	43,682858	0,402489	75,076408	60,2	-0,41	51,98			16,281	9,3	13,271447	5,896	0,415	9,1224161	25,190308	65,687276	0,894519331
46	1	C	13	62,771	43,947498	43,682858	0,397495	56,290943	63,13	-1,01	56,03			17,098	9,39	13,442489	6,042	0,4241667	9,625464	27,238693	63,135843	0,904476974
46	1	C	14	67,204	45,674999	44,583622	0,392494	105,08691	64,43	-3,09	50,51			19,262	9,43	13,484489	6,471	0,4433333	9,6288911	28,661984	61,709124	0,859319121
46	1	C	15	64,308	44,342499	43,983112	0,389999	75,072304	63,3	-3,38	51,35			15,725	9,34		6,354	0,4175	9,8805747	24,452634	65,666791	0,879515158

Supplementary Table S1. Raw data of egg quality parameters with ATOL (Animal Trait Ontology for Livestock) descriptors, for hen.

				ATOL_ 0001880	ATOL_ 0002297	ATOL_ 0002119	ATOL_ 0002126	ATOL_ 0002122	ATOL_ 0002074	ATOL_ 0002073	ATOL_ 0002075	ATOL_ 0005600	ATOL_ 0005600	ATOL_ 0001883	ATOL_ 0005587	ATOL_ 0001938	ATOL_ 1881	ATOL_ 0002121	ATOL_ 1881	ATOL_ 0001883	ATOL_ 0001882	ATOL_ 0002125
**Hen age**	**Pen**	**Genotype**	**Replicate**	**Egg weight**	**Egg diameter**	**Shell strength**	**Shell fracture**	**Shell modulus**	**Yolk lightness**	**Yolk redness**	**Yolk yellow**	**Blood spot**	**Meat spot**	**Yolk weight**	**Albumen pH**	**Albumen DM**	**Shell weight**	**Shell thickness**	**Shell**	**Yolk**	**Albumen**	**Shell-to-egg-compression**
wk	no.			g	mm	N	mm	N/mm	L*	a*	b*	1=present	1=present	g	-	% (w/w)	g	mm	% (w/w)	% (w/w)	% (w/w)	%
46	2	D	1	65,392	44,509998	48,486942	0,424992	71,315781	61,23	-1,94	52,8			17,416	9,33	12,894751	6,15	0,4225	9,4048202	26,633227	63,961953	0,954823678
46	2	D	2	66,072	44,732498	57,494598	0,449997	75,069244	62,72	-2,66	49,97			18,647	9,35	12,914751	6,447	0,4375	9,7575372	28,222242	62,02022	1,005973331
46	2	D	3	60,559	43,977497	53,891537	0,439995	71,329391	60,84	-4,17	50,56			17,179	9,3	12,613646	5,969	0,4433333	9,8565036	28,367377	61,776119	1,000500324
46	2	D	4	62,1232	44,424999	54,792301	0,424992	67,568764	57,89	-0,88	54,4			16,823	9,32	12,633646	6,424	0,445	10,340742	27,08006	62,579197	0,956650556
46	2	D	5	60,785	43,619999	43,983112	0,394989	78,817093	55,97	0,15	52,44			19,025	9,26	12,713289	5,016	0,425	8,2520359	31,29884	60,449124	0,905522717
46	2	D	6	63,42	45,055004	47,285919	0,409988	67,562317	66,89	-4,18	48,7			16,987	9,3	12,755289	5,704	0,4216667	8,9940082	26,784926	64,221066	0,909972175
46	2	D	7	61,7	43,719997	54,792301	0,404984	86,321396	65,14	-0,11	52,57			16,54	9,34	12,571776	6,682	0,4591667	10,829822	26,807131	62,363047	0,926312964
46	2	D	8	60,449	43,712498	47,285919	0,389992	78,815186	63,11	-1,71	52,95	1		18,007	9,38	12,613776	5,979	0,42	9,8909825	29,788748	60,32027	0,892175048
46	2	D	9	60,938	44,384998	51,789749	0,414989	63,802765	63,48	-1,69	53,66			18,66	9,31	12,314025	5,821	0,4158333	9,5523319	30,621287	59,826381	0,934975822
46	2	D	10	61,178	43,549999	49,838089	0,397495	86,321404	66,84	-1,74	57,87			16,933	9,35	12,356025	5,807	0,4141667	9,4919742	27,67825	62,829775	0,912732512
46	2	D	11	63,995	44,355	42,782093	0,334991	71,308983	62,62	-1,66	50,73			18,201	9,37	10,530345	6,197	0,4408333	9,683569	28,441284	61,875146	0,75524969
46	2	D	12	66,743	46,079998	54,341919	0,467487	112,6146	64,02	-3,11	48,69			16,999	9,43	10,590345	6,189	0,4383333	9,2728825	25,469338	65,25778	1,014511763
46	2	D	13	61,83	43,294998	45,634518	0,369991	86,329628	61,35	-0,38	56,16			17,141	9,39	12,802529	5,923	0,4275	9,5794922	27,722788	62,69772	0,8545814
46	2	D	14	64,196	44,155003	52,690514	0,407482	86,329628	64,77	-2,64	51,38			18,857	9,43	12,844529	6,438	0,4508333	10,028662	29,374104	60,597233	0,922844462
46	2	D	15	60,955	44,68	34,975456	0,377499	63,808853	59,42	-1,04	49,25			17,064	9,31		5,075	0,3641667	8,3258141	27,994422	63,679764	0,844894808
46	3	B	1	64,128	44,037498	48,03656	0,364986	75,054939	58,65	-3,05	53,1		1	18,115	9,25	13,216601	6,462	0,4525	10,076722	28,248191	61,675087	0,828807304
46	3	B	2	62,84	43,775002	43,082348	0,404995	75,050827	65,99	-4,1	51,11		1	15,765	9,27	13,236601	5,726	0,4033333	9,1120306	25,087524	65,800446	0,925174144
46	3	B	3	62,304	43,314999	39,479286	0,462486	60,021442	52,39	1,35	57,21			18,706	9,18	12,457532	5,513	0,3983333	8,848549	30,023754	61,127696	1,06772714
46	3	B	4	68,45	44,497498	41,280815	0,364986	71,315781	65,01	-2,78	51,46			18,211	9,2	12,477532	6,352	0,4258333	9,2797663	26,604821	64,115413	0,820239376
46	3	B	5	68,636	44,707497	37,077244	0,429985	52,543461	66,88	-2,32	50,3	1	1	18,533	9,35	12,965381	6,121	0,42	8,9180605	27,001865	64,080075	0,961773816
46	3	B	6	68,232	44,067497	33,0238	0,419983	56,262291	63,49	-4,75	48,48	1		18,529	9,39	13,007381	6,479	0,4341667	9,4955446	27,15588	63,348575	0,953044826
46	3	B	7	67,437	44,7925	47,135792	0,472496	52,538452	63,72	-1,39	57,38	1		18,147	9,34	12,890025	5,755	0,4241667	8,5338909	26,90956	64,556549	1,054855165
46	3	B	8	67,529	45,5425	35,425838	0,43499	75,054924	65,77	-6,01	50,66	1		19,253	9,38	12,932025	5,412	0,3891667	8,0143346	28,510714	63,474952	0,955129824
46	3	B	9	61,741	43,572498	41,130688	0,422493	67,568764	66,51	-3,6	51,84	1		17,79	9,29	12,585959	7,225	0,4908333	11,70211	28,813916	59,483973	0,969632267
46	3	B	10	64,497	44,482502	31,672649	0,457493	67,49881	67,24	-6,2	44,73		1	19,109	9,33	12,627959	4,981	0,3658333	7,7228398	29,627735	62,649426	1,028478569
46	3	B	11	68,746	45,032497	48,186687	0,459988	86,281593	58,09	-1,12	52,81			17,533	9,29	12,713764	6,561	0,44	9,543828	25,504029	64,952143	1,021457904
46	3	B	12	68,034	45,889999	31,372395	0,314987	112,59263	66,09	-2,18	53,4			20,029	9,35	12,773764	6,09	0,4058333	8,9514066	29,439692	61,608901	0,686395744
46	3	B	13	62,537	44,192497	46,835537	0,444992	52,543461	52,64	1,73	64,62	1		18,471	9,29	13,256826	5,893	0,4233333	9,4232215	29,536115	61,040664	1,00694016
46	3	B	14	67,476	44,174999	30,92201	0,377487	60,032784	66,41	-4,59	49,66		1	17,691	9,33	13,298826	5,36	0,38	7,9435651	26,218211	65,838224	0,854526335
46	3	B	15	61,51	43,877499	42,782093	0,414993	60,049767	64,54	-3,26	51,37		1	15,991	9,35		5,73	0,4191667	9,3155584	25,997399	64,687043	0,945799121
46	4	A	1	64,692	43,752499	55,843193	0,469994	71,30899	63,62	-1,23	52			20,141	9,36	13,241757	6,654	0,45	10,285661	31,13368	58,580659	1,074210641
46	4	A	2	62,786	43,364998	42,93222	0,434986	71,265144	54,81	-1,4	54,72		1	17,56	9,38	13,261757	6,05	0,4175	9,6359061	27,968018	62,396076	1,003080872
46	4	A	3	65,442	44,114998	43,232475	0,415001	56,27969	66,41	-1,37	57,6			19,983	9,36	13,852506	6,237	0,4308333	9,5305767	30,535436	59,933987	0,94072542
46	4	A	4	67,338	44,285	40,680305	0,442497	56,285057	64,89	-1,8	55,39			20,805	9,38	13,872506	5,765	0,3891667	8,5612878	30,896374	60,542339	0,99920289
46	4	A	5	62,007	43,93	43,382603	0,412502	52,54847	58,01	0,17	57,78			18,949	9,41	13,218142	5,715	0,4033333	9,2167013	30,559453	60,223846	0,938998407
46	4	A	6	65,47	44,294998	44,883881	0,384995	60,043945	59,2	-0,98	56,87			19,444	9,45	13,260142	6,437	0,4333333	9,8319841	29,699099	60,468917	0,869161344
46	4	A	7	64,733	44,407497	46,385155	0,465	67,557518	61,52	-2,57	50,73			18,617	9,42	12,350907	6,033	0,425	9,3198214	28,759674	61,920504	1,04712049
46	4	A	8	66,702	45,350002	53,44138	0,467499	101,34347						16,903	9,35	13,245592	6,443	0,4475	9,6593805	25,341069	64,99955	1,030868753
46	4	A	9	65,048	44,93	55,693291	0,474995	60,049667	60,39	-0,66	53,09			16,735	9,26		6,34	0,4358333	9,7466486	25,727155	64,526196	1,057188961
46	4	A	10	64,281	44,442501	45,034233	0,377499	71,308983	62,23	-0,23	53,66			19,095	9,39	13,494819	6,94	0,4433333	10,796347	29,705512	59,498141	0,849409893
46	4	A	11	62,799	42,969997	43,832985	0,434982	71,315781	63,86	-1,56	51,81			17,54	9,53	12,858838	6,387	0,44	10,170544	27,930381	61,899075	1,012292368
46	4	A	12	65,421	44,497501	53,741409	0,404999	71,308975	66,39	-1,78	56,13			19,925	9,56	12,737868	6,568	0,4483333	10,03959	30,456581	59,503829	0,910161225
46	4	A	13	64,637	44,099998	54,642174	0,445	67,534187	56,76	-0,54	62,29			19,766	9,34	13,174147	6,766	0,46	10,467689	30,580008	58,952303	1,009070341
46	4	A	14	68,837	45,07	50,588726	0,467499	52,54847	57,42	-0,18	61,59			19,703	9,38	13,216147	6,457	0,4441667	9,3801299	28,622688	61,997182	1,037273131
46	4	A	15	65,27	43,9725	50,138344	0,46249	52,530975	68,41	-1,44	57,95		1	19,26	9,35		6,498	0,4341667	9,9555692	29,508197	60,536234	1,051770993

Supplementary Table S1. Raw data of egg quality parameters with ATOL (Animal Trait Ontology for Livestock) descriptors, for hen.

Hen age<br>wk	Pen<br>no.	Genotype	Replicate	ATOL_ 0001880<br>**Egg weight**<br>g	ATOL_ 0002297<br>**Egg diameter**<br>mm	ATOL_ 0002119<br>**Shell strength**<br>N	ATOL_ 0002126<br>**Shell fracture**<br>mm	ATOL_ 0002122<br>**Shell modulus**<br>N/mm	ATOL_ 0002074<br>**Yolk lightness**<br>L*	ATOL_ 0002073<br>**Yolk redness**<br>a*	ATOL_ 0002075<br>**Yolk yellow**<br>b*	ATOL_ 0005600<br>**Blood spot**<br>1=present	ATOL_ 0005600<br>**Meat spot**<br>1=present	ATOL_ 0001883<br>**Yolk weight**<br>g	ATOL_ 0005587<br>**Albumen pH**<br>-	ATOL_ 0001938<br>**Albumen DM**<br>% (w/w)	ATOL_ 1881<br>**Shell weight**<br>g	ATOL_ 0002121<br>**Shell thickness**<br>mm	ATOL_ 1881<br>**Shell**<br>% (w/w)	ATOL_ 0001883<br>**Yolk**<br>% (w/w)	ATOL_ 0001882<br>**Albumen**<br>% (w/w)	ATOL_ 0002125<br>**Shell-to-egg-compression**<br>%
46	5	B	1	67,778	44,907497	40,079796	0,369995	75,054924	64,85	-2,13	55,55	1		17,346	9,29	13,023478	6,268	0,4216667	9,2478385	25,592375	65,159786	0,823904748
46	5	B	2	63,254	44,502502	41,58107	0,444988	56,301941	56,04	-1,95	37,19			19,766	9,31	13,043478	5,524	0,4008333	8,7330446	31,248617	60,018339	0,999916814
46	5	B	3	69,731	45,785	38,878773	0,39999	101,33382	54,42	-0,09	49,54			16,212	9,38	12,29137	6,454	0,4141667	9,2555678	23,249344	67,495088	0,873626734
46	5	B	4	66,314	44,449997	42,93222	0,347485	82,584045	64,43	-0,97	59,86			18,719	9,4	12,31137	6,401	0,425	9,6525621	28,227825	62,119613	0,781743585
46	5	B	5	66,738	45,157497	35,125584	0,372494	78,837746	51,94	1,1	46,12			17,203	9,33	12,215679	5,747	0,3933333	8,6112859	25,776919	65,611795	0,824877428
46	5	B	6	63,168	43,195	47,436047	0,447498	82,517654	60,39	-4,97	53,31			17,745	9,37	12,257679	6,684	0,4666667	10,581307	28,091755	61,326938	1,035994907
46	5	B	7	67,602	45,372498	44,583622	0,457493	78,822708	57,68	-2,31	56,5		1	18,265	9,37	12,657208	5,969	0,4266667	8,8296204	27,018431	64,151948	1,008304634
46	5	B	8	63,947	44,129997	33,324055	0,389992	60,038315	65,72	-3,36	50,36	1		18,229	9,41	12,699208	5,065	0,365	7,9206218	28,506419	63,572959	0,883734481
46	5	B	9	66,548	44,649998	38,428391	0,395	75,069244	63,51	-1,56	60,66	1		17,176	9,4	12,846058	5,83	0,4075	8,7605939	25,809942	65,429464	0,884658494
46	5	B	10	68,591	44,454998	40,680305	0,469986	60,032784	61,6	0,89	55,9			17,743	9,44	12,888058	6,098	0,4033333	8,8903792	25,867825	65,241796	1,057217458
46	5	B	11	61,447	43,674999	30,471628	0,344986	71,298912	49,16	-0,23	48,82			16,577	9,3	12,349907	5,208	0,3766667	8,4755969	26,977721	64,546683	0,78989355
46	5	B	12	68,459	44,805	40,079796	0,402489	75,069244	64,05	-3,74	51,75			18,54	9,36	12,409907	5,961	0,3983333	8,7074015	27,081903	64,210695	0,898312688
46	5	B	13	62,845	44,197498	37,978008	0,39999	56,296574	65,54	-2,28	55,68		1	16,403	9,37	12,641338	5,278	0,3841667	8,3984406	26,100724	65,500835	0,90500598
46	5	B	14	67,12	44,224998	39,479286	0,407486	56,296562	66,57	-5,15	46,64			18,429	9,41	12,683338	6,205	0,4141667	9,2446365	27,456794	63,29857	0,921392919
46	5	B	15	62,161	43,4025	38,428391	0,412498	63,82103	62,98	-5,05	49,49			16,765	9,43		5,705	0,3916667	9,1777803	26,970287	63,851933	0,950401475
46	6	C	1	59,098	43,282497	39,479286	0,429989	75,070778	56,36	-0,68	57,4			16,461	9,38	12,828837	5,106	0,3841667	8,6398863	27,853734	63,506379	0,993447767
46	6	C	2	64,233	44,7925	53,891537	0,459995	63,827122	64,19	-3,4	51,41	1		17,956	9,4	12,848837	6,051	0,425	9,4203914	27,954478	62,62513	1,026946475
46	6	C	3	63,872	43,894997	39,179031	0,432487	56,285313	59,57	-4,07	40,05			16,64	9,19	13,294936	5,276	0,3675	8,2602705	26,052104	65,687625	0,985276295
46	6	C	4	58,952	43,889999	31,222267	0,404991	52,532211	58,19	-0,35	44,36			17,028	9,21	13,314936	4,583	0,4033333	7,7741213	28,884516	63,341362	0,922740964
46	6	C	5	64,163	43,965	33,474182	0,357502	56,274319	70,57	-3,68	53,26		1	17,399	9,32	13,196158	5,677	0,3991667	8,8477783	27,116874	64,035347	0,81315137
46	6	C	6	58,348	43,685001	44,733749	0,362492	78,845276	64,13	-3,32	43,31			16,796	9,36	13,238158	5,684	0,4308333	9,7415507	28,785905	61,472544	0,829785949
46	6	C	7	64,115	43,855	44,283367	0,389999	78,822708	52,62	-1,73	54,98			18,424	9,3	13,559247	5,996	0,4233333	9,3519457	28,735865	61,912189	0,889291985
46	6	C	8	56,672	42,115002	31,822777	0,317486	90,071838	68,53	-4,87	49,19			16,167	9,34	13,601247	5,036	0,3933333	8,8862225	28,527315	62,586462	0,753854885
46	6	C	9	61,269	43,157501	43,082348	0,404987	78,830215	62,06	-1,94	48,43			16,79	9,41	13,71791	6,043	0,4216667	9,8630629	27,403744	62,733193	0,938393073
46	6	C	10	63,991	44,2775	49,838314	0,417492	75,076416	58,44	-2,21	51,47			16,633	9,45	13,75991	6,248	0,4233333	9,763873	25,992718	64,243409	0,942898763
46	6	C	11	56,549	42,777508	38,128361	0,427486	78,822708	61,28	-3,8	52,56			15,751	9,41	12,473601	4,994	0,375	8,8312791	27,85372	63,315001	0,999324224
46	6	C	12	67,185	45,535004	44,884106	0,394985	78,815186	62,85	-4,11	51,53		1	17,966	9,47	12,533601	6,161	0,3958333	9,1702017	26,741088	64,08871	0,86743157
46	6	C	13	60,726	43,657501	41,28104	0,419998	63,798065	58,36	-4,75	42,46			14,742	9,35	12,71768	5,488	0,4166667	9,0373152	24,276257	66,686428	0,962029412
46	6	C	14	63,803	44,295002	45,934998	0,409985	67,55587	58,85	-2,26	48,21			16,73	9,39	12,75968	6,161	0,4233333	9,6562858	26,221338	64,122377	0,925578466
46	6	C	15	56,743	42,587505	33,174152	0,352501	82,560425	62,54	-5,54	37,63			14,14	9,25		4,92	0,4025	8,670673	24,919373	66,409954	0,827709912
46	7	A	1	63,592	42,695	39,179256	0,372486	86,321396	63,09	-0,87	58,76			20,376	9,27	13,142462	6,456	0,445	10,15222	32,041766	57,806013	0,872434711
46	7	A	2	62,957	44,215	50,288696	0,43499	63,797592	65,84	-1,25	59,43			18,867	9,29	13,162462	6,279	0,4466667	9,973474	29,968073	60,058453	0,983806401
46	7	A	3	72,686	46,205002	50,138569	0,417496	112,60385	59,7	-1,71	63,36			20,081	9,37	12,608297	7,163	0,4633333	9,8547176	27,627053	62,518229	0,903573167
46	7	A	4	65,665	43,9575	38,428616	0,349983	71,290009	62,46	-1,77	53,6			20,15	9,39	12,628297	6,225	0,42	9,479936	30,686058	59,834006	0,796184951
46	7	A	5	69,387	46,227501	38,578743	0,439983	93,827599	66,78	-2,72	53,81			19,63	9,47	13,744542	5,683	0,3833333	8,190295	28,290602	63,519103	0,951777601
46	7	A	6	68,222	44,945	41,431168	0,452496	52,553486	60,56	3,38	65,52	1		20,054	9,51	13,786542	5,913	0,4	8,6672921	29,39521	61,937498	1,006777172
46	7	A	7	64,937	43,82	60,347248	0,442497	63,82103	68,75	-0,62	57,9			17,444	9,34	13,538322	6,826	0,4698333	10,511727	26,862959	62,625314	1,009806025
46	7	A	8	67,336	45,017502	37,077469	0,377487	63,808861	61,16	-2,96	48,24			18,894	9,38	13,580322	5,876	0,4116667	8,7263871	28,059285	63,214328	0,838533866
46	7	A	9	72,906	45,950001	51,189465	0,384995	123,86426	58,86	-0,73	64,26			19,645	9,39	12,743004	7,526	0,4716667	10,322882	26,945656	62,731462	0,837856347
46	7	A	10	71,056	44,250004	38,878998	0,407486	60,043945	64,64	-2,41	53,88			20,617	9,43	12,785004	6,499	0,4416667	9,1463071	29,015143	61,83855	0,920872233
46	7	A	11	68,685	44,8475	38,428616	0,437485	67,563141	65,26	-1,99	55,41			18,553	9,38	13,325408	5,946	0,4208333	8,656912	27,01172	64,331368	0,975494732
46	7	A	12	72,062	45,5425	54,492271	0,419994	90,09169	63,52	-2,83	51,07			19,687	9,44	13,385408	7,831	0,4983333	10,867031	27,31953	61,813438	0,922202338
46	7	A	13	67,197	44,705002	50,88921	0,470001	67,562325	63,66	-2,24	55,48			19,534	9,49	12,84545	6,419	0,4316667	9,5525098	29,06975	61,37774	1,051338729
46	7	A	14	62,237	43,907505	48,637295	0,382496	71,308975	53,19	0,91	52,05			15,132	9,27	13,679583	5,964	0,4308333	9,5827241	24,313511	66,103765	0,871140367
46	7	A	15	64,568	44,110001	44,884106	0,392494	71,275223	64,69	2,9	64,41			19,22	9,54		6,355	0,4316667	9,8423368	29,767067	60,390596	0,88980728

Supplementary Table S1. Raw data of egg quality parameters with ATOL (Animal Trait Ontology for Livestock) descriptors, for hen.

				ATOL_ 0001880	ATOL_ 0002297	ATOL_ 0002119	ATOL_ 0002126	ATOL_ 0002122	ATOL_ 0002074	ATOL_ 0002073	ATOL_ 0002075	ATOL_ 0005600	ATOL_ 0005600	ATOL_ 0001883	ATOL_ 0005587	ATOL_ 0001938	ATOL_ 1881	ATOL_ 0002121	ATOL_ 1881	ATOL_ 0001883	ATOL_ 0001882	ATOL_ 0002125
**Hen age**	**Pen**	**Genotype**	**Replicate**	**Egg weight**	**Egg diameter**	**Shell strength**	**Shell fracture**	**Shell modulus**	**Yolk lightness**	**Yolk redness**	**Yolk yellow**	**Blood spot**	**Meat spot**	**Yolk weight**	**Albumen pH**	**Albumen DM**	**Shell weight**	**Shell thickness**	**Shell**	**Yolk**	**Albumen**	**Shell-to-egg-compression**
wk	no.			g	mm	N	mm	N/mm	L*	a*	b*	1=present	1=present	g	-	% (w/w)	g	mm	% (w/w)	% (w/w)	% (w/w)	%
46	8	D	1	56,033	41,615002	40,380276	0,417488	101,33382	64,82	-3	51,86			14,726	9,36	12,455163	5,513	0,4275	9,838845	26,280942	63,880213	1,003215139
46	8	D	2	65,806	45,722504	50,288696	0,417496	123,87608	59,67	-2,1	56,93		1	17,026	9,38	12,475163	6,29	0,4408333	9,5583989	25,873021	64,56858	0,913108346
46	8	D	3	59,768	43,827503	48,33704	0,444996	63,803226	56,41	-4,04	43,65			14,933	9,25	12,607859	6,003	0,455	10,043836	24,984942	64,971222	1,015335051
46	8	D	4	56,734	43,275002	59,596611	0,46249	75,069244	62,91	-2,83	47,34			15,496	9,27	12,627859	6,029	0,4458333	10,626785	27,313428	62,059788	1,068723232
46	8	D	5	59,604	43,945	52,690739	0,387497	75,062088	58,26	-1,04	54,41			18,903	9,34	12,543767	6,534	0,4425	10,962352	31,714314	57,323334	0,881777222
46	8	D	6	67,507	44,872501	43,983337	0,367493	75,069252														0,818971512
46	8	D	7	58,403	42,995003	58,545715	0,422493	97,590027	62,08	-3,31	53,66			15,098	9,38	13,10773	6,494	0,48	11,119292	25,851412	63,029296	0,982656054
46	8	D	8	60,88	43,052502	50,438824	0,449997	71,322586	63,74	-2,85	52,69			17,485	9,42	13,14973	6,294	0,4583333	10,338371	28,720434	60,941196	1,045228452
46	8	D	9	59,214	43,790001	48,93755	0,402489	63,80278	60,53	-1,03	52,78			16,407	9,37	13,639086	5,956	0,4533333	10,058432	27,707974	62,233593	0,919134485
46	8	D	10	63,332	44,800003	41,130913	0,397495	75,076408	61,96	-3,13	52,63			16,514	9,41	13,681086	5,54	0,4066667	8,7475526	26,075286	65,177162	0,887265566
46	8	D	11	59,73	43,990002	40,230148	0,34499	71,315781	57,91	-1,59	62,78			16,62	9,41	12,657945	5,54	0,4091667	9,2750712	27,825213	62,899715	0,784246384
46	8	D	12	57,426	43,0625	61,097885	0,442486	86,321404	61,6	-1,96	50,82			15,675	9,47	12,717945	6,583	0,4833333	11,463449	27,295998	61,240553	1,027543687
46	8	D	13	65,03	45,43	63,95031	0,407486	112,62537	53,98	-1,5	53,26			15,845	9,4	13,971695	7,249	0,4816667	11,147163	24,365677	64,48716	0,896953555
46	8	D	14	63,856	44,537502	45,634743	0,417496	63,814945	59,52	-2,53	52,7			16,19	9,44	14,013695	6,163	0,4266667	9,6514032	25,353921	64,994676	0,93740327
46	8	D	15	62,784	43,932503	31,37262	0,292503	67,555878	66,64	-2,38	60,03		1	15,259	9,38		6,391	0,4433333	10,179345	24,303963	65,516692	0,665800899
46	9	D	1	64,433	45,052502	52,090229	0,449986	82,586296	66,45	-1,33	55,87			15,59	9,32	12,429024	6,374	0,435	9,8924464	24,195676	65,911877	0,998803574
46	9	D	2	62,842	44,995003	60,197121	0,454998	67,568764	63,71	-0,65	50,95			15,118	9,34	12,449024	6,441	0,4533333	10,249515	24,057159	65,693326	1,011218957
46	9	D	3	58,809	42,827499	51,789974	0,459984	75,076408	61,29	-1,87	54,24			15,161	9,32	12,462036	6,208	0,4616667	10,556207	25,780068	63,663725	1,074038902
46	9	D	4	61,469	43,540001	50,438824	0,399998	93,827606	64,04	-2,27	51,62			16,873	9,34	12,482036	6,015	0,445	9,7854203	27,449609	62,764971	0,918690838
46	9	D	5	61,386	43,615002	48,787422	0,384998	86,337875	59,21	-3,27	52,69			16,834	9,28	12,622993	6,226	0,445	10,142378	27,423191	62,434431	0,882719207
46	9	D	6	62,799	44,1875	51,940102	0,439999	56,301926	58,69	-0,22	59,39			17,446	9,32	12,664993	5,984	0,4283333	9,5288142	27,780697	62,690489	0,995754455
46	9	D	7	59,533	43,7575	39,929893	0,375	67,55587	64,93	-2,11	48,64			16,282	9,21	13,241005	5,665	0,435	9,5157308	27,349537	63,134732	0,856995944
46	9	D	8	64,986	45,170002	48,637295	0,43499	63,802773	58,81	0,28	48,92			15,299	9,25	13,283005	6,253	0,4275	9,6220724	23,541994	66,835934	0,963006378
46	9	D	9	60,921	43,16	45,334488	0,39999	101,33382	68,14	-2,62	57,34		1	17,185	9,35	12,704031	5,672	0,4166667	9,3104184	28,208664	62,480918	0,92676089
46	9	D	10	64,972	44,3325	55,693291	0,397491	86,329628	58,57	0,51	48,75		1	16,38	9,39	12,746031	6,613	0,4483333	10,178231	25,21086	64,610909	0,896613094
46	9	D	11	62,122	44,5625	65,451591	0,417492	82,57618	57,47	-0,72	53,6			16,053	9,36	13,603994	6,99	0,4941667	11,252052	25,841087	62,906861	0,936868443
46	9	D	12	67	44,107502	53,891762	0,414997	67,555878	64,93	1,26	55,23			13,996	9,42	13,663994	6,668	0,4533333	9,9522388	20,889552	69,158209	0,940876226
46	9	D	13	60,325	43,9175	48,487167	0,367485	75,083572	58,97	-0,67	56,7			17,771	9,38	13,229883	5,925	0,435	9,8217986	29,458765	60,719436	0,836762111
46	9	D	14	63,115	44,225002	48,186913	0,409992	56,301941	63,47	0,32	57			14,509	9,42	13,271883	6,11	0,4266667	9,6807415	22,988196	67,331062	0,927059314
46	9	D	15	59,934	42,709999	40,980785	0,374985	90,074501	62,39	-1,35	51,94			15,662	9,31		5,663	0,4166667	9,4487269	26,132079	64,419194	0,877979416
46	11	C	1	69,808	44,705002	42,331936	0,389992	60,049671	60,22	-4,07	53,35			17,448	9,32	12,020247	6,965	0,435	9,9773665	24,99427	65,028364	0,872367705
46	11	C	2	63,059	44,190002	32,423515	0,30249	82,560417	57,92	-2,81	58,63			17,315	9,34	12,040247	5,67	0,3908333	8,9915793	27,458412	63,550009	0,684521354
46	11	C	3	70,348	45,237499	31,672874	0,33749	67,55587	63,23	-3,18	49,98			22,312	9,28	12,723447	6,322	0,405	8,9867516	31,716609	59,29664	0,746040359
46	11	C	4	65,164	44,847504	47,136017	0,407482	75,076416	58,33	-0,57	65,6			17,524	9,3	12,743447	5,719	0,3975	8,7763182	26,892149	64,331533	0,908594601
46	11	C	5	62,63	43,812504	31,072365	0,399994	52,563511	60,35	-4,49	43,33			20,427	9,33	11,686317	5,222	0,3808333	8,3378573	32,61536	59,046783	0,912967677
46	11	C	6	69,237	45,540001	43,382828	0,46249	78,830231	63,23	-1,39	52,7		1	18,591	9,37	11,728317	6,033	0,4083333	8,7135491	26,85125	64,435201	1,015568708
46	11	C	7	61,883	43,924999	43,2327	0,432484	60,055397	55,96	-1,3	58,96			17,199	9,11	12,6083	5,711	0,4133333	9,2287058	27,79277	62,978524	0,984596494
46	11	C	8	57,565	42,695	29,120705	0,304989	82,560417	59,32	-1,05	50,79			16,738	9,15	12,6503	5,191	0,3841667	9,0176322	29,076696	61,905672	0,7143436
46	11	C	9	70,878	45,834999	33,024025	0,334988	108,8504	64,23	-3,68	50,32	1		21,049	9,37	13,185126	5,007	0,3925	7,0642512	29,697508	63,23824	0,730856348
46	11	C	10	64,15	44,137501	44,733978	0,394989	78,830231	66,45	-2,78	54,66			17,82	9,41	13,227126	5,784	0,3975	9,0163679	27,778644	63,204988	0,894905672
46	11	C	11	61,517	43,52	34,074917	0,352497	71,315781	57,87	1,13	45,85	1		16,76	9,38	12,671909	5,222	0,3833333	8,4887104	27,244502	64,266788	0,809965533
46	11	C	12	64,278	44,555	35,876446	0,434986	60,061119	66,15	-4,79	51,61		1	19,14	9,44	12,731909	5,046	0,3766667	7,8502754	29,776907	62,372818	0,976289979
46	11	C	13	60,096	42,205002	30,471853	0,347488	82,576172	68,09	-2,18	56,33	1		17,454	9,41	12,490585	5,466	0,405	9,0954473	29,04353	61,861022	0,823333689
46	11	C	14	67,537	45,440002	44,43372	0,419998	101,34347	64,73	-4,53	52,62		1	18,972	9,45	12,532585	5,719	0,3891667	8,4679509	28,091268	63,440781	0,924291333
46	11	C	15	63,04	45,100002	51,039337	0,464989	67,562317	67,52	-2,64	50,33	1		15,826	9,28		5,97	0,4066667	9,4701777	25,104695	65,425127	1,031017693

Supplementary Table S1. Raw data of egg quality parameters with ATOL (Animal Trait Ontology for Livestock) descriptors, for hen.

				ATOL_ 0001880	ATOL_ 0002297	ATOL_ 0002119	ATOL_ 0002126	ATOL_ 0002122	ATOL_ 0002074	ATOL_ 0002073	ATOL_ 0002075	ATOL_ 0005600	ATOL_ 0005600	ATOL_ 0001883	ATOL_ 0005587	ATOL_ 0001938	ATOL_ 1881	ATOL_ 0002121	ATOL_ 1881	ATOL_ 0001883	ATOL_ 0001882	ATOL_ 0002125
**Hen age**	**Pen**	**Genotype**	**Replicate**	**Egg weight**	**Egg diameter**	**Shell strength**	**Shell fracture**	**Shell modulus**	**Yolk lightness**	**Yolk redness**	**Yolk yellow**	**Blood spot**	**Meat spot**	**Yolk weight**	**Albumen pH**	**Albumen DM**	**Shell weight**	**Shell thickness**	**Shell**	**Yolk**	**Albumen**	**Shell-to-egg-compression**
wk	no.			g	mm	N	mm	N/mm	L*	a*	b*	1=present	1=present	g	-	% (w/w)	g	mm	% (w/w)	% (w/w)	% (w/w)	%
46	12	B	1	62,559	44,07	39,029129	0,387493	60,055401	64,62	-1,79	55,08	1		18,151	9,35	12,704179	5,933	0,4083333	9,4838472	29,014211	61,501942	0,879267075
46	12	B	2	60,996	42,767502	33,624535	0,404991	71,308975	56,73	-2,97	53,44			19,199	9,37	12,724179	5,178	0,3716667	8,4890813	31,475834	60,035084	0,94695968
46	12	B	3	67,016	44,82	25,217388	0,334991	60,043945	49,58	0,31	51,02	1			9,29	12,407582	5,1335	0,3691667	7,660111			0,747414101
46	12	B	4	65,399	44,567501	41,431168	0,414997	60,049667	64,45	-0,81	48,99	1		19,066	9,31	12,427582	5,722	0,3991667	8,7493693	29,153351	62,09728	0,931165066
46	12	B	5	66,079	44,3475	39,629639	0,387493	67,555885	60,69	-1,03	52,24	1		18,697	9,36	12,713177	5,793	0,3975	8,7667792	28,29492	62,938301	0,87376515
46	12	B	6	68,92	43,802502	43,2327	0,442486	63,803226	49,96	4,41	40,17			19,931	9,4	12,755177	6,207	0,415	9,006094	28,919037	62,074869	1,010184304
46	12	B	7	60,211	43,745003	38,128361	0,437489	56,285316	62,08	0,98	61,37			16,449	9,37	12,527394	4,762	0,3533333	7,9088539	27,318928	64,772218	1,000089084
46	12	B	8	65,046	44,655003	37,677979	0,384987	63,814945	62,61	-0,91	55,82			18,686	9,41	12,569394	5,62	0,3966667	8,6400394	28,727362	62,632598	0,862136321
46	12	B	9	66,881	44,927502	35,125809	0,419998	60,061134	62,23	0,03	55,13				9,2	12,350615	5,311	0,3733333	7,9409698			0,93483497
46	12	B	10	60,842	42,607502	37,828106	0,382484	82,584053	51,96	1,03	43,98	1		16,703	9,24	12,392615	5,753	0,425	9,4556392	27,453075	63,091286	0,897691679
46	12	B	11	64,84	44,299999	15,759348	0,234993	60,049671	58,5	-2,46	47,7	1		17,275	9,4	12,870875	5,237	0,3725	8,0768044	26,642505	65,280691	0,530458251
46	12	B	12	67,538	44,857502	34,074917	0,364994	60,038223	59,39	-0,03	50,26			18,371	9,46	12,930875	6,284	0,4166667	9,3043916	27,200983	63,494625	0,813674377
46	12	B	13	59,899	42,202499	36,026573	0,397491	90,100273	51,63	-2,04	41,51				9,4	12,850854	5,328	0,3875	8,8949732			0,941866026
46	12	B	14	65,553	44,530003	39,479511	0,33749	86,313164	50,08	-0,5	50,86				9,44	12,892854	5,789	0,3983333	8,8310222			0,757893504
46	12	B	15	65,663	44,690002	37,527851	0,359993	75,062088	67,1	-3,96	55,43			17,452	9,39		5,678	0,4041667	8,6471833	26,578134	64,774683	0,80553364
54	1	C	1	63,618	44,767502	35,275486	0,319996	67,555885	61,02	-4,36	51,18			17,815	9,26	12,808722	5,959	0,4066667	9,3668459	28,003081	62,630073	0,7147953
54	1	C	2	66,502	43,500004	32,423061	0,344997	75,069244	61,99	-4,67	49,65			18,146	9,28	12,828722	5,983	0,425	8,9967219	27,286397	63,716881	0,793096479
54	1	C	3	64,126	44,970001	37,077019	0,417492	75,076401	62	-3,72	47,51			17,351	9,03	13,321222	4,993	0,3541667	7,7862334	27,057668	65,156099	0,928378899
54	1	C	4	62,838	44,665001	37,527401	0,40749	86,307533	58	0,46	51,91			17,391	9,05	13,341222	5,277	0,375	8,3977848	27,675929	63,926287	0,912325066
54	1	C	5	60,035	44,005001	31,972679	0,424995	56,240307	66,61	-4,27	58,01			16,802	9,26	12,230443	5,063	0,37	8,4334138	27,987008	63,579579	0,965787957
54	1	C	6	64,243	44,755001	39,629189	0,329994	97,590012							9,3	12,272443	5,696	0,3875	8,8663356			0,73733436
54	1	C	7	62,74	43,092503	37,977783	0,302498	90,083092	66,3	-2,1	64,02			17,092	9,27	11,964019	6,766	0,46	10,784189	27,242588	61,973223	0,701973612
54	1	C	8	68,651	44,397503	37,227146	0,294994	112,59315	64,15	-3,06	52,39		1	16,775	9,31	12,006019	6,999	0,4533333	10,195045	24,435187	65,369769	0,664438268
54	1	C	9	67,795	45,972504	36,776764	0,39999	97,580704	64,65	-3,57	47,84			17,782	9,24	12,393384	5,749	0,3908333	8,4799764	26,229073	65,290951	0,870063549
54	1	C	10	62,956	43,872501	46,985439	0,429989	60,04966	66,57	-4,22	52,27		1	17,974	9,28	12,435384	5,806	0,4083333	9,222314	28,550098	62,227588	0,980087732
54	1	C	11	65,565	44,272503	30,171148	0,502499	11,242434	60,94	-4,12	52,64			17,769	9,24	13,26806	7,159	0,4808333	10,918935	27,10135	61,979715	1,135013758
54	1	C	12	63,537	44,335003	42,781868	0,402493	86,321396	61,1	-1,26	53,72			16,985	9,3	13,32806	5,717	0,4141667	8,9979067	26,732455	64,269638	0,907844756
54	1	C	13	64,603	44,2225	50,738628	0,367485	75,06208	58,42	-1,48	49,45			18,908	9,09	12,932172	6,506	0,4525	10,07074	29,267991	60,66127	0,830991011
54	1	C	14	67,922	45,592503	52,239906	0,427494	120,12222	59,66	-1,75	51,75			18,099	9,13	12,974172	6,4	0,4475	9,422573	26,646742	63,930685	0,937640998
54	1	C	15	63,523	43,472504	36,326378	0,389988	71,322578	64,06	-1,13	59	1		17,179	9,29		5,795	0,41	9,12268	27,043748	63,833572	0,897091182
54	2	D	1	66,893	44,935001	47,135567	0,442486	78,815186	60,3	-2,29	49,62			18,112	9,18	12,565819	6,329	0,4316667	9,4613786	27,076077	63,462545	0,98472458
54	2	D	2	62,497	44,16	50,738628	0,394985	67,568756	66,04	-2,47	53,53			16,377	9,2	12,585819	6,527	0,4475	10,443701	26,204458	63,351841	0,89444067
54	2	D	3	62,717	43,945004	48,636845	0,412483	67,575203	62,45	-1,82	47,74			17,333	9,07	12,66845	5,92	0,4166667	9,439227	27,636845	62,923928	0,938634572
54	2	D	4	66,461	45,1675	46,38493	0,39999	63,814945	58,12	-3,51	52,59			16,68	9,09	12,68845	6,465	0,425	9,7275094	25,097426	65,175065	0,885570377
54	2	D	5	62,542	43,737503	55,542713	0,412487	86,321404	63,02	-1,35	51,09	1		16,639	9,19	12,306929	6,514	0,4533333	10,415401	26,604522	62,980077	0,94309682
54	2	D	6	66,505	45,567505	49,837864	0,424992	108,84003	62,57	-0,13	53,63			17,977	9,23	12,348929	6,602	0,4483333	9,9270732	27,03105	63,041877	0,932664626
54	2	D	7	59,692	43,232502				65,36	-3,66	47,59			16,639	9,16	11,548381	5,905	0,4216667	9,8924479	27,874757	62,232795	
54	2	D	8	65,426	45,397503	50,88876	0,4375	90,083092	62,88	-5,61	40,14			19,362	9,2	11,590381	6,157	0,4316667	9,4106319	29,593739	60,995629	0,963709392
54	2	D	9	67,649	44,965	51,639397	0,412498	60,055401	67,51	-5,4	46,37			19,939	9,22	11,351792	6,98	0,455	10,317965	29,474198	60,207838	0,917375737
54	2	D	10	66,536	45,197502	48,186462	0,412498	78,815186							9,26	11,393792	6,774	0,4625	10,180955			0,912656633
54	2	D	11	69,653	45,837502	55,542713	0,46249	127,62989	59,99	-2,45	54,93			18,427	9,2	11,359522	6,998	0,4483333	10,046947	26,455429	63,497624	1,008977322
54	2	D	12	65,29	43,7575	51,639397	0,467487	67,550247	60,69	-4,47	49,98			16,957	9,26	11,419522	6,305	0,4383333	9,6569153	25,971818	64,371267	1,068358567
54	2	D	13	64,987	44,535	53,140671	0,424992	86,33786	63,22	-2,97	53,53			19,968	9,16	11,641238	6,846	0,45	10,534415	30,726145	58,73944	0,954287639
54	2	D	14	63,128	44,657505	39,479061	0,382496	71,322578	61,08	-3,78	48,82			16,155	9,2	11,683238	5,674	0,3933333	8,9880877	25,590863	65,421049	0,856510009
54	2	D	15	63,328	43,922504	55,392586	0,424995	78,817085	64,03	-3,32	46,46			16,855	9,17		6,666	0,46	10,52615	26,615399	62,858451	0,967601938

Supplementary Table S1. Raw data of egg quality parameters with ATOL (Animal Trait Ontology for Livestock) descriptors, for hen.

Hen age wk	Pen no.	Genotype	Replicate	ATOL_0001880 Egg weight g	ATOL_0002297 Egg diameter mm	ATOL_0002119 Shell strength N	ATOL_0002126 Shell fracture mm	ATOL_0002122 Shell modulus N/mm	ATOL_0002074 Yolk lightness L*	ATOL_0002073 Yolk redness a*	ATOL_0002075 Yolk yellow b*	ATOL_0005600 Blood spot 1=present	ATOL_0005600 Meat spot 1=present	ATOL_0001883 Yolk weight g	ATOL_0005587 Albumen pH -	ATOL_0001938 Albumen DM % (w/w)	ATOL_1881 Shell weight g	ATOL_0002121 Shell thickness mm	ATOL_1881 Shell % (w/w)	ATOL_0001883 Yolk % (w/w)	ATOL_0001882 Albumen % (w/w)	ATOL_0002125 Shell-to-egg-compression %
54	3	B	1	72,069	45,327503	29,870893	0,409988	75,069237	57,05	0,32	59,46	1		19,163	9,16	11,509608	5,655	0,355	7,846647	26,589796	65,563557	0,904501622
54	3	B	2	72,356	45,450001	29,270382	0,329994	86,337875	63,18	-1,26	55,29	1		19,49	9,18	11,529608	6,209	0,4075	8,5811819	26,93626	64,482558	0,72605939
54	3	B	3	70,779	46,84	37,077019	0,354992	108,84003	63,82	-3	55,91			20,475	9,19	11,940904	6,343	0,41	8,9616977	28,928072	62,11023	0,757882152
54	3	B	4	70,612	46,2075	27,468851	0,344997	93,819672	60,82	-0,77	55,64	1		19,077	9,21	11,960904	5,56	0,3783333	7,8740157	27,016654	65,10933	0,746625548
54	3	B	5	69,914	45,657501	36,326378	0,389996	101,32417	59,99	-2,79	48,08			20,563	9,23	11,426811	6,311	0,4108333	9,0268044	29,411849	61,561347	0,85417728
54	3	B	6	67,124	44,360001	36,476509	0,45499	22,484869	67,78	-5,14	48,38	1		18,618	9,27	11,468811	6,243	0,4233333	9,3006972	27,736726	62,962577	1,025676262
54	3	B	7	66,766	45,212502	41,430717	0,427494	67,562325	58,05	-1,52	41,63	1		19,614	9,16	12,376661	5,845	0,3966667	8,7544559	29,377228	61,868316	0,945521661
54	3	B	8	70,746	44,375	31,22204	0,354996	60,055397	61,47	-1,57	56,56	1	1	20,865	9,2	12,418661	6,33	0,4175	8,9475023	29,492834	61,559664	0,799990986
54	3	B	9	71,423	45,210003	39,629189	0,402489	71,315781							9,13	12,23078	6,212	0,4133333	8,6974784			0,890265369
54	3	B	10	69,581	45,727509	36,026123	0,419994	97,573143	63,61	-1,04	55,95			18,804	9,17	12,27278	5,848	0,39	8,4045932	27,024619	64,570788	0,918471199
54	3	B	11	68,729	45,850002	37,077019	0,332493	112,59315	64,01	-2,07	56,77			17,145	9,15	11,700449	6,317	0,41	9,1911711	24,945802	65,863027	0,725175541
54	3	B	12	67,554	44,950001	39,929443	0,414997	59,976536	64,6	-2,38	51,66		1	19,844	9,21	11,760449	5,783	0,4008333	8,560559	29,375019	62,064423	0,923241359
54	3	B	13	71,506	44,620003	34,674976	0,384983	78,815186	62,82	-0,9	57,29			19,981	9,16	12,145172	6,108	0,3983333	8,5419405	27,94311	63,51495	0,862803617
54	3	B	14	68,707	45,587502	32,873447	0,355	97,580704	63,02	-2,17	50,75	1		21,779	9,2	12,187172	5,366	0,37	7,8099757	31,698371	60,491653	0,778722203
54	3	B	15	68,97	45,887501	32,272934	0,372486	105,09694	66,32	-3,8	55,01	1	1	17,658	9,21		5,741	0,3916667	8,3239089	25,602436	66,073655	0,811737384
54	4	A	1	67,147	45,100002	62,448586	0,484997	56,307297	56,64	-0,6	56,94			20,061	9,21	13,276164	7,003	0,4741667	10,429356	29,876242	59,694402	1,075381327
54	4	A	2	66,675	44,987503	50,138344	0,427502	48,799671	63,84	-2,99	54,75			16,587	9,22	12,600442	6,412	0,4383333	9,6167979	24,87739	65,505812	0,950268345
54	4	A	3	67,915	45,607498	49,387707	0,450005	97,599327	63,33	-2,43	53,99			17,28	9,15	12,48591	6,152	0,4175	9,0583818	25,443569	65,498049	0,986690829
54	4	A	4	70,477	44,907501	45,634518	0,424995	60,055401	66,91	-0,21	60,8			17,38	9,2	12,947693	6,732	0,4316667	9,5520524	24,660528	65,78742	0,946378646
54	4	A	5	67,295	44,530003	49,837864	0,379997	75,076408	65,46	-3,39	54,87			19,937	9,21	12,448287	7,115	0,4808333	10,572851	29,626272	59,800877	0,853350493
54	4	A	6	60,589	41,860001	39,479061	0,377495	97,599342	59,62	-2,56	54,08			19,507	9,25	12,490287	6,193	0,4666667	10,221327	32,195613	57,58306	0,90180361
54	4	A	7	72,224	46,3325	48,33659	0,484993	112,60385	57,3	-2,81	58,6	1	1	19,94	9,12	13,023925	6,066	0,4658333	8,3988702	27,608551	63,992579	1,046766309
54	4	A	8	62,788	43,93	49,988216	0,414997	63,808868	59,64	0,18	50,22		1	16,66	9,3		6,382	0,4466667	10,164363	26,533733	63,301905	0,944677897
54	4	A	9	70,008	45,665001	44,883652	0,464985	97,590019	62,77	-4,6	56,9			22,641	9,22	13,369119	5,895	0,3933333	8,4204662	32,34059	59,238944	1,018252469
54	4	A	10	66,481	45,530003	46,835312	0,467487	105,08692	65,86	-2,01	56,25			20,022	9,26	13,411119	6,271	0,415	9,4327703	30,116875	60,450354	1,026766899
54	4	A	11	68,966	45,350006	50,138119	0,389988	90,074501	58,54	-2,56	58,6			21,163	9,23	12,451805	7,385	0,5016667	10,708175	30,686135	58,60569	0,859951375
54	4	A	12	67,807	46,1875	37,527401	0,409992	116,33482	61,8	-3,79	54,17			20,286	9,29	12,511805	6,034	0,415	8,8987863	29,917265	61,183949	0,887668742
54	4	A	13	68,21	45,357502	47,736076	0,444996	75,06208	59,56	-4,4	46,12			20,396	9,2	12,210462	6,379	0,4241667	9,3520012	29,901774	60,746225	0,981085775
54	4	A	14	64,869	44,677502	51,038887	0,414989	86,321404	60,6	-2,89	53,68			20,272	9,24	12,252462	6,155	0,4333333	9,4883535	31,250674	59,260972	0,928854527
54	4	A	15	65,979	44,442501	39,779316	0,452496	67,506058	56,75	-1,99	48,84			20,262	9,26		5,85	0,4025	8,8664575	30,709771	60,423771	1,018160522
54	5	B	1	70,596	45,485004	40,079571	0,362492	116,34046	59,92	0,04	53,11			19,925	9,2	13,739162	6,433	0,4166667	9,1124143	28,223979	62,663607	0,796948374
54	5	B	2	69,146	45,695	46,234802	0,492489	101,3435	59,76	0,69	62,51			18,958	9,22	13,759162	5,733	0,3766667	8,2911521	27,417349	64,291499	1,077774374
54	5	B	3	62,011	43,1325	32,72332	0,414986	56,301941	62,69	-2,57	56,57			18,967	9,25	13,716125	5,44	0,3783333	8,7726371	30,586509	60,640854	0,962119052
54	5	B	4	67,09	44,195	31,822552	0,384995	56,307301	66,99	-0,9	58,66			17,263	9,27	13,736125	5,836	0,4033333	8,6987629	25,731107	65,57013	0,871127956
54	5	B	5	67,371	45,080002	39,178802	0,437485	60,061119	62,33	-1,18	53,13			17,775	9,17	13,317063	5,772	0,3933333	8,5674845	26,383756	65,04876	0,970463577
54	5	B	6	68,446	43,4575	32,573189	0,409996	67,516495	64,43	-0,34	52,77	1	1	20,399	9,21	13,359063	5,641	0,375	8,2415335	29,803056	61,95541	0,943441293
54	5	B	7	70,902	45,830002	47,585949	0,452492	97,550644	63,53	-1,79	53,1			18,785	9,22	12,964782	6,965	0,4475	9,8234182	26,494316	63,682266	0,987327035
54	5	B	8	67,334	43,952503	46,535057	0,377491	71,308975	66,96	-3,24	58,62			17,535	9,26	13,006782	6,906	0,455	10,256334	26,041821	63,701845	0,858861212
54	5	B	9	68,155	45,127502	51,489269	0,424988	63,81496	65,96	-0,45	54,22	1		18,322	9,22	14,094781	6,714	0,4333333	9,8510748	26,882841	63,266085	0,941749446
54	5	B	10	71,988	46,3475	41,130463	0,389992	127,61771	63,27	-2,21	59,79	1		18,943	9,26	14,136781	6,192	0,3941667	8,6014336	26,314108	65,084459	0,841452074
54	5	B	11	64,898	44,2925	40,379826	0,334984	86,321404	54,17	-0,46	57,37	1		19,215	9,29	12,690848	5,992	0,4066667	9,2329502	29,608	61,15905	0,756299599
54	5	B	12	71,486	45,4175				61,09	-1,43	56,8		1	20,02	9,35	12,750848	6,547	0,4158333	9,1584366	28,005484	62,83608	
54	5	B	13	61,264	43,0625	29,720764	0,387497	63,718838	63,95	-4,34	50,43	1		17,75	9,25	13,822352	5,585	0,3958333	9,1162836	28,972969	61,910747	0,899847896
54	5	B	14	66,418	44,215	49,537609	0,442497	60,049671	63,95	-4,5	42,35			17,809	9,29	13,864352	6,64	0,4533333	9,9972899	26,813514	63,189196	1,000784802
54	5	B	15	65,394	44,545002	38,278038	0,419994	78,77018	62,26	-3,56	59,05	1		17,575	9,33		5,523	0,3808333	8,445729	26,875554	64,678717	0,942853252

Supplementary Table S1. Raw data of egg quality parameters with ATOL (Animal Trait Ontology for Livestock) descriptors, for hen.

				ATOL_ 0001880	ATOL_ 0002297	ATOL_ 0002119	ATOL_ 0002126	ATOL_ 0002122	ATOL_ 0002074	ATOL_ 0002073	ATOL_ 0002075	ATOL_ 0005600	ATOL_ 0005600	ATOL_ 0001883	ATOL_ 0005587	ATOL_ 0001938	ATOL_ 1881	ATOL_ 0002121	ATOL_ 1881	ATOL_ 0001883	ATOL_ 0001882	ATOL_ 0002125
**Hen age**	**Pen**	**Genotype**	**Replicate**	**Egg weight**	**Egg diameter**	**Shell strength**	**Shell fracture**	**Shell modulus**	**Yolk lightness**	**Yolk redness**	**Yolk yellow**	**Blood spot**	**Meat spot**	**Yolk weight**	**Albumen pH**	**Albumen DM**	**Shell weight**	**Shell thickness**	**Shell**	**Yolk**	**Albumen**	**Shell-to-egg-compression**
wk	no.			g	mm	N	mm	N/mm	L*	a*	b*	1=present	1=present	g	-	% (w/w)	g	mm	% (w/w)	% (w/w)	% (w/w)	%
54	6	C	1	66,709	45,150002	33,62431	0,499989	52,480953	51,68	0,31	65,06			16,092	9,23	12,592291	5,572	0,385	8,352696	24,122682	67,524622	1,1073953
54	6	C	2	65,39	44,502502	47,285919	0,384998	82,57618	59,25	-1,38	59,4			17,839	9,25	12,612291	6,683	0,4508333	10,220217	27,28093	62,498853	0,865115404
54	6	C	3	63,994	43,019997	38,128136	0,379986	60,044037	65,88	-4,9	52,72			18,089	9,17	13,297241	6,148	0,4416667	9,6071507	28,266713	62,126137	0,883277607
54	6	C	4	68,893	44,807503	45,334263	0,352489	86,321396	65,47	-4,39	58,44			17,599	9,19	13,317241	7,178	0,4783333	10,419056	25,545411	64,035533	0,786674053
54	6	C	5	65,63	45,654999	51,18924	0,437504	105,10696	66,86	-3,92	52,36			18,494	9,22	12,891183	6,447	0,4383333	9,8232516	28,179186	61,997562	0,958282794
54	6	C	6	64,818	44,192505	41,430943	0,389999	60,043949	65,02	-0,62	61,93			17,025	9,26	12,933183	6,081	0,4166667	9,3816532	26,265852	64,352495	0,882500324
54	6	C	7	63,365	43,834999	43,232475	0,39999	67,568771	43,3	1,12	47,23			17,516	9,29	13,238833	6,223	0,4266667	9,820879	27,643021	62,5361	0,91249004
54	6	C	8	68,863	44,247498	53,591282	0,407486	71,315781	58,85	1,1	53,07			18,34	9,33	13,280833	7,226	0,4633333	10,493298	26,632589	62,874112	0,920924388
54	6	C	9	67,145	45,112503	40,680305	0,394989	60,055401	60,92	-1,72	48,05			17,789	9,22	14,138292	5,755	0,3766667	8,5710031	26,49341	64,935587	0,875564364
54	6	C	10	64,967	43,510002	47,886433	0,397488	75,069244	63,53	-3,72	46,11			17,331	9,26	14,180292	6,65	0,4425	10,235966	26,67662	63,087414	0,913555462
54	6	C	11	65,006	44,402496	44,883881	0,380005	75,069252	63,11	-2,82	57,57		1	17,71	9,18	9,476008	6,07	0,4066667	9,3375996	27,243639	63,418761	0,855819006
54	6	C	12	64,133	44,1875	46,835537	0,467491	60,049767	61,93	-5,04	47,62		1	19,029	9,24	9,536008	5,717	0,405	8,9142875	29,671152	61,41456	1,057971146
54	6	C	13	67,862	44,934998	45,184135	0,355007	71,308983	61,76	-3,81	52			17,577	9,18	13,108195	7,007	0,4616667	10,325366	25,901093	63,77354	0,790045657
54	6	C	14	64,773	44,962502	30,021246	0,342495	60,061119	64,43	-4,52	47,88		1	18,025	9,22	13,150195	5,09	0,3733333	7,8582125	27,827953	64,313834	0,761734745
54	6	C	15	62,169	43,1875	62,448811	0,469997	71,308975	61,43	-2,89	48,88			16,839	9,36		6,796	0,4716667	10,931493	27,085847	61,98266	1,088270912
54	7	A	1	67,835	43,707497	53,741409	0,4175	71,308983	60,63	-2,36	52,31		1	20,585	9,24	12,236326	7,055	0,4766667	10,400236	30,345692	59,254072	0,955213702
54	7	A	2	68,949	44,417503	47,285919	0,429996	67,575211	64,41	-2,75	57,84		1	21,451	9,26	12,256326	6,726	0,4458333	9,7550363	31,111401	59,133562	0,968077832
54	7	A	3	64,172	44,372498	39,329159	0,417503	60,066864	58,39	-1,76	51,95			18,441	9,25	12,225563	5,431	0,3883333	8,4631927	28,736832	62,799975	0,940904882
54	7	A	4	67,443	44,512497	54,492046	0,39249	86,313171	64,04	-2,06	54,15			15,714	9,12	14,104561	6,99	0,46	10,364308	23,299675	66,336017	0,881752376
54	7	A	5	66,93	45,779999	60,196896	0,484989	108,86077	57,89	-0,67	59,52			20,518	9,23	12,625856	6,682	0,4691667	9,9835649	30,655909	59,360526	1,059390587
54	7	A	6	64,351	45,305	53,291023	0,357491	75,06208	63,1	-2,26	56,23			19,393	9,27	12,667856	7,345	0,4966667	11,413964	30,136284	58,449752	0,789076261
54	7	A	7	67,06	45,139999	58,695618	0,467503	71,329391	68,61	-2,81	57,84			18,092	9,18	13,179413	6,775	0,4575	10,102893	26,978825	62,918282	1,035673483
54	7	A	8	63,318	43,415001	47,886433	0,46751	63,808853	57,93	-1,01	60,48			21,043	9,22	13,221413	5,711	0,465	9,0195521	33,233836	57,746612	1,076839777
54	7	A	9	65,848	44,970001	43,082348	0,389999	67,55587	52,13	-0,99	48,92			19,487	9,23	12,217181	6,294	0,435	9,5583769	29,593913	60,84771	0,867242587
54	7	A	10	69,938	44,894997	56,293575	0,447487	71,315781	62,53	1,56	61,21			20,056	9,27	12,259181	6,579	0,4141667	9,4069033	28,676828	61,916269	0,996741352
54	7	A	11	66,391	45,422501	58,995872	0,460007	60,055389	60,29	-0,15	57,73			21,085	9,19	12,653101	6,611	0,4458333	9,957675	31,758823	58,283502	1,012729352
54	7	A	12	67,998	45,067497	49,537834	0,467506	60,061119	59,58	-3,18	57,23			22,198	9,25	12,713101	6,221	0,4158333	9,1487985	32,645078	58,206124	1,037346272
54	7	A	13	62,91	43,504997	49,687962	0,379986	75,076401	60,28	-3,37	46,33		1	19,169	9,29	12,571073	6,986	0,4933333	11,104753	30,470513	58,424734	0,8734307
54	7	A	14	64,348	44,067497	54,341919	0,43	67,555885	67,54	-4,53	51,69			19,575	9,29		6,557	0,4716667	10,189905	30,420526	59,389569	0,975775865
54	7	A	15	65,437	44,974998	65,751617	0,407486	82,568291	60,26	-2,06	48,6		1	17,175	9,31		7,442	0,515	11,372771	26,246619	62,38061	0,906027834
54	8	D	1	64,547	45	49,687962	0,420002	56,318058	49,16	1,07	38,24				9,12	11,925484	6,259	0,455	9,6968101			0,933337778
54	8	D	2	62,945	44,357498	34,374947	0,382504	63,802773	66,86	-3,98	49,41			18,472	9,14	11,945484	5,367	0,39	8,5264914	29,346255	62,127254	0,862320954
54	8	D	3	63,511	44,450005	51,18924	0,407501	75,06208	64,75	-3,24	53,95			17,505	9,19	12,461993	6,202	0,4483333	9,7652375	27,562155	62,672608	0,916762552
54	8	D	4	64,136	45,142502	53,891537	0,434994	67,562325	67,54	-4,24	45,9			17,276	9,23	12,503993	6,493	0,4583333	10,123799	26,93651	62,939691	0,963601885
54	8	D	5	63,753	44,252502	51,639622	0,429993	78,815193	58,3	-3,75	56,21			16,361	9,21	12,663366	6,507	0,4475	10,206579	25,663106	64,130315	0,971680652
54	8	D	6	59,693	43,682503	51,639622	0,437496	71,31015	54,97	-3,81	52,95			16,877	9,25	12,705366	6,152	0,4483333	10,306066	28,272997	61,420937	1,001536015
54	8	D	7	66,722	45,905006	55,542938	0,410004	135,12465	61,03	-2,41	53,01			18,501	9	12,71143	6,583	0,4433333	9,866311	27,728485	62,405204	0,893157491
54	8	D	8	67,512	44,932503	37,227371	0,355	60,055401	63,66	-3,43	51,53			16,214	9,04	12,75343	6,36	0,4483333	9,4205475	24,016471	66,562981	0,790073947
54	8	D	9	63,183	43,970001	22,364737	0,237499	63,808861	61,72	-2,54	51,44			16,637	8,89	12,018193	6,175	0,4316667	9,7731985	26,33145	63,895352	0,540138719
54	8	D	10	54,975	42,817497	53,741409	0,439983	82,576164	61,22	-4,1	48,47			15,774	8,95	12,078193	5,762	0,4433333	10,481128	28,693042	60,82583	1,027577581
54	8	D	11	57,356	42,290001	57,344471	0,422493	86,321396	63,49	-2,81	50,47			15,039	9,18	12,239766	6,682	0,5333333	11,650045	26,220448	62,129507	0,999037574
54	8	D	12	65,419	44,540001	18,46142	0,162495	112,60385	63,62	-3,11	48,97			17,155	9,22	12,281766	7,307	0,4916667	11,169538	26,223268	62,607194	0,364829359
54	8	D	13	59,91	43,300003	48,937325	0,420006	75,069244	69,24	-3,55	52,93			16,869	9,18	11,982154	6,104	0,4441667	10,188616	28,157236	61,654148	0,969990695
54	8	D	14	61,331	44,167503	46,985664	0,415012	78,783325	65,47	-3,54	51,94			16,545	9,2	12,003154	5,983	0,4183333	9,7552624	26,97657	63,268168	0,939632019
54	8	D	15	65,341	44,285004	57,794853	0,380001	78,837746	61,66	1,79	57,63			16,194	9,24		7,528	0,5183333	11,521097	24,783826	63,695077	0,858080537

Supplementary Table S1. Raw data of egg quality parameters with ATOL (Animal Trait Ontology for Livestock) descriptors, for hen.

				ATOL_ 0001880	ATOL_ 0002297	ATOL_ 0002119	ATOL_ 0002126	ATOL_ 0002122	ATOL_ 0002074	ATOL_ 0002073	ATOL_ 0002075	ATOL_ 0005600	ATOL_ 0005600	ATOL_ 0001883	ATOL_ 0005587	ATOL_ 0001938	ATOL_ 1881	ATOL_ 0002121	ATOL_ 1881	ATOL_ 0001883	ATOL_ 0001882	ATOL_ 0002125
**Hen age**	**Pen**	**Genotype**	**Replicate**	**Egg weight**	**Egg diameter**	**Shell strength**	**Shell fracture**	**Shell modulus**	**Yolk lightness**	**Yolk redness**	**Yolk yellow**	**Blood spot**	**Meat spot**	**Yolk weight**	**Albumen pH**	**Albumen DM**	**Shell weight**	**Shell thickness**	**Shell**	**Yolk**	**Albumen**	**Shell-to-egg-compression**
wk	no.			g	mm	N	mm	N/mm	L*	a*	b*	1=present	1=present	g	-	% (w/w)	g	mm	% (w/w)	% (w/w)	% (w/w)	%
54	9	D	1	59,462	43,432503	46,68541	0,399986	75,054924	62,47	-1,44	48,08			15,998	9,2	11,84798	6,114	0,4583333	10,282197	26,904578	62,813225	0,920937023
54	9	D	2	62,085	44,349998	49,387707	0,419994	82,576172	57,58	-4,59	50,87			16,871	9,22	11,86798	6,196	0,4266667	9,9798663	27,174036	62,846098	0,946998915
54	9	D	3	65,872	46,065002	38,728645	0,467495	86,307129	56,36	-0,07	47,5			18,545	9,18	13,236269	5,528	0,3983333	8,392033	28,153085	63,454882	1,014859394
54	9	D	4	64,809	44,842499	57,494598	0,342491	97,590019	57,45	-1,41	46,18			15,81	9,2	13,256269	7,196	0,4858333	11,103396	24,39476	64,501844	0,763764303
54	9	D	5	60,631	43,702499	55,542938	0,454994	44,996532	56,15	-0,14	48,46			16,181	9,22	11,929078	6,489	0,4641667	10,702446	26,687668	62,609886	1,041116665
54	9	D	6	64,607	44,547501	45,184135	0,387493	67,562325	61,67	-4,64	53,09			16,559	9,26	11,971078	6,208	0,4216667	9,6088659	25,63035	64,760784	0,869842284
54	9	D	7	59,253	43,205002	55,843193	0,449997	71,308983	62,09	1,06	50,65			17,23	9,15	11,43805	6,51	0,485	10,986785	29,078696	59,934518	1,041539125
54	9	D	8	63,431	44,202499	51,339367	0,369995	78,815186						18,661	9,19	11,48005	6,271	0,4366667	9,8863332	29,419369	60,694298	0,837045435
54	9	D	9	61,787	43,332497	55,392811	0,387497	90,083092	62,55	-2,55	54,45			15,985	9,16	11,680783	6,939	0,4816667	11,230518	25,871138	62,898344	0,894241105
54	9	D	10	65,462	44,300003	43,53273	0,3675	75,062088	59,67	-2,67	53,31			18,271	9,2	11,722783	6,579	0,4416667	10,050105	27,910849	62,039046	0,82957105
54	9	D	11	63,69	45,364998	48,336815	0,422497	90,09465	56,91	-1,6	50,98			17,417	9,2	12,293828	5,893	0,4166667	9,2526299	27,346522	63,400848	0,931328157
54	9	D	12	62,459	44,422501	57,194344	0,459991	78,807671	62,85	-1,26	53,56			17,65	9,26	12,353828	6,018	0,4416667	9,6351206	28,258538	62,106342	1,035491
54	9	D	13	59,894	43,592499	42,331711	0,374992	75,069244	58,75	-1,21	46,98			16,683	9,18	11,513501	6,006	0,44	10,027716	27,854209	62,118075	0,860221388
54	9	D	14	64,788	44,449997	50,588726	0,392502	75,062088	59,61	-2,6	42,71			17,994	9,22	11,555501	6,728	0,45	10,384639	27,773662	61,841699	0,883019182
54	9	D	15	65,268	44,592499	54,191792	0,429989	82,576164	57,85	0,27	56,04		1	18,112	9,19		6,333	0,4416667	9,7030704	27,750199	62,54673	0,96426307
54	11	C	1	70,372	45,25	33,774437	0,502495		62,45	-3,46	49,56			17,841	9,17	12,566453	6,011	0,4025	8,5417496	25,352413	66,105838	1,110486188
54	11	C	2	61,471	44,892502	28,970352	0,332497	63,803234	62,46	-3,81	50,7	1		16,459	9,19	12,586453	4,97	0,3766667	8,0851133	26,775227	65,139659	0,740651523
54	11	C	3	68,312	45,157501	55,392811	0,382496	86,337875	60,11	1,93	56,78			18,097	9,21	12,762277	6,711	0,4416667	9,8240426	26,491685	63,684272	0,8470265
54	11	C	4	67,164	45,3825	34,825329	0,464993	71,296555	61,38	-3,84	51,37		1	18,486	9,23	12,782277	5,124	0,3658333	7,629087	27,523673	64,84724	1,024608605
54	11	C	5	58,49	43,330002	30,171373	0,502495	7,495672	64,72	-4,24	49,51			16,313	9,2	12,094259	5,494	0,4033333	9,3930586	27,890238	62,716704	1,159693
54	11	C	6	70,395	45,447502	39,329159	0,389999	78,815186	64,1	-3,7	51,37		1	19,089	9,24	12,136259	6,525	0,4216667	9,2691242	27,116983	63,613893	0,858130773
54	11	C	7	66,754	45,107498	47,436047	0,385006	82,584045	64,31	-1,32	60,1			18,783	9,17	11,68647	6,011	0,4116667	9,0047038	28,13764	62,857656	0,853529939
54	11	C	8	64,792	44,264999	42,181583	0,352497	78,800163	49,58	-1,25	58,56				9,21	11,72847	6,294	0,44	9,7141622			0,796333464
54	11	C	9	63,977	44,637497	37,978008	0,322502	86,313171	65,63	-3,86	49,49			17,596	9,22	11,927791	5,718	0,3933333	8,9375869	27,503634	63,558779	0,722491227
54	11	C	10	63,84	43,705002	34,675201	0,355	78,817093	59,8	-0,17	60,55			18,153	9,26	11,969791	5,423	0,3933333	8,4946742	28,43515	63,070175	0,812264006
54	11	C	11	65,958	44,9375	47,135792	0,39249	67,562325	65,83	-3,64	57,81			18,624	9,24	12,279293	6,775	0,4691667	10,271688	28,23615	61,492162	0,873413074
54	11	C	12	66,185	43,472504	36,026348	0,34499	78,815186	63,32	-3,83	51,75			19,145	9,3	12,339293	6,112	0,4116667	9,2347209	28,926494	61,838785	0,793582077
54	11	C	13	68,701	44,544998	50,888985	0,389999	78,837738	65,99	-2,58	57,5			19,033	9,2	12,225553	6,343	0,4283333	9,2327623	27,704109	63,063129	0,875516932
54	11	C	14	65,153	43,565002	42,181583	0,40749	67,549431	62,4	-4,85	53,13	1		18,803	9,24	12,267553	6,272	0,435	9,6265713	28,859761	61,513668	0,935360912
54	11	C	15	69,976	45,607498	37,527626	0,402481	93,827606	62,59	-3,05	47,2			18,237	9,18		5,969	0,4083333	8,5300675	26,061793	65,40814	0,882488664
54	12	B	1	71,213	46,195	43,382603	0,440002	105,09694	57,31	-2,5	48,23			20,974	9,16	11,726941	6,522	0,4116667	9,1584402	29,452488	61,389072	0,952488365
54	12	B	2	68,835	45,120003	25,9678	0,332493	63,768562	63,71	-1,21	61,57			18,919	9,18	11,746941	5,416	0,3591667	7,8680904	27,484565	64,647345	0,736908196
54	12	B	3	71,8	46,390007	34,224819	0,399982	97,599327	62,8	-2,14	57,76			19,842	9,2	12,892086	5,349	0,3633333	7,4498607	27,635097	64,915042	0,862215865
54	12	B	4	64,724	43,879997	38,878773	0,412498	71,296555	61,56	-2,63	54,12			18,884	9,22	12,912086	5,699	0,3958333	8,80508	29,176194	62,018726	0,940059317
54	12	B	5	65,406	44,904999	39,479286	0,399994	60,061119	59,15	-2,32	58,54		1	18,877	9,15	11,854	5,78	0,3941667	8,8371097	28,861267	62,301624	0,89075606
54	12	B	6	67,387	45,404999	46,985664	0,387497	93,818665	52,95	-1,29	69,49			18,493	9,19	11,896	6,206	0,4166667	9,2094914	27,442979	63,34753	0,853423651
54	12	B	7	70,528	45,864998	37,227371	0,424995	108,76776	62,18	-4,83	49,24			19,283	9,08	12,921932	5,99	0,4041667	8,4930808	27,340914	64,166005	0,926621647
54	12	B	8	73,027	45,972504	36,927116	0,372498	93,827606	62,17	-3,91	48,05			19,965	9,12	12,963932	5,704	0,3916667	7,8108097	27,339203	64,849987	0,810262587
54	12	B	9	64,559	44,400002	36,776989	0,37249	78,807678	55,22	-0,27	65,65			17,746	9,06	11,604738	5,263	0,3691667	8,1522328	27,488034	64,359733	0,838941404
54	12	B	10	70,002	42,942497	43,232475	0,377499	86,329628	61,52	-4,05	46,96	1	1	16,009	9,1	11,646738	6,833	0,4583333	9,7611497	22,869347	67,369504	0,879080227
54	12	B	11	66,422	44,474998	39,929668	0,397499	67,575203	64,96	-1,95	54,89			16,828	9,17	12,479012	5,709	0,3908333	8,5950438	25,334979	66,069977	0,893758331
54	12	B	12	67,592	44,932503	47,135792	0,374992	71,315781	59,9	-1,72	59,07			17,845	9,23	12,539012	6,609	0,4483333	9,7777844	26,401053	63,821162	0,834567351
54	12	B	13	59,638	42,489998	25,36729	0,307491	75,069244							9,17	12,981214	5,142	0,3716667	8,6220195			0,723678547
54	12	B	14	69,849	45,325005	47,285919	0,434994	71,30899							9,21	13,023214	6,187	0,4216667	8,8576787			0,959721902
54	12	B	15	65,896	45,43	40,380051	0,417496	97,580711	69,14	-2,56	57,12			18,633	9,2		5,555	0,3875	8,4299502	28,276375	63,293675	0,918987453
